# Supplementary material for: Homeostatic control of energy metabolism by monocyte-derived macrophages
Source: EMBO J. 2025 Nov 17;45(1):106–50. doi: 10.1038/s44318-025-00622-x (PMC12759084; doi:10.1038/s44318-025-00622-x)
Supplement: Supplementary file 1 — Appendix [file 44318_2025_622_MOESM1_ESM.pdf]

Appendix for:

Homeostatic control of energy metabolism by monocyte-derived macrophages

Contents

Appendix Figure S1 2

Appendix Figure S2 4

Appendix Figure S3. 6

Appendix Figure S4 8

Appendix Table S1.. 10

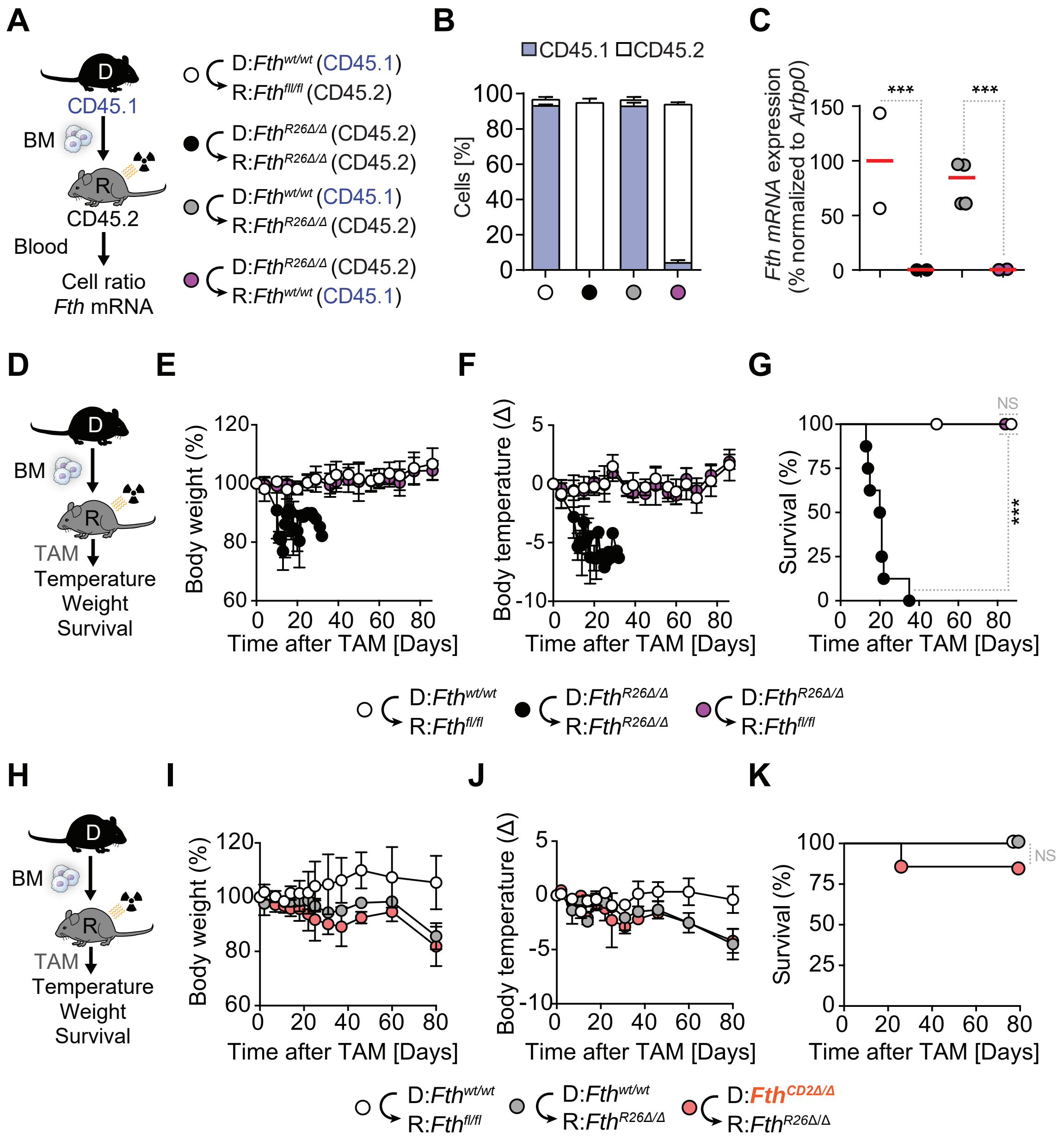

Appendix Figure S1: *Fth*-competent chimeric mice, rescue from whole-body *Fth* deletion-induced lethality independently of T-lymphocytes. (**A**) Schematic representation of chimeric mice and engraftment assessment. (**B**) Relative immune cell percentage of donor or recipient origin and (**C**) corresponding *Fth* gene expression in the blood of $Fth^{wt/wt}$⇨$Fth^{wt/wt}$ (n=2), $Fth^{R26\Delta/\Delta}$⇨$Fth^{R26\Delta/\Delta}$ (n=2), $Fth^{wt/wt}$⇨$Fth^{R26\Delta/\Delta}$ (n=3) and $Fth^{R26\Delta/\Delta}$⇨$Fth^{wt/wt}$ (n=2) chimeric mice, collected on day 10 post-TAM administration, as determined via flow cytometry analysis (B) and qRT-PCR (C). (**D**) Schematic representation of TAM-induced *Fth* deletion in chimeric mice and monitoring vital parameters. Relative body weight (**E**), temperature (**F**) and survival (**G**) of $Fth^{fl/fl}$⇨$Fth^{fl/fl}$ (n=8), $Fth^{R26\Delta/\Delta}$⇨$Fth^{R26\Delta/\Delta}$ (n=8) and $Fth^{R26\Delta/\Delta}$⇨$Fth^{fl/fl}$ (n=8) chimeric mice following TAM administration on day 0. Data in (E, F) is represented as mean ± SD. Data in (E-G) is pooled from 3 experiments. (**H**) Schematic representation of TAM-induced *Fth* deletion in chimeric mice and monitoring vital parameters. Relative body weight (**I**), temperature (**J**)

and survival (**K**) of $Fth^{wt/wt}$⇨$Fth^{fl/fl}$ (n=3), $Fth^{wt/wt}$⇨$Fth^{R26\Delta/\Delta}$ (n=3) and $Fth^{CD2\Delta/\Delta}$⇨ $Fth^{R26\Delta/\Delta}$ (n=7) chimeric mice following TAM administration on day 0. Data in (I, J) is represented as mean ± SD. Data in (I-K) is pooled from 2 experiments. One-Way ANOVA with Tukey's range test for multiple comparison correction was used for comparison between multiple groups. Survival analysis was performed using Log-rank (Mantel-Cox) test. NS: non-significant, *** P<0.001.

Appendix Figure S2

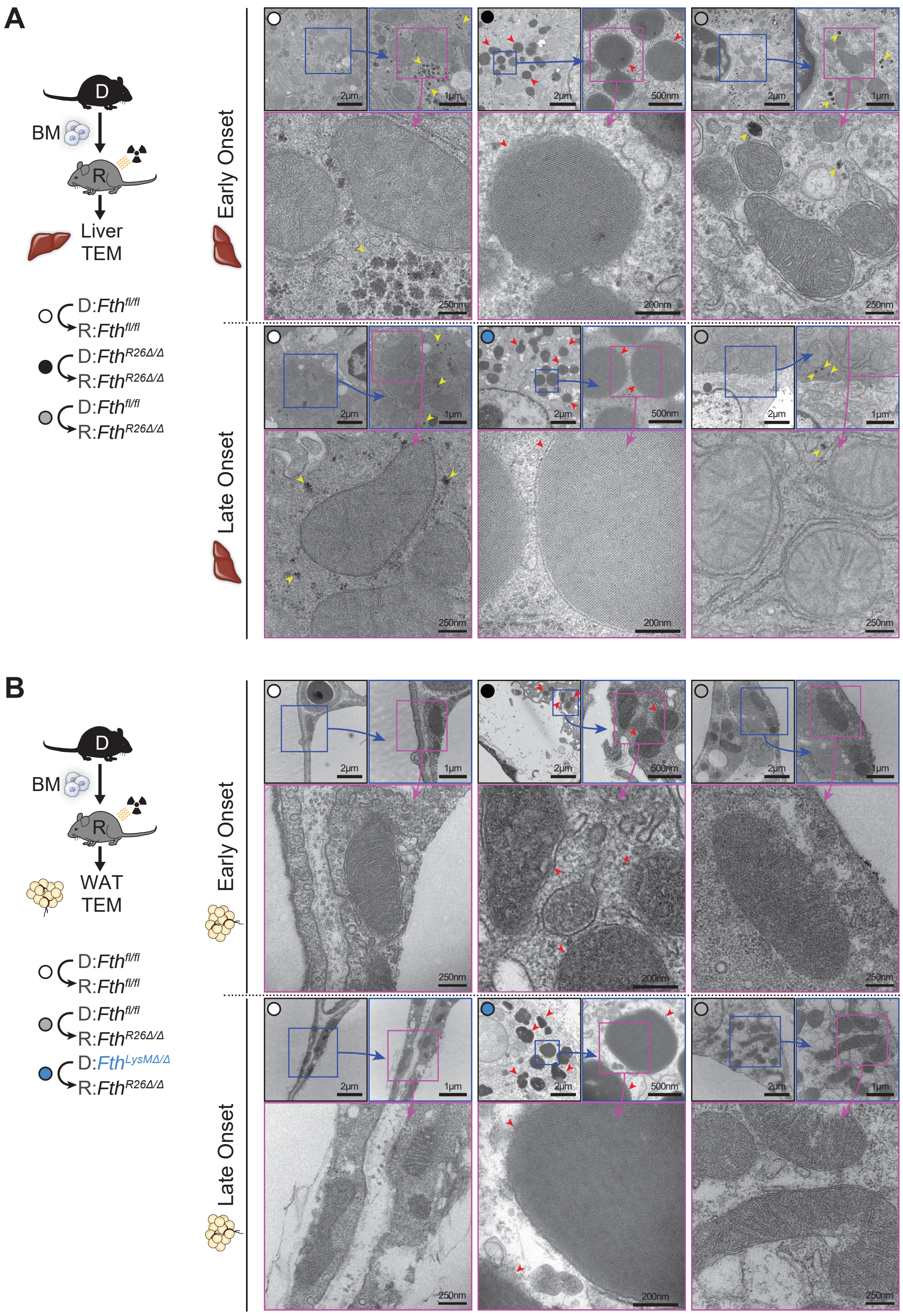

Appendix Figure S2: *Fth*-competent myeloid cells prevent the formation of iron-filled siderosomes in chimeric *Fth*-deleted mice. Schematic representation of chimeric mice and TAM-induced *Fth* deletion (day 0) and representative transmission electron microscopy images of siderosome-like structures present in (**A**) hepatocytes (liver) and (**B**) adipocytes (gWAT) from $Fth^{R26\Delta/\Delta}$⇨$Fth^{R26\Delta/\Delta}$ chimeric mice, collected on

day 8 (early onset), and $Fth^{LysM\Delta/\Delta}$⇨$Fth^{R26\Delta/\Delta}$ chimeric mice, collected on day 30 (late onset), but absent in either $Fth^{fl/fl}$⇨$Fth^{fl/fl}$ or $Fth^{fl/fl}$⇨$Fth^{R26\Delta/\Delta}$ chimeric mice, at either time points. Red arrows indicate membrane-bound, electron-dense, siderosome-like structures filled with apparent crystalline iron, consistent with earlier descriptions (Sato, Ogihara et al., 1978). Yellow arrows indicate hepatic glycogen granules. Data in (A, B) is representative of 2 independent experiments with similar trends.

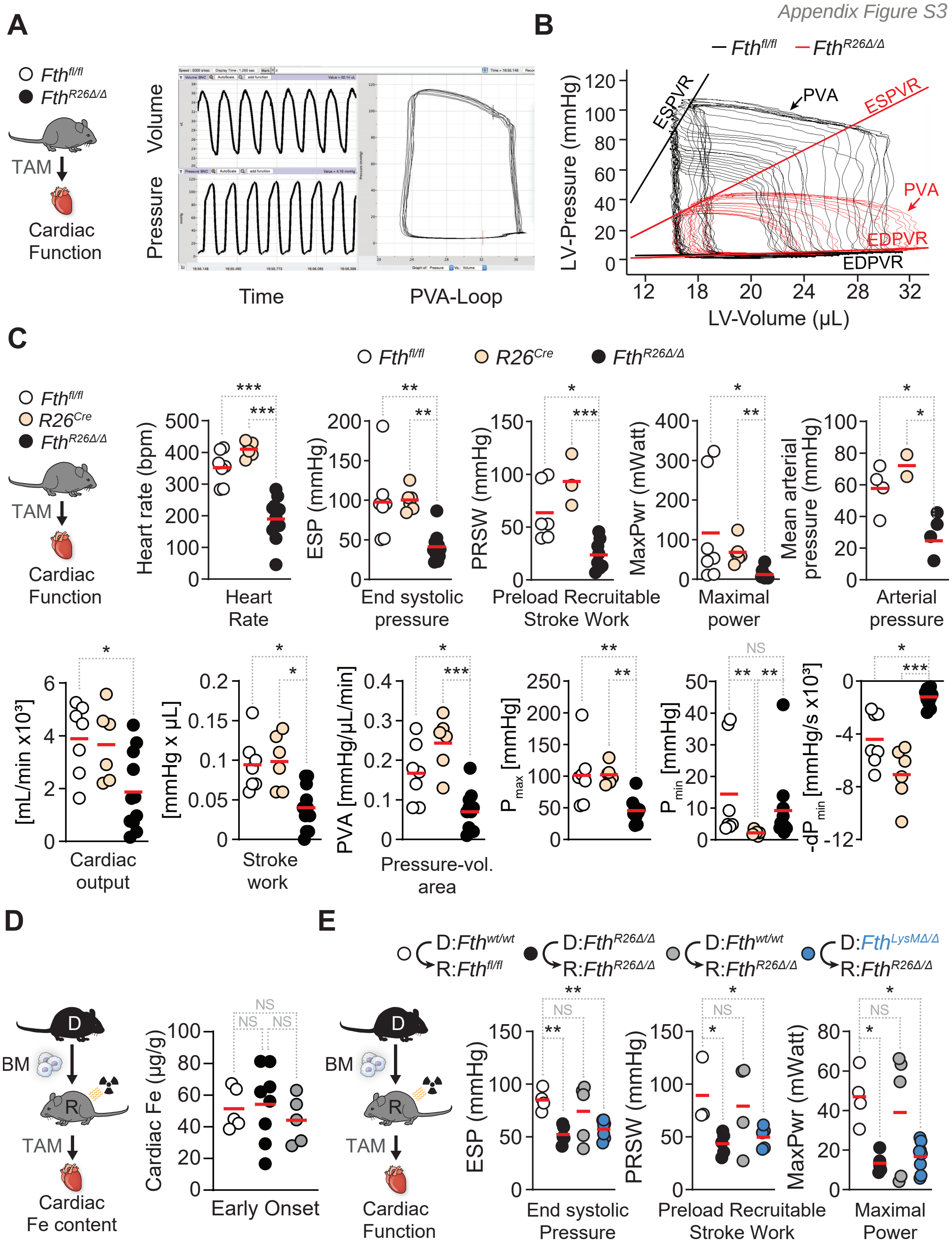

Appendix Figure S3: *Fth*-competent myeloid cells support tissue function in chimeric *Fth*-deleted mice. (**A**) Schematic representation of TAM-induced *Fth* deletion, cardiac function assessment and pressure volume loop analysis (PVA loop). (**B**) PVA loop analysis of $Fth^{fl/fl}$ (n=7) and $Fth^{R26\Delta/\Delta}$ (n=11) mice, on day **7** post-TAM administration. ESPVR = end systolic pressure-volume relationship, EDPVR = end diastolic pressure-volume relationship. (**C**) Schematic representation of TAM-induced *Fth* deletion (day 0) and cardiac function parameter quantification of $Fth^{fl/fl}$ (n=7), $R26^{Cre}$ (n=6) and $Fth^{R26\Delta/\Delta}$ (n=11) mice, on

day 7 post-TAM administration. Data represented as individual values (circles) and mean (red bars). (**D**) Cardiac iron content in $Fth^{fl/fl}$⇨$Fth^{fl/fl}$ (n=3-5), $Fth^{R26\Delta/\Delta}$⇨$Fth^{R26\Delta/\Delta}$ (n=8) and $Fth^{fl/fl}$⇨$Fth^{R26\Delta/\Delta}$ (n=3-5) chimeric mice, 7 days (early onset) following TAM administration. Data pooled from 3 independent experiments with similar trends. (**E**) Schematic representation of chimeric mice and TAM-induced *Fth* deletion (day 0) and cardiac function parameters (end systolic pressure; preload recruitable stroke work; maximal power) in $Fth^{fl/fl}$⇨$Fth^{fl/fl}$ (n=4), $Fth^{R26\Delta/\Delta}$⇨$Fth^{R26\Delta/\Delta}$ (n=5), $Fth^{fl/fl}$⇨$Fth^{R26\Delta/\Delta}$ (n=5) and $Fth^{LysM\Delta/\Delta}$⇨$Fth^{R26\Delta/\Delta}$ (n=8) chimeric mice between days 10-51 following TAM administration. Data pooled from 2 independent experiments with similar trends. Data in (C-E) presented as individual values (circles) and mean (red bars). One-Way ANOVA with Tukey's range test for multiple comparison correction was used for comparison between multiple groups. NS: non-significant, * P<0.05, ** P<0.01, *** P<0.001.

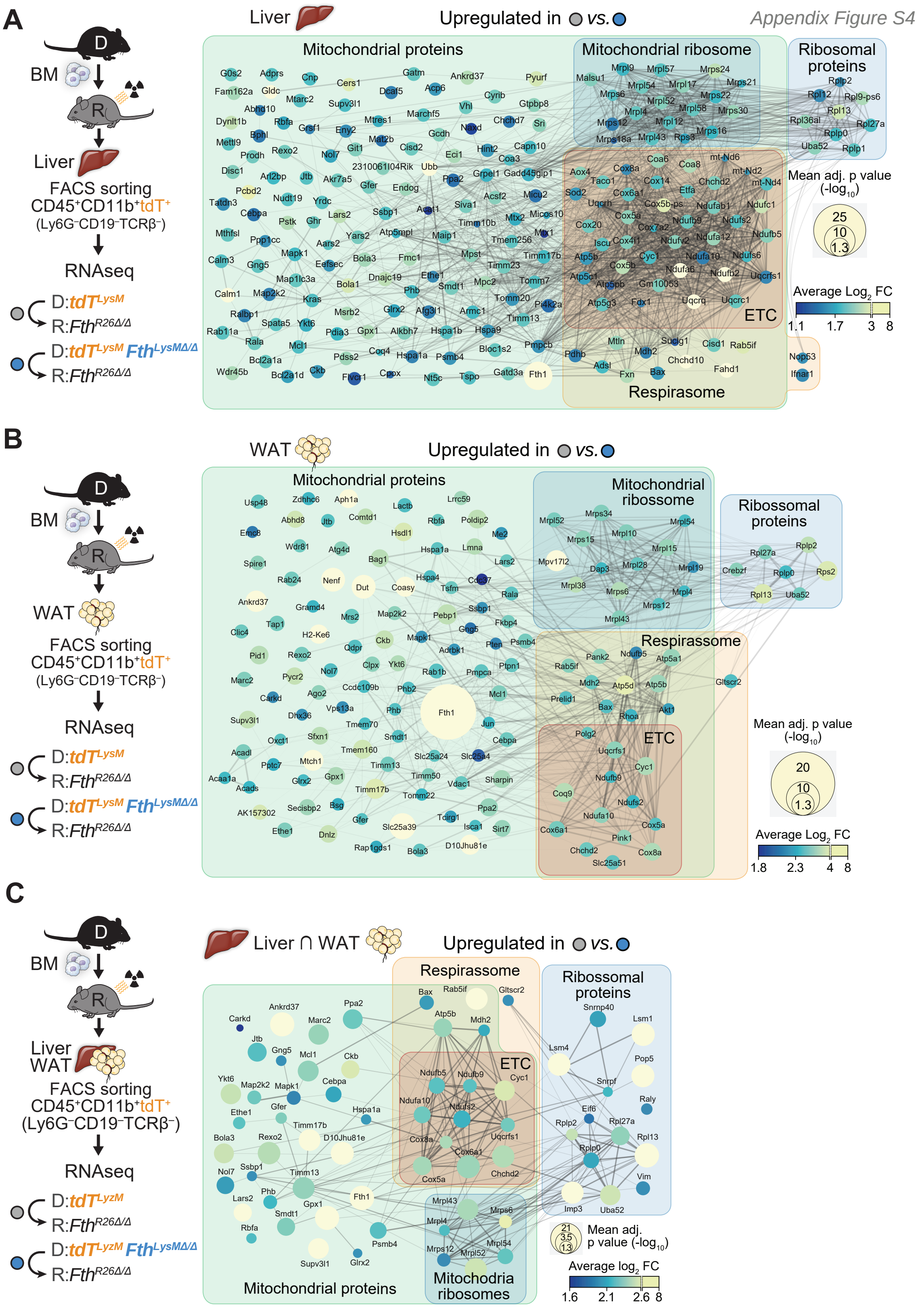

Appendix Figure S4: Mitochondrial gene expression program upregulated by *Fth*-competent monocyte-derived macrophages in *Fth*-deleted chimeras. (**A-C**) Schematic representations of chimeric mice, TAM administration and fluorescence-activated cell sorting (FACS) of *LysM*+

monocyte/macrophages ($CD45^+$,$CD11b^+$,$Ly6G^-$,$CD19^-$,$TCR\beta^-$) and STRING database (STRING-DB) interaction networks of mitochondrial, and mitochondria-related genes (as per gene ontology analysis) significantly upregulated in $LysM^+$ monocyte-derived macrophages from tdT^{LysM}⇨$Fth^{R26\Delta/\Delta}$ chimeric mice, as compared to $tdT^{LysM}Fth^{LysM\Delta/\Delta}$⇨$Fth^{R26\Delta/\Delta}$ chimeric mice in either (**A**) liver or (**B**) WAT. (**C**) Mitochondrial and mitochondria-related genes that are significantly upregulated in $LysM^+$ monocyte-derived macrophages from both the liver and WAT (intersection of significantly upregulated genes) of tdT^{LysM}⇨$Fth^{R26\Delta/\Delta}$ chimeric mice, as compared to $tdT^{LysM}Fth^{LysM\Delta/\Delta}$⇨$Fth^{R26\Delta/\Delta}$ chimeric mice. In (A-C), known interactions annotated in the STRING-DB are depicted by edges connecting gene dots.

Appendix Table S1

Figure	Panel	Comparison			Note	Stars	p-value
1	D	$Fth^{LysM\Delta/\Delta} \rightarrow Fth^{R26\Delta/\Delta}$	vs.	$Fth^{R26\Delta/\Delta} \rightarrow Fth^{R26\Delta/\Delta}$	Survival	***	0,0003
		$Fth^{fl/fl} \rightarrow Fth^{R26\Delta/\Delta}$	vs.	$Fth^{R26\Delta/\Delta} \rightarrow Fth^{R26\Delta/\Delta}$	Survival	****	<0,0001
		$Fth^{wt/wt} \rightarrow Fth^{fl/fl}$	vs.	$Fth^{R26\Delta/\Delta} \rightarrow Fth^{R26\Delta/\Delta}$	Survival	****	<0,0001
		$Fth^{fl/fl} \rightarrow Fth^{R26\Delta/\Delta}$		$Fth^{LysM\Delta/\Delta} \rightarrow Fth^{R26\Delta/\Delta}$	Survival	****	<0,0001
		$Fth^{wt/wt} \rightarrow Fth^{fl/fl}$	vs.	$Fth^{fl/fl} \rightarrow Fth^{R26\Delta/\Delta}$	Survival	NS	0,273
	F	$Fth^{wt/wt} \rightarrow Fth^{fl/fl}$	vs.	$Fth^{R26\Delta/\Delta} \rightarrow Fth^{R26\Delta/\Delta}$	Liver	**	0,0014
		$Fth^{fl/fl} \rightarrow Fth^{R26\Delta/\Delta}$	vs.	$Fth^{R26\Delta/\Delta} \rightarrow Fth^{R26\Delta/\Delta}$	Liver	***	0,0005
		$Fth^{wt/wt} \rightarrow Fth^{fl/fl}$	vs.	$Fth^{fl/fl} \rightarrow Fth^{R26\Delta/\Delta}$	Liver	NS	0,9656
		$Fth^{wt/wt} \rightarrow Fth^{fl/fl}$	vs.	$Fth^{LysM\Delta/\Delta} \rightarrow Fth^{R26\Delta/\Delta}$	Liver	**	0,001
		$Fth^{fl/fl} \rightarrow Fth^{R26\Delta/\Delta}$	vs.	$Fth^{LysM\Delta/\Delta} \rightarrow Fth^{R26\Delta/\Delta}$	Liver	***	0,0004
		$Fth^{wt/wt} \rightarrow Fth^{fl/fl}$	vs.	$Fth^{R26\Delta/\Delta} \rightarrow Fth^{R26\Delta/\Delta}$	Heart	NS	0,0807
		$Fth^{fl/fl} \rightarrow Fth^{R26\Delta/\Delta}$	vs.	$Fth^{R26\Delta/\Delta} \rightarrow Fth^{R26\Delta/\Delta}$	Heart	**	0,0012
		$Fth^{wt/wt} \rightarrow Fth^{fl/fl}$	vs.	$Fth^{fl/fl} \rightarrow Fth^{R26\Delta/\Delta}$	Heart	NS	0,1918
		$Fth^{wt/wt} \rightarrow Fth^{fl/fl}$	vs.	$Fth^{LysM\Delta/\Delta} \rightarrow Fth^{R26\Delta/\Delta}$	Heart	*	0,0425
		$Fth^{fl/fl} \rightarrow Fth^{R26\Delta/\Delta}$	vs.	$Fth^{LysM\Delta/\Delta} \rightarrow Fth^{R26\Delta/\Delta}$	Heart	***	0,0004
		$Fth^{wt/wt} \rightarrow Fth^{fl/fl}$	vs.	$Fth^{R26\Delta/\Delta} \rightarrow Fth^{R26\Delta/\Delta}$	Lungs	*	0,0104
		$Fth^{fl/fl} \rightarrow Fth^{R26\Delta/\Delta}$	vs.	$Fth^{R26\Delta/\Delta} \rightarrow Fth^{R26\Delta/\Delta}$	Lungs	**	0,0049
		$Fth^{wt/wt} \rightarrow Fth^{fl/fl}$	vs.	$Fth^{fl/fl} \rightarrow Fth^{R26\Delta/\Delta}$	Lungs	NS	0,9841
		$Fth^{wt/wt} \rightarrow Fth^{fl/fl}$	vs.	$Fth^{LysM\Delta/\Delta} \rightarrow Fth^{R26\Delta/\Delta}$	Lungs	**	0,0094
		$Fth^{fl/fl} \rightarrow Fth^{R26\Delta/\Delta}$	vs.	$Fth^{LysM\Delta/\Delta} \rightarrow Fth^{R26\Delta/\Delta}$	Lungs	**	0,0043
		$Fth^{wt/wt} \rightarrow Fth^{fl/fl}$	vs.	$Fth^{R26\Delta/\Delta} \rightarrow Fth^{R26\Delta/\Delta}$	Kidneys	***	0,0004
		$Fth^{fl/fl} \rightarrow Fth^{R26\Delta/\Delta}$	vs.	$Fth^{R26\Delta/\Delta} \rightarrow Fth^{R26\Delta/\Delta}$	Kidneys	*	0,0497
		$Fth^{wt/wt} \rightarrow Fth^{fl/fl}$	vs.	$Fth^{fl/fl} \rightarrow Fth^{R26\Delta/\Delta}$	Kidneys	NS	0,1795
		$Fth^{wt/wt} \rightarrow Fth^{fl/fl}$	vs.	$Fth^{LysM\Delta/\Delta} \rightarrow Fth^{R26\Delta/\Delta}$	Kidneys	***	0,0003
		$Fth^{fl/fl} \rightarrow Fth^{R26\Delta/\Delta}$	vs.	$Fth^{LysM\Delta/\Delta} \rightarrow Fth^{R26\Delta/\Delta}$	Kidneys	*	0,0495
	G	$Fth^{LysM\Delta/\Delta} \rightarrow Fth^{R26\Delta/\Delta}$	vs.	$Fth^{R26\Delta/\Delta} \rightarrow Fth^{R26\Delta/\Delta}$	Parabiosis survival	NS	0,1119
		$Fth^{fl/fl} \rightarrow Fth^{R26\Delta/\Delta}$	vs.	$Fth^{R26\Delta/\Delta} \rightarrow Fth^{R26\Delta/\Delta}$	Parabiosis survival	***	0,0007
		$Fth^{fl/fl} \rightarrow Fth^{fl/fl}$	vs.	$Fth^{R26\Delta/\Delta} \rightarrow Fth^{R26\Delta/\Delta}$	Parabiosis survival	**	0,0014
		$Fth^{fl/fl} \rightarrow Fth^{fl/fl}$	vs.	$Fth^{fl/fl} \rightarrow Fth^{R26\Delta/\Delta}$	Parabiosis survival	NS	0,5165
		$Fth^{fl/fl} \rightarrow Fth^{fl/fl}$	vs.	$Fth^{LysM\Delta/\Delta} \rightarrow Fth^{R26\Delta/\Delta}$	Parabiosis survival	*	0,0294
		$Fth^{LysM\Delta/\Delta} \rightarrow Fth^{R26\Delta/\Delta}$	vs.	$Fth^{fl/fl} \rightarrow Fth^{R26\Delta/\Delta}$	Parabiosis survival	*	0,0376
	I	$tdT^{R26} \rightarrow Fth^{fl/fl}$	vs.	$tdT^{R26} Fth^{R26\Delta/\Delta} \rightarrow Fth^{R26\Delta/\Delta}$	Survival	**	0,0026
		$tdT^{R26} \rightarrow Fth^{R26\Delta/\Delta}$	vs.	$tdT^{R26} Fth^{R26\Delta/\Delta} \rightarrow Fth^{R26\Delta/\Delta}$	Survival	*	0,0203
		$tdT^{R26} \rightarrow Fth^{fl/fl}$	vs.	$tdT^{R26} \rightarrow Fth^{R26\Delta/\Delta}$	Survival	**	0,0021
	J	$Fth^{wt/wt} \rightarrow Fth^{fl/fl}$	vs.	$Fth^{LysM\Delta/\Delta} \rightarrow Fth^{R26\Delta/\Delta}$	Liver	***	0,0009
		$Fth^{fl/fl} \rightarrow Fth^{R26\Delta/\Delta}$	vs.	$Fth^{LysM\Delta/\Delta} \rightarrow Fth^{R26\Delta/\Delta}$	Liver	****	<0,0001
		$Fth^{wt/wt} \rightarrow Fth^{fl/fl}$	vs.	$Fth^{fl/fl} \rightarrow Fth^{R26\Delta/\Delta}$	Liver	*	0,0395
		$Fth^{wt/wt} \rightarrow Fth^{fl/fl}$	vs.	$Fth^{LysM\Delta/\Delta} \rightarrow Fth^{R26\Delta/\Delta}$	Spleen	**	0,008
		$Fth^{fl/fl} \rightarrow Fth^{R26\Delta/\Delta}$	vs.	$Fth^{LysM\Delta/\Delta} \rightarrow Fth^{R26\Delta/\Delta}$	Spleen	*	0,0123
		$Fth^{wt/wt} \rightarrow Fth^{fl/fl}$	vs.	$Fth^{fl/fl} \rightarrow Fth^{R26\Delta/\Delta}$	Spleen	NS	0,924

Figure	Panel	Comparison			Note	Stars	p-value
2	B	$Fth^{wt/wt} \rightarrow Fth^{fl/fl}$	vs.	$Fth^{R26\Delta/\Delta} \rightarrow Fth^{R26\Delta/\Delta}$	Early onset	****	<0,0001
		$Fth^{fl/fl} \rightarrow Fth^{R26\Delta/\Delta}$	vs.	$Fth^{R26\Delta/\Delta} \rightarrow Fth^{R26\Delta/\Delta}$	Early onset	**	0,0029
		$Fth^{wt/wt} \rightarrow Fth^{fl/fl}$	vs.	$Fth^{fl/fl} \rightarrow Fth^{R26\Delta/\Delta}$	Early onset	NS	0,079
		$Fth^{wt/wt} \rightarrow Fth^{fl/fl}$	vs.	$Fth^{LysM\Delta/\Delta} \rightarrow Fth^{R26\Delta/\Delta}$	Late onset	****	<0,0001
		$Fth^{fl/fl} \rightarrow Fth^{R26\Delta/\Delta}$	vs.	$Fth^{LysM\Delta/\Delta} \rightarrow Fth^{R26\Delta/\Delta}$	Late onset	**	0,0022
		$Fth^{wt/wt} \rightarrow Fth^{fl/fl}$	vs.	$Fth^{fl/fl} \rightarrow Fth^{R26\Delta/\Delta}$	Late onset	NS	0,1662
	C	$Fth^{wt/wt} \rightarrow Fth^{fl/fl}$	vs.	$Fth^{R26\Delta/\Delta} \rightarrow Fth^{R26\Delta/\Delta}$	Early onset	****	<0,0001
		$Fth^{fl/fl} \rightarrow Fth^{R26\Delta/\Delta}$	vs.	$Fth^{R26\Delta/\Delta} \rightarrow Fth^{R26\Delta/\Delta}$	Early onset	***	0,0004
		$Fth^{wt/wt} \rightarrow Fth^{fl/fl}$	vs.	$Fth^{fl/fl} \rightarrow Fth^{R26\Delta/\Delta}$	Early onset	NS	0,8493
		$Fth^{wt/wt} \rightarrow Fth^{fl/fl}$	vs.	$Fth^{LysM\Delta/\Delta} \rightarrow Fth^{R26\Delta/\Delta}$	Late onset	**	0,0022
		$Fth^{fl/fl} \rightarrow Fth^{R26\Delta/\Delta}$	vs.	$Fth^{LysM\Delta/\Delta} \rightarrow Fth^{R26\Delta/\Delta}$	Late onset	NS	0,174
		$Fth^{wt/wt} \rightarrow Fth^{fl/fl}$	vs.	$Fth^{fl/fl} \rightarrow Fth^{R26\Delta/\Delta}$	Late onset	NS	0,1915
	E	$Fth^{wt/wt} \rightarrow Fth^{fl/fl}$	vs.	$Fth^{R26\Delta/\Delta} \rightarrow Fth^{R26\Delta/\Delta}$	Early onset	NS	0,9991
		$Fth^{fl/fl} \rightarrow Fth^{R26\Delta/\Delta}$	vs.	$Fth^{R26\Delta/\Delta} \rightarrow Fth^{R26\Delta/\Delta}$	Early onset	NS	0,952
		$Fth^{wt/wt} \rightarrow Fth^{fl/fl}$	vs.	$Fth^{fl/fl} \rightarrow Fth^{R26\Delta/\Delta}$	Early onset	NS	0,9322
		$Fth^{wt/wt} \rightarrow Fth^{fl/fl}$	vs.	$Fth^{LysM\Delta/\Delta} \rightarrow Fth^{R26\Delta/\Delta}$	Late onset	***	0,0008
		$Fth^{fl/fl} \rightarrow Fth^{R26\Delta/\Delta}$	vs.	$Fth^{LysM\Delta/\Delta} \rightarrow Fth^{R26\Delta/\Delta}$	Late onset	NS	0,9844
		$Fth^{wt/wt} \rightarrow Fth^{fl/fl}$	vs.	$Fth^{fl/fl} \rightarrow Fth^{R26\Delta/\Delta}$	Late onset	**	0,0047
	F	$Fth^{fl/fl} \rightarrow Fth^{fl/fl}$	vs.	$Fth^{R26\Delta/\Delta} \rightarrow Fth^{R26\Delta/\Delta}$	Early onset	NS	0,1197
		$Fth^{fl/fl} \rightarrow Fth^{R26\Delta/\Delta}$	vs.	$Fth^{R26\Delta/\Delta} \rightarrow Fth^{R26\Delta/\Delta}$	Early onset	NS	0,0575
		$Fth^{fl/fl} \rightarrow Fth^{fl/fl}$	vs.	$Fth^{fl/fl} \rightarrow Fth^{R26\Delta/\Delta}$	Early onset	**	0,0022
		$Fth^{fl/fl} \rightarrow Fth^{fl/fl}$	vs.	$Fth^{LysM\Delta/\Delta} \rightarrow Fth^{R26\Delta/\Delta}$	Late onset	***	0,0005
		$Fth^{fl/fl} \rightarrow Fth^{R26\Delta/\Delta}$	vs.	$Fth^{LysM\Delta/\Delta} \rightarrow Fth^{R26\Delta/\Delta}$	Late onset	NS	0,9423
		$Fth^{fl/fl} \rightarrow Fth^{fl/fl}$	vs.	$Fth^{fl/fl} \rightarrow Fth^{R26\Delta/\Delta}$	Late onset	**	0,0024
		$Fth^{wt/wt} \rightarrow OKD48^{Luc} Fth^{fl/fl}$	vs.	$Fth^{R26M\Delta/\Delta} \rightarrow OKD48^{Luc} Fth^{R26\Delta/\Delta}$	Early onset	**	0,0089

	G	$Fth^{fl/fl}$ → $OKD48^{Luc}$ $Fth^{R26\Delta/\Delta}$	vs.	$Fth^{R26M\Delta/\Delta}$ → $OKD48^{Luc}$ $Fth^{R26\Delta/\Delta}$	Early onset	*	0,0237
		$Fth^{wt/wt}$ → $OKD48^{Luc}$ $Fth^{fl/fl}$	vs.	$Fth^{fl/fl}$ → $OKD48^{Luc}$ $Fth^{R26\Delta/\Delta}$	Early onset	NS	0,9143
	H	$Fth^{wt/wt}$ → $OKD48^{Luc}$ $Fth^{fl/fl}$	vs.	$Fth^{LysM\Delta/\Delta}$ → $OKD48^{Luc}$ $Fth^{R26\Delta/\Delta}$	Late onset	**	0,0041
		$Fth^{fl/fl}$ → $OKD48^{Luc}$ $Fth^{R26\Delta/\Delta}$	vs.	$Fth^{LysM\Delta/\Delta}$ → $OKD48^{Luc}$ $Fth^{R26\Delta/\Delta}$	Late onset	**	0,0065
		$Fth^{wt/wt}$ → $OKD48^{Luc}$ $Fth^{fl/fl}$	vs.	$Fth^{fl/fl}$ → $OKD48^{Luc}$ $Fth^{R26\Delta/\Delta}$	Late onset	NS	0,6837
	I	$Fth^{fl/fl}$ → $Fth^{fl/fl}$	vs.	$Fth^{LysM\Delta/\Delta}$ → $Fth^{R26\Delta/\Delta}$	Late onset	**	0,008
		$Fth^{fl/fl}$ → $Fth^{R26\Delta/\Delta}$	vs.	$Fth^{LysM\Delta/\Delta}$ → $Fth^{R26\Delta/\Delta}$	Late onset	*	0,0146
		$Fth^{fl/fl}$ → $Fth^{fl/fl}$	vs.	$Fth^{fl/fl}$ → $Fth^{R26\Delta/\Delta}$	Late onset	NS	0,9357
	J	$Fth^{wt/wt}$ → $Fth^{fl/fl}$	vs.	$Fth^{fl/fl}$ → $Fth^{R26\Delta/\Delta}$	Heart rate	NS	0,9993
		$Fth^{wt/wt}$ → $Fth^{fl/fl}$	vs.	$Fth^{R26\Delta/\Delta}$ → $Fth^{R26\Delta/\Delta}$	Heart rate	NS	0,0819
		$Fth^{wt/wt}$ → $Fth^{fl/fl}$	vs.	$Fth^{LysM\Delta/\Delta}$ → $Fth^{R26\Delta/\Delta}$	Heart rate	*	0,0219
		$Fth^{wt/wt}$ → $Fth^{fl/fl}$	vs.	$Fth^{fl/fl}$ → $Fth^{R26\Delta/\Delta}$	Mean arterial pressure	NS	0,8667
		$Fth^{wt/wt}$ → $Fth^{fl/fl}$	vs.	$Fth^{R26\Delta/\Delta}$ → $Fth^{R26\Delta/\Delta}$	Mean arterial pressure	*	0,0494
		$Fth^{wt/wt}$ → $Fth^{fl/fl}$	vs.	$Fth^{LysM\Delta/\Delta}$ → $Fth^{R26\Delta/\Delta}$	Mean arterial pressure	**	0,0032

Figure	Panel	Comparison			Note	Stars	p-value
3	B	$Fth^{fl/fl}$ → $Fth^{fl/fl}$	vs.	$Fth^{R26\Delta/\Delta}$ → $Fth^{R26\Delta/\Delta}$	Light	****	<0,0001
		$Fth^{fl/fl}$ → $Fth^{R26\Delta/\Delta}$	vs.	$Fth^{R26\Delta/\Delta}$ → $Fth^{R26\Delta/\Delta}$	Light	****	<0,0001
		$Fth^{fl/fl}$ → $Fth^{fl/fl}$	vs.	$Fth^{fl/fl}$ → $Fth^{R26\Delta/\Delta}$	Light	NS	0,9751
		$Fth^{fl/fl}$ → $Fth^{fl/fl}$	vs.	$Fth^{R26\Delta/\Delta}$ → $Fth^{R26\Delta/\Delta}$	Dark	****	<0,0001
		$Fth^{fl/fl}$ → $Fth^{R26\Delta/\Delta}$	vs.	$Fth^{R26\Delta/\Delta}$ → $Fth^{R26\Delta/\Delta}$	Dark	****	<0,0001
		$Fth^{fl/fl}$ → $Fth^{fl/fl}$	vs.	$Fth^{fl/fl}$ → $Fth^{R26\Delta/\Delta}$	Dark	NS	0,9921
	C	$Fth^{fl/fl}$ → $Fth^{fl/fl}$	vs.	$Fth^{LysM\Delta/\Delta}$ → $Fth^{R26\Delta/\Delta}$	Light	***	0,0007
		$Fth^{fl/fl}$ → $Fth^{R26\Delta/\Delta}$	vs.	$Fth^{LysM\Delta/\Delta}$ → $Fth^{R26\Delta/\Delta}$	Light	**	0,001
		$Fth^{fl/fl}$ → $Fth^{fl/fl}$	vs.	$Fth^{fl/fl}$ → $Fth^{R26\Delta/\Delta}$	Light	NS	0,8404
		$Fth^{fl/fl}$ → $Fth^{fl/fl}$	vs.	$Fth^{LysM\Delta/\Delta}$ → $Fth^{R26\Delta/\Delta}$	Dark	****	<0,0001
		$Fth^{fl/fl}$ → $Fth^{R26\Delta/\Delta}$	vs.	$Fth^{LysM\Delta/\Delta}$ → $Fth^{R26\Delta/\Delta}$	Dark	***	0,0004
		$Fth^{fl/fl}$ → $Fth^{fl/fl}$	vs.	$Fth^{fl/fl}$ → $Fth^{R26\Delta/\Delta}$	Dark	NS	0,2309
	D	$Fth^{fl/fl}$ → $Fth^{fl/fl}$	vs.	$Fth^{R26\Delta/\Delta}$ → $Fth^{R26\Delta/\Delta}$	Light	****	<0,0001
		$Fth^{fl/fl}$ → $Fth^{R26\Delta/\Delta}$	vs.	$Fth^{R26\Delta/\Delta}$ → $Fth^{R26\Delta/\Delta}$	Light	****	<0,0001
		$Fth^{fl/fl}$ → $Fth^{fl/fl}$	vs.	$Fth^{fl/fl}$ → $Fth^{R26\Delta/\Delta}$	Light	NS	0,9816
		$Fth^{fl/fl}$ → $Fth^{fl/fl}$	vs.	$Fth^{R26\Delta/\Delta}$ → $Fth^{R26\Delta/\Delta}$	Dark	****	<0,0001
		$Fth^{fl/fl}$ → $Fth^{R26\Delta/\Delta}$	vs.	$Fth^{R26\Delta/\Delta}$ → $Fth^{R26\Delta/\Delta}$	Dark	****	<0,0001
		$Fth^{fl/fl}$ → $Fth^{fl/fl}$	vs.	$Fth^{fl/fl}$ → $Fth^{R26\Delta/\Delta}$	Dark	NS	0,9923
	E	$Fth^{fl/fl}$ → $Fth^{fl/fl}$	vs.	$Fth^{R26\Delta/\Delta}$ → $Fth^{R26\Delta/\Delta}$	Light	****	<0,0001
		$Fth^{fl/fl}$ → $Fth^{R26\Delta/\Delta}$	vs.	$Fth^{R26\Delta/\Delta}$ → $Fth^{R26\Delta/\Delta}$	Light	****	<0,0001
		$Fth^{fl/fl}$ → $Fth^{fl/fl}$	vs.	$Fth^{fl/fl}$ → $Fth^{R26\Delta/\Delta}$	Light	NS	0,9408
		$Fth^{fl/fl}$ → $Fth^{fl/fl}$	vs.	$Fth^{R26\Delta/\Delta}$ → $Fth^{R26\Delta/\Delta}$	Dark	****	<0,0001
		$Fth^{fl/fl}$ → $Fth^{R26\Delta/\Delta}$	vs.	$Fth^{R26\Delta/\Delta}$ → $Fth^{R26\Delta/\Delta}$	Dark	****	<0,0001
		$Fth^{fl/fl}$ → $Fth^{fl/fl}$	vs.	$Fth^{fl/fl}$ → $Fth^{R26\Delta/\Delta}$	Dark	NS	0,9917
	F	$Fth^{fl/fl}$ → $Fth^{fl/fl}$	vs.	$Fth^{LysM\Delta/\Delta}$ → $Fth^{R26\Delta/\Delta}$	Light	***	0,0005
		$Fth^{fl/fl}$ → $Fth^{R26\Delta/\Delta}$	vs.	$Fth^{LysM\Delta/\Delta}$ → $Fth^{R26\Delta/\Delta}$	Light	***	0,0008
		$Fth^{fl/fl}$ → $Fth^{fl/fl}$	vs.	$Fth^{fl/fl}$ → $Fth^{R26\Delta/\Delta}$	Light	NS	0,8227
		$Fth^{fl/fl}$ → $Fth^{fl/fl}$	vs.	$Fth^{LysM\Delta/\Delta}$ → $Fth^{R26\Delta/\Delta}$	Dark	****	<0,0001
		$Fth^{fl/fl}$ → $Fth^{R26\Delta/\Delta}$	vs.	$Fth^{LysM\Delta/\Delta}$ → $Fth^{R26\Delta/\Delta}$	Dark	***	0,0003
		$Fth^{fl/fl}$ → $Fth^{fl/fl}$	vs.	$Fth^{fl/fl}$ → $Fth^{R26\Delta/\Delta}$	Dark	NS	0,2522
	G	$Fth^{fl/fl}$ → $Fth^{fl/fl}$	vs.	$Fth^{LysM\Delta/\Delta}$ → $Fth^{R26\Delta/\Delta}$	Light	**	0,0023
		$Fth^{fl/fl}$ → $Fth^{R26\Delta/\Delta}$	vs.	$Fth^{LysM\Delta/\Delta}$ → $Fth^{R26\Delta/\Delta}$	Light	**	0,003
		$Fth^{fl/fl}$ → $Fth^{fl/fl}$	vs.	$Fth^{fl/fl}$ → $Fth^{R26\Delta/\Delta}$	Light	NS	0,9045
		$Fth^{fl/fl}$ → $Fth^{fl/fl}$	vs.	$Fth^{LysM\Delta/\Delta}$ → $Fth^{R26\Delta/\Delta}$	Dark	***	0,0002
		$Fth^{fl/fl}$ → $Fth^{R26\Delta/\Delta}$	vs.	$Fth^{LysM\Delta/\Delta}$ → $Fth^{R26\Delta/\Delta}$	Dark	**	0,0013
		$Fth^{fl/fl}$ → $Fth^{fl/fl}$	vs.	$Fth^{fl/fl}$ → $Fth^{R26\Delta/\Delta}$	Dark	NS	0,1845

Figure	Panel	Comparison			Note	Stars	p-value
	B	$Fth^{fl/fl}$ → $Fth^{fl/fl}$	vs.	$Fth^{R26\Delta/\Delta}$ → $Fth^{R26\Delta/\Delta}$	BAT	***	0,0005
		$Fth^{fl/fl}$ → $Fth^{R26\Delta/\Delta}$	vs.	$Fth^{R26\Delta/\Delta}$ → $Fth^{R26\Delta/\Delta}$	BAT	***	0,0002
		$Fth^{fl/fl}$ → $Fth^{fl/fl}$	vs.	$Fth^{fl/fl}$ → $Fth^{R26\Delta/\Delta}$	BAT	NS	0,9793
		$Fth^{fl/fl}$ → $Fth^{fl/fl}$	vs.	$Fth^{R26\Delta/\Delta}$ → $Fth^{R26\Delta/\Delta}$	Tail	NS	0,4348
		$Fth^{fl/fl}$ → $Fth^{R26\Delta/\Delta}$	vs.	$Fth^{R26\Delta/\Delta}$ → $Fth^{R26\Delta/\Delta}$	Tail	NS	0,619
		$Fth^{fl/fl}$ → $Fth^{fl/fl}$	vs.	$Fth^{fl/fl}$ → $Fth^{R26\Delta/\Delta}$	Tail	NS	0,9347
		$Fth^{fl/fl}$ → $Fth^{fl/fl}$	vs.	$Fth^{R26\Delta/\Delta}$ → $Fth^{R26\Delta/\Delta}$	Temperature delta	***	0,0002
		$Fth^{fl/fl}$ → $Fth^{R26\Delta/\Delta}$	vs.	$Fth^{R26\Delta/\Delta}$ → $Fth^{R26\Delta/\Delta}$	Temperature delta	****	<0,0001
		$Fth^{fl/fl}$ → $Fth^{fl/fl}$	vs.	$Fth^{fl/fl}$ → $Fth^{R26\Delta/\Delta}$	Temperature delta	NS	0,866
	D	$Fth^{fl/fl}$ → $Fth^{fl/fl}$	vs.	$Fth^{LysM\Delta/\Delta}$ → $Fth^{R26\Delta/\Delta}$	BAT	**	0,0085
		$Fth^{fl/fl}$ → $Fth^{R26\Delta/\Delta}$	vs.	$Fth^{LysM\Delta/\Delta}$ → $Fth^{R26\Delta/\Delta}$	BAT	*	0,0459
		$Fth^{fl/fl}$ → $Fth^{fl/fl}$	vs.	$Fth^{fl/fl}$ → $Fth^{R26\Delta/\Delta}$	BAT	NS	0,9964
		$Fth^{fl/fl}$ → $Fth^{fl/fl}$	vs.	$Fth^{LysM\Delta/\Delta}$ → $Fth^{R26\Delta/\Delta}$	Tail	*	0,0287
		$Fth^{fl/fl}$ → $Fth^{R26\Delta/\Delta}$	vs.	$Fth^{LysM\Delta/\Delta}$ → $Fth^{R26\Delta/\Delta}$	Tail	NS	0,4773

Figure	Panel	Comparison			Note	Stars	p-value
4		$Fth^{fl/fl} \rightarrow Fth^{fl/fl}$	vs.	$Fth^{fl/fl} \rightarrow Fth^{R26\Delta/\Delta}$	Tail	NS	0,5502
		$Fth^{fl/fl} \rightarrow Fth^{fl/fl}$	vs.	$Fth^{LysM\Delta/\Delta} \rightarrow Fth^{R26\Delta/\Delta}$	Temperature delta	*	0,0217
		$Fth^{fl/fl} \rightarrow Fth^{R26\Delta/\Delta}$	vs.	$Fth^{LysM\Delta/\Delta} \rightarrow Fth^{R26\Delta/\Delta}$	Temperature delta	*	0,0385
		$Fth^{fl/fl} \rightarrow Fth^{fl/fl}$	vs.	$Fth^{fl/fl} \rightarrow Fth^{R26\Delta/\Delta}$	Temperature delta	NS	0,9211
	F	$Fth^{fl/fl} \rightarrow Fth^{fl/fl}$	vs.	$Fth^{R26\Delta/\Delta} \rightarrow Fth^{R26\Delta/\Delta}$	gWAT - organ weight	****	<0,0001
		$Fth^{fl/fl} \rightarrow Fth^{R26\Delta/\Delta}$	vs.	$Fth^{R26\Delta/\Delta} \rightarrow Fth^{R26\Delta/\Delta}$	gWAT - organ weight	****	<0,0001
		$Fth^{fl/fl} \rightarrow Fth^{fl/fl}$	vs.	$Fth^{fl/fl} \rightarrow Fth^{R26\Delta/\Delta}$	gWAT - organ weight	NS	0,5741
		$Fth^{fl/fl} \rightarrow Fth^{fl/fl}$	vs.	$Fth^{R26\Delta/\Delta} \rightarrow Fth^{R26\Delta/\Delta}$	gWAT - Adipocyte area	**	0,0072
		$Fth^{fl/fl} \rightarrow Fth^{R26\Delta/\Delta}$	vs.	$Fth^{R26\Delta/\Delta} \rightarrow Fth^{R26\Delta/\Delta}$	gWAT - Adipocyte area	*	0,016
		$Fth^{fl/fl} \rightarrow Fth^{fl/fl}$	vs.	$Fth^{fl/fl} \rightarrow Fth^{R26\Delta/\Delta}$	gWAT - Adipocyte area	NS	0,8223
	H	$Fth^{fl/fl} \rightarrow Fth^{fl/fl}$	vs.	$Fth^{LysM\Delta/\Delta} \rightarrow Fth^{R26\Delta/\Delta}$	gWAT - organ weight	*	0,0205
		$Fth^{fl/fl} \rightarrow Fth^{R26\Delta/\Delta}$	vs.	$Fth^{LysM\Delta/\Delta} \rightarrow Fth^{R26\Delta/\Delta}$	gWAT - organ weight	**	0,0057
		$Fth^{fl/fl} \rightarrow Fth^{fl/fl}$	vs.	$Fth^{fl/fl} \rightarrow Fth^{R26\Delta/\Delta}$	gWAT - organ weight	NS	0,8223
		$Fth^{fl/fl} \rightarrow Fth^{fl/fl}$	vs.	$Fth^{LysM\Delta/\Delta} \rightarrow Fth^{R26\Delta/\Delta}$	gWAT - Adipocyte area	*	0,0234
		$Fth^{fl/fl} \rightarrow Fth^{R26\Delta/\Delta}$	vs.	$Fth^{LysM\Delta/\Delta} \rightarrow Fth^{R26\Delta/\Delta}$	gWAT - Adipocyte area	NS	0,0651
		$Fth^{fl/fl} \rightarrow Fth^{fl/fl}$	vs.	$Fth^{fl/fl} \rightarrow Fth^{R26\Delta/\Delta}$	gWAT - Adipocyte area	NS	0,958

Figure	Panel	Comparison			Note	Stars	p-value
5	B	$Fth^{fl/fl} \rightarrow Fth^{fl/fl}$	vs.	$Fth^{LysM\Delta/\Delta} \rightarrow Fth^{R26\Delta/\Delta}$	Heart	***	0,0001
		$Fth^{fl/fl} \rightarrow Fth^{R26\Delta/\Delta}$	vs.	$Fth^{LysM\Delta/\Delta} \rightarrow Fth^{R26\Delta/\Delta}$	Heart	NS	0,2372
		$Fth^{fl/fl} \rightarrow Fth^{fl/fl}$	vs.	$Fth^{fl/fl} \rightarrow Fth^{R26\Delta/\Delta}$	Heart	**	0,0013
		$Fth^{fl/fl} \rightarrow Fth^{fl/fl}$	vs.	$Fth^{LysM\Delta/\Delta} \rightarrow Fth^{R26\Delta/\Delta}$	Liver	****	<0,0001
		$Fth^{fl/fl} \rightarrow Fth^{R26\Delta/\Delta}$	vs.	$Fth^{LysM\Delta/\Delta} \rightarrow Fth^{R26\Delta/\Delta}$	Liver	*	0,0437
		$Fth^{fl/fl} \rightarrow Fth^{fl/fl}$	vs.	$Fth^{fl/fl} \rightarrow Fth^{R26\Delta/\Delta}$	Liver	**	0,0016
		$Fth^{fl/fl} \rightarrow Fth^{fl/fl}$	vs.	$Fth^{LysM\Delta/\Delta} \rightarrow Fth^{R26\Delta/\Delta}$	Kidney	***	0,0001
		$Fth^{fl/fl} \rightarrow Fth^{R26\Delta/\Delta}$	vs.	$Fth^{LysM\Delta/\Delta} \rightarrow Fth^{R26\Delta/\Delta}$	Kidney	****	<0,0001
		$Fth^{fl/fl} \rightarrow Fth^{fl/fl}$	vs.	$Fth^{fl/fl} \rightarrow Fth^{R26\Delta/\Delta}$	Kidney	*	0,0351
		$Fth^{fl/fl} \rightarrow Fth^{fl/fl}$	vs.	$Fth^{LysM\Delta/\Delta} \rightarrow Fth^{R26\Delta/\Delta}$	Lung	****	<0,0001
		$Fth^{fl/fl} \rightarrow Fth^{R26\Delta/\Delta}$	vs.	$Fth^{LysM\Delta/\Delta} \rightarrow Fth^{R26\Delta/\Delta}$	Lung	**	0,0095
		$Fth^{fl/fl} \rightarrow Fth^{fl/fl}$	vs.	$Fth^{fl/fl} \rightarrow Fth^{R26\Delta/\Delta}$	Lung	*	0,0149
	E	$Fth^{R26\Delta/\Delta} \rightarrow Fth^{R26\Delta/\Delta}$	vs.	$Fth^{v5/v5} \rightarrow Fth^{fl/fl}$	Survival	****	<0,0001
		$Fth^{R26\Delta/\Delta} \rightarrow Fth^{R26\Delta/\Delta}$	vs.	$Fth^{v5/v5} \rightarrow Fth^{R26\Delta/\Delta}$	Survival	****	<0,0001

Figure	Panel	Comparison			Note	Stars	p-value
6	B	$Fth^{fl/fl} \rightarrow Fth^{fl/fl}$	vs.	$Fth^{R26\Delta/\Delta} \rightarrow Fth^{R26\Delta/\Delta}$	Liver (Early onset)	NS	0,9987
		$Fth^{fl/fl} \rightarrow Fth^{R26\Delta/\Delta}$	vs.	$Fth^{R26\Delta/\Delta} \rightarrow Fth^{R26\Delta/\Delta}$	Liver (Early onset)	NS	0,7295
		$Fth^{fl/fl} \rightarrow Fth^{fl/fl}$	vs.	$Fth^{fl/fl} \rightarrow Fth^{R26\Delta/\Delta}$	Liver (Early onset)	NS	0,785
		$Fth^{fl/fl} \rightarrow Fth^{fl/fl}$	vs.	$Fth^{R26\Delta/\Delta} \rightarrow Fth^{R26\Delta/\Delta}$	WAT (Early onset)	NS	0,9007
		$Fth^{fl/fl} \rightarrow Fth^{R26\Delta/\Delta}$	vs.	$Fth^{R26\Delta/\Delta} \rightarrow Fth^{R26\Delta/\Delta}$	WAT (Early onset)	NS	0,8512
		$Fth^{fl/fl} \rightarrow Fth^{fl/fl}$	vs.	$Fth^{fl/fl} \rightarrow Fth^{R26\Delta/\Delta}$	WAT (Early onset)	NS	0,5386
		$Fth^{fl/fl} \rightarrow Fth^{fl/fl}$	vs.	$Fth^{R26\Delta/\Delta} \rightarrow Fth^{R26\Delta/\Delta}$	Heart (Early onset)	NS	0,9997
		$Fth^{fl/fl} \rightarrow Fth^{R26\Delta/\Delta}$	vs.	$Fth^{R26\Delta/\Delta} \rightarrow Fth^{R26\Delta/\Delta}$	Heart (Early onset)	NS	0,6524
		$Fth^{fl/fl} \rightarrow Fth^{fl/fl}$	vs.	$Fth^{fl/fl} \rightarrow Fth^{R26\Delta/\Delta}$	Heart (Early onset)	NS	0,6109
	C	$Fth^{fl/fl} \rightarrow Fth^{fl/fl}$	vs.	$Fth^{LysM\Delta/\Delta} \rightarrow Fth^{R26\Delta/\Delta}$	Liver (Late onset)	NS	0,9338
		$Fth^{fl/fl} \rightarrow Fth^{R26\Delta/\Delta}$	vs.	$Fth^{LysM\Delta/\Delta} \rightarrow Fth^{R26\Delta/\Delta}$	Liver (Late onset)	NS	0,7696
		$Fth^{fl/fl} \rightarrow Fth^{fl/fl}$	vs.	$Fth^{fl/fl} \rightarrow Fth^{R26\Delta/\Delta}$	Liver (Late onset)	NS	0,5247
		$Fth^{fl/fl} \rightarrow Fth^{fl/fl}$	vs.	$Fth^{LysM\Delta/\Delta} \rightarrow Fth^{R26\Delta/\Delta}$	WAT (Late onset)	NS	0,6627
		$Fth^{fl/fl} \rightarrow Fth^{R26\Delta/\Delta}$	vs.	$Fth^{LysM\Delta/\Delta} \rightarrow Fth^{R26\Delta/\Delta}$	WAT (Late onset)	NS	0,5468
		$Fth^{fl/fl} \rightarrow Fth^{fl/fl}$	vs.	$Fth^{fl/fl} \rightarrow Fth^{R26\Delta/\Delta}$	WAT (Late onset)	NS	0,9545
		$Fth^{fl/fl} \rightarrow Fth^{fl/fl}$	vs.	$Fth^{LysM\Delta/\Delta} \rightarrow Fth^{R26\Delta/\Delta}$	Heart (Late onset)	NS	0,9927
		$Fth^{fl/fl} \rightarrow Fth^{R26\Delta/\Delta}$	vs.	$Fth^{LysM\Delta/\Delta} \rightarrow Fth^{R26\Delta/\Delta}$	Heart (Late onset)	NS	0,7918
		$Fth^{fl/fl} \rightarrow Fth^{fl/fl}$	vs.	$Fth^{fl/fl} \rightarrow Fth^{R26\Delta/\Delta}$	Heart (Late onset)	NS	0,6882
	F	$Fth^{fl/fl} \rightarrow Fth^{fl/fl}$	vs.	$Fth^{R26\Delta/\Delta} \rightarrow Fth^{R26\Delta/\Delta}$	*MT-CO1*	*	0,0343
		$Fth^{fl/fl} \rightarrow Fth^{R26\Delta/\Delta}$	vs.	$Fth^{R26\Delta/\Delta} \rightarrow Fth^{R26\Delta/\Delta}$	*MT-CO1*	**	0,0016
		$Fth^{fl/fl} \rightarrow Fth^{fl/fl}$	vs.	$Fth^{fl/fl} \rightarrow Fth^{R26\Delta/\Delta}$	*MT-CO1*	NS	0,7116
		$Fth^{fl/fl} \rightarrow Fth^{fl/fl}$	vs.	$Fth^{R26\Delta/\Delta} \rightarrow Fth^{R26\Delta/\Delta}$	*MT-CyB*	*	0,0101
		$Fth^{fl/fl} \rightarrow Fth^{R26\Delta/\Delta}$	vs.	$Fth^{R26\Delta/\Delta} \rightarrow Fth^{R26\Delta/\Delta}$	*MT-CyB*	*	0,0159
		$Fth^{fl/fl} \rightarrow Fth^{fl/fl}$	vs.	$Fth^{fl/fl} \rightarrow Fth^{R26\Delta/\Delta}$	*MT-CyB*	NS	0,9541
		$Fth^{fl/fl} \rightarrow Fth^{fl/fl}$	vs.	$Fth^{R26\Delta/\Delta} \rightarrow Fth^{R26\Delta/\Delta}$	*PolG*	*	0,0487
		$Fth^{fl/fl} \rightarrow Fth^{R26\Delta/\Delta}$	vs.	$Fth^{R26\Delta/\Delta} \rightarrow Fth^{R26\Delta/\Delta}$	*PolG*	NS	0,6157
		$Fth^{fl/fl} \rightarrow Fth^{fl/fl}$	vs.	$Fth^{fl/fl} \rightarrow Fth^{R26\Delta/\Delta}$	*PolG*	NS	0,3515
		$Fth^{fl/fl} \rightarrow Fth^{fl/fl}$	vs.	$Fth^{R26\Delta/\Delta} \rightarrow Fth^{R26\Delta/\Delta}$	*Cs*	NS	0,1142
		$Fth^{fl/fl} \rightarrow Fth^{R26\Delta/\Delta}$	vs.	$Fth^{R26\Delta/\Delta} \rightarrow Fth^{R26\Delta/\Delta}$	*Cs*	**	0,0033
		$Fth^{fl/fl} \rightarrow Fth^{fl/fl}$	vs.	$Fth^{fl/fl} \rightarrow Fth^{R26\Delta/\Delta}$	*Cs*	NS	0,5382
		$Fth^{fl/fl} \rightarrow Fth^{fl/fl}$	vs.	$Fth^{R26\Delta/\Delta} \rightarrow Fth^{R26\Delta/\Delta}$	*Nrf1*	*	0,0178
		$Fth^{fl/fl} \rightarrow Fth^{R26\Delta/\Delta}$	vs.	$Fth^{R26\Delta/\Delta} \rightarrow Fth^{R26\Delta/\Delta}$	*Nrf1*	NS	0,1394
		$Fth^{fl/fl} \rightarrow Fth^{fl/fl}$	vs.	$Fth^{fl/fl} \rightarrow Fth^{R26\Delta/\Delta}$	*Nrf1*	NS	0,6439
		$Fth^{fl/fl} \rightarrow Fth^{fl/fl}$	vs.	$Fth^{LysM\Delta/\Delta} \rightarrow Fth^{R26\Delta/\Delta}$	*MT-CO1*	**	0,0059

	G	$Fth^{fl/fl}$ → $Fth^{R26\Delta/\Delta}$	vs.	$Fth^{LysM\Delta/\Delta}$ → $Fth^{R26\Delta/\Delta}$	*MT-CO1*	NS	0,1864
		$Fth^{fl/fl}$ → $Fth^{fl/fl}$	vs.	$Fth^{fl/fl}$ → $Fth^{R26\Delta/\Delta}$	*MT-CO1*	NS	0,2435
		$Fth^{fl/fl}$ → $Fth^{fl/fl}$	vs.	$Fth^{LysM\Delta/\Delta}$ → $Fth^{R26\Delta/\Delta}$	*MT-CyB*	**	0,0017
		$Fth^{fl/fl}$ → $Fth^{R26\Delta/\Delta}$	vs.	$Fth^{LysM\Delta/\Delta}$ → $Fth^{R26\Delta/\Delta}$	*MT-CyB*	**	0,0036
		$Fth^{fl/fl}$ → $Fth^{fl/fl}$	vs.	$Fth^{fl/fl}$ → $Fth^{R26\Delta/\Delta}$	*MT-CyB*	NS	0,9798
		$Fth^{fl/fl}$ → $Fth^{fl/fl}$	vs.	$Fth^{LysM\Delta/\Delta}$ → $Fth^{R26\Delta/\Delta}$	*PolG*	**	0,0039
		$Fth^{fl/fl}$ → $Fth^{R26\Delta/\Delta}$	vs.	$Fth^{LysM\Delta/\Delta}$ → $Fth^{R26\Delta/\Delta}$	*PolG*	***	0,0004
		$Fth^{fl/fl}$ → $Fth^{fl/fl}$	vs.	$Fth^{fl/fl}$ → $Fth^{R26\Delta/\Delta}$	*PolG*	NS	0,5812
		$Fth^{fl/fl}$ → $Fth^{fl/fl}$	vs.	$Fth^{LysM\Delta/\Delta}$ → $Fth^{R26\Delta/\Delta}$	*Cs*	**	0,0088
		$Fth^{fl/fl}$ → $Fth^{R26\Delta/\Delta}$	vs.	$Fth^{LysM\Delta/\Delta}$ → $Fth^{R26\Delta/\Delta}$	*Cs*	*	0,0435
		$Fth^{fl/fl}$ → $Fth^{fl/fl}$	vs.	$Fth^{fl/fl}$ → $Fth^{R26\Delta/\Delta}$	*Cs*	NS	0,827
		$Fth^{fl/fl}$ → $Fth^{fl/fl}$	vs.	$Fth^{LysM\Delta/\Delta}$ → $Fth^{R26\Delta/\Delta}$	*Nrf1*	NS	0,3112
		$Fth^{fl/fl}$ → $Fth^{R26\Delta/\Delta}$	vs.	$Fth^{LysM\Delta/\Delta}$ → $Fth^{R26\Delta/\Delta}$	*Nrf1*	NS	0,2847
		$Fth^{fl/fl}$ → $Fth^{fl/fl}$	vs.	$Fth^{fl/fl}$ → $Fth^{R26\Delta/\Delta}$	*Nrf1*	NS	0,9865
	J	$Fth^{fl/fl}$ → $Fth^{fl/fl}$	vs.	$Fth^{R26\Delta/\Delta}$ → $Fth^{R26\Delta/\Delta}$	CI	NS	0,1952
		$Fth^{fl/fl}$ → $Fth^{R26\Delta/\Delta}$	vs.	$Fth^{R26\Delta/\Delta}$ → $Fth^{R26\Delta/\Delta}$	CI	*	0,0294
		$Fth^{fl/fl}$ → $Fth^{fl/fl}$	vs.	$Fth^{fl/fl}$ → $Fth^{R26\Delta/\Delta}$	CI	NS	>0,9999
		$Fth^{fl/fl}$ → $Fth^{fl/fl}$	vs.	$Fth^{R26\Delta/\Delta}$ → $Fth^{R26\Delta/\Delta}$	CII	*	0,0167
		$Fth^{fl/fl}$ → $Fth^{R26\Delta/\Delta}$	vs.	$Fth^{R26\Delta/\Delta}$ → $Fth^{R26\Delta/\Delta}$	CII	*	0,0166
		$Fth^{fl/fl}$ → $Fth^{fl/fl}$	vs.	$Fth^{fl/fl}$ → $Fth^{R26\Delta/\Delta}$	CII	NS	>0,9999
		$Fth^{fl/fl}$ → $Fth^{fl/fl}$	vs.	$Fth^{R26\Delta/\Delta}$ → $Fth^{R26\Delta/\Delta}$	CIV	*	0,0483
		$Fth^{fl/fl}$ → $Fth^{R26\Delta/\Delta}$	vs.	$Fth^{R26\Delta/\Delta}$ → $Fth^{R26\Delta/\Delta}$	CIV	NS	0,1021
		$Fth^{fl/fl}$ → $Fth^{fl/fl}$	vs.	$Fth^{fl/fl}$ → $Fth^{R26\Delta/\Delta}$	CIV	NS	>0,9999
		$Fth^{fl/fl}$ → $Fth^{fl/fl}$	vs.	$Fth^{LysM\Delta/\Delta}$ → $Fth^{R26\Delta/\Delta}$	CIV	**	0,0055
		$Fth^{fl/fl}$ → $Fth^{R26\Delta/\Delta}$	vs.	$Fth^{LysM\Delta/\Delta}$ → $Fth^{R26\Delta/\Delta}$	CIV	NS	0,394
		$Fth^{fl/fl}$ → $Fth^{fl/fl}$	vs.	$Fth^{fl/fl}$ → $Fth^{R26\Delta/\Delta}$	CIV	NS	0,5044

Figure	Panel	Comparison			Note	Stars	p-value
7	F	$Fth^{fl/fl}$ → $Fth^{R26\Delta/\Delta}$	vs.	$Fth^{LysM\Delta/\Delta}$ → $Fth^{R26\Delta/\Delta}$	Liver - $Ly6C^{hi}$ PhAM MFI	***	0,0005
		$Fth^{fl/fl}$ → $Fth^{R26\Delta/\Delta}$	vs.	$Fth^{LysM\Delta/\Delta}$ → $Fth^{R26\Delta/\Delta}$	Liver - $Ly6C^{hi}$ % $PhAM^{+}$	*	0,0223
	G	$Fth^{fl/fl}$ → $Fth^{R26\Delta/\Delta}$	vs.	$Fth^{LysM\Delta/\Delta}$ → $Fth^{R26\Delta/\Delta}$	WAT - $Ly6C^{hi}$ PhAM MFI	*	0,0326
		$Fth^{fl/fl}$ → $Fth^{R26\Delta/\Delta}$	vs.	$Fth^{LysM\Delta/\Delta}$ → $Fth^{R26\Delta/\Delta}$	WAT - $Ly6C^{hi}$ % $PhAM^{+}$	*	0,026

Figure	Panel	Comparison			Note	Stars	p-value
8	A	$Tfam^{fl/fl}$ → $Fth^{fl/fl}$	vs.	$Fth^{R26\Delta/\Delta}$ → $Fth^{R26\Delta/\Delta}$	Survival	*	0,0232
		$Tfam^{fl/fl}$ → $Fth^{R26\Delta/\Delta}$	vs.	$Fth^{R26\Delta/\Delta}$ → $Fth^{R26\Delta/\Delta}$	Survival	**	0,0036
		$Tfam^{LysM\Delta/\Delta}$ → $Fth^{R26\Delta/\Delta}$	vs.	$Fth^{R26\Delta/\Delta}$ → $Fth^{R26\Delta/\Delta}$	Survival	NS	0,0706
		$Tfam^{fl/fl}$ → $Fth^{fl/fl}$	vs.	$Tfam^{LysM\Delta/\Delta}$ → $Fth^{R26\Delta/\Delta}$	Survival	*	0,0326
		$Tfam^{fl/fl}$ → $Fth^{R26\Delta/\Delta}$	vs.	$Tfam^{LysM\Delta/\Delta}$ → $Fth^{R26\Delta/\Delta}$	Survival	**	0,0048
	B	$Uqcrq^{fl/fl}$ → $Fth^{fl/fl}$	vs.	$Fth^{R26\Delta/\Delta}$ → $Fth^{R26\Delta/\Delta}$	Survival	**	0,0039
		$Uqcrq^{fl/fl}$ → $Fth^{R26\Delta/\Delta}$	vs.	$Fth^{R26\Delta/\Delta}$ → $Fth^{R26\Delta/\Delta}$	Survival	**	0,0039
		$Uqcrq^{LysM\Delta/\Delta}$ → $Fth^{R26\Delta/\Delta}$	vs.	$Fth^{R26\Delta/\Delta}$ → $Fth^{R26\Delta/\Delta}$	Survival	**	0,0082
		$Uqcrq^{fl/fl}$ → $Fth^{fl/fl}$	vs.	$Uqcrq^{LysM\Delta/\Delta}$ → $Fth^{R26\Delta/\Delta}$	Survival	NS	>0,9999
		$Uqcrq^{fl/fl}$ → $Fth^{R26\Delta/\Delta}$	vs.	$Uqcrq^{LysM\Delta/\Delta}$ → $Fth^{R26\Delta/\Delta}$	Survival	NS	>0,9999
	E	$PhAM^{R26}$	vs.	$PhAM^{LysM}$ → tdT^{R26}	Liver - $CD45^{+}$ PhAM MFI	****	<0,0001
		$PhAM^{R26}$	vs.	$PhAM^{LysM}$ → $tdT^{R26}Fth^{R26\Delta/\Delta}$	Liver - $CD45^{+}$ PhAM MFI	****	<0,0001
		$PhAM^{R26}$	vs.	$PhAM^{LysM}$ $Fth^{LysM\Delta/\Delta}$ → tdT^{R26} $Fth^{R26\Delta/\Delta}$	Liver - $CD45^{+}$ PhAM MFI	****	<0,0001
	H	$Fth^{fl/fl}$ → $PhAM^{R26}$	vs.	$Fth^{fl/fl}$ → $PhAM^{R26}$ $Fth^{R26\Delta/\Delta}$	Liver - $Ly6C^{hi}$ PhAM MFI	NS	0,7701
		$Fth^{fl/fl}$ → $PhAM^{R26}$	vs.	$Fth^{LysM\Delta/\Delta}$ → $PhAM^{R26}$ $Fth^{R26\Delta/\Delta}$	Liver - $Ly6C^{hi}$ PhAM MFI	***	0,0002
		$Fth^{fl/fl}$ → $PhAM^{R26}$ $Fth^{R26\Delta/\Delta}$	vs.	$Fth^{LysM\Delta/\Delta}$ → $PhAM^{R26}$ $Fth^{R26\Delta/\Delta}$	Liver - $Ly6C^{hi}$ PhAM MFI	***	0,0005
	K	$Fth^{fl/fl}$ + Vehicle	vs.	$Fth^{R26\Delta/\Delta}$ + Mitochondria	BMDM viability	**	0,0076
		$Fth^{R26\Delta/\Delta}$ + Vehicle	vs.	$Fth^{R26\Delta/\Delta}$ + Mitochondria	BMDM viability	**	0,0077
		$Fth^{fl/fl}$ + Mitochondria	vs.	$Fth^{R26\Delta/\Delta}$ + Mitochondria	BMDM viability	**	0,0082
	L	$Fth^{fl/fl}$ + Vehicle	vs.	$Fth^{R26\Delta/\Delta}$ + Iron	BMDM viability	****	<0,0001
		$Fth^{R26\Delta/\Delta}$ + Vehicle	vs.	$Fth^{R26\Delta/\Delta}$ + Iron	BMDM viability	****	<0,0001
		$Fth^{fl/fl}$ + Iron	vs.	$Fth^{R26\Delta/\Delta}$ + Iron	BMDM viability	****	<0,0001

Appendix Table S1: Table containing the p-values and significance levels for the different statistical pairwise comparisons from Figures 1 to 8. Statistical analysis details are described in the respective figure legend, and in the “Statistical analysis” paragraph within the Methods section.
