# Supplementary figures and images for: Homeostatic control of energy metabolism by monocyte-derived macrophages
Source: EMBO J. 2025 Nov 17;45(1):106–50. doi: 10.1038/s44318-025-00622-x (PMC12759084; doi:10.1038/s44318-025-00622-x)

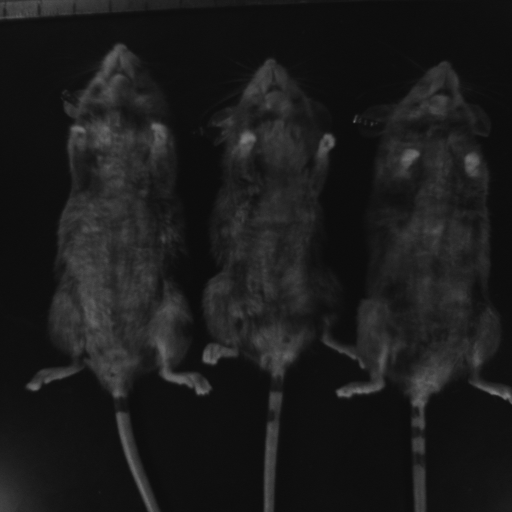

Supplement: Supplementary file 4 — Source data Fig. 2 [file 44318_2025_622_MOESM4_ESM.zip › Figure 2/Fig. 2G/FinalStack 1568 240sec.tif]

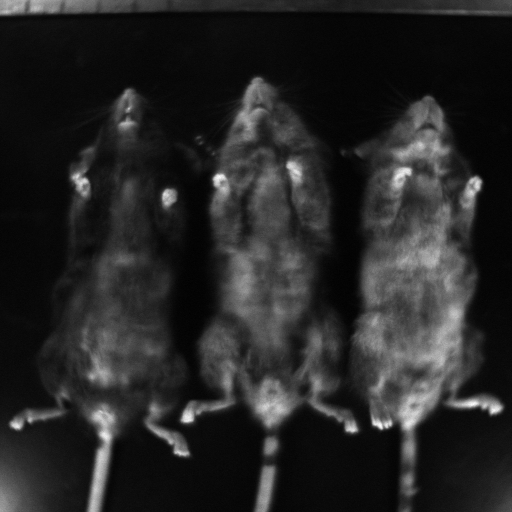

Supplement: Supplementary file 4 — Source data Fig. 2 [file 44318_2025_622_MOESM4_ESM.zip › Figure 2/Fig. 2G/FinalStack 163846 240sec.tif]

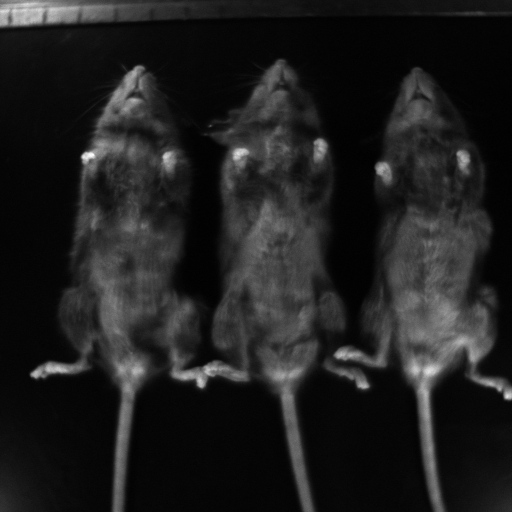

Supplement: Supplementary file 4 — Source data Fig. 2 [file 44318_2025_622_MOESM4_ESM.zip › Figure 2/Fig. 2H/FinalStack 15723_240sec.tif]

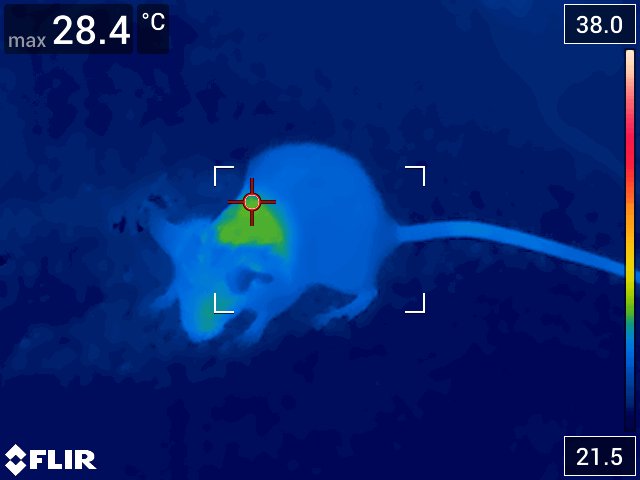

Supplement: Supplementary file 6 — Source data Fig. 4 [file 44318_2025_622_MOESM6_ESM.zip › Figure 4/Fig. 4A/IR_28-10-2019_0004.jpg]

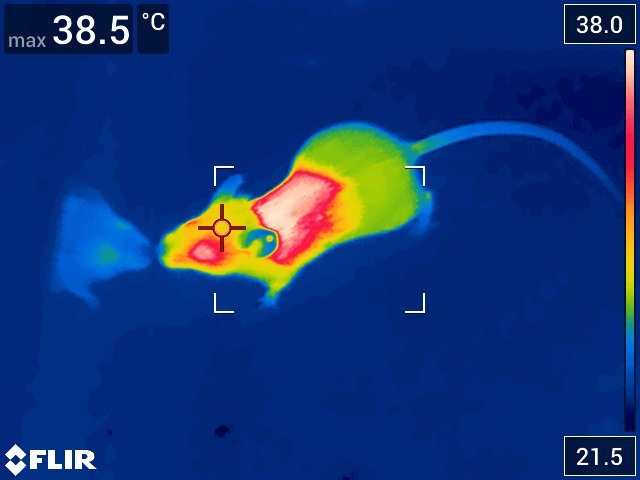

Supplement: Supplementary file 6 — Source data Fig. 4 [file 44318_2025_622_MOESM6_ESM.zip › Figure 4/Fig. 4A/IR_28-10-2019_0028.jpg]

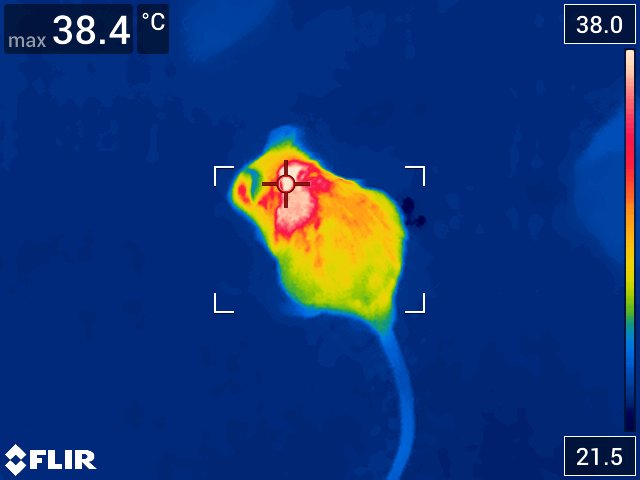

Supplement: Supplementary file 6 — Source data Fig. 4 [file 44318_2025_622_MOESM6_ESM.zip › Figure 4/Fig. 4A/IR_28-10-2019_0034.jpg]

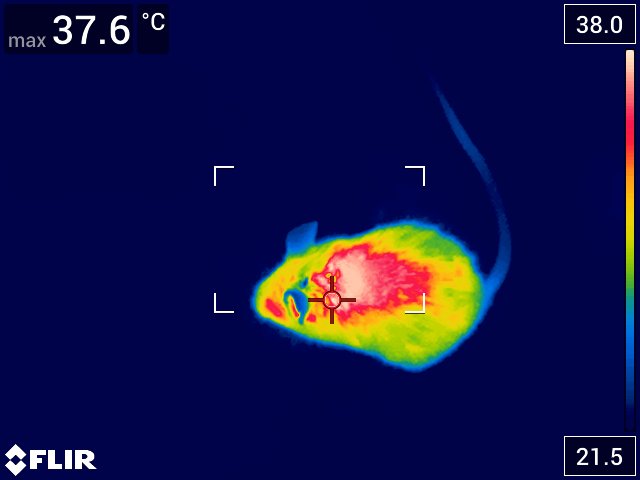

Supplement: Supplementary file 6 — Source data Fig. 4 [file 44318_2025_622_MOESM6_ESM.zip › Figure 4/Fig. 4C/IR_12-11-2019_0046.jpg]

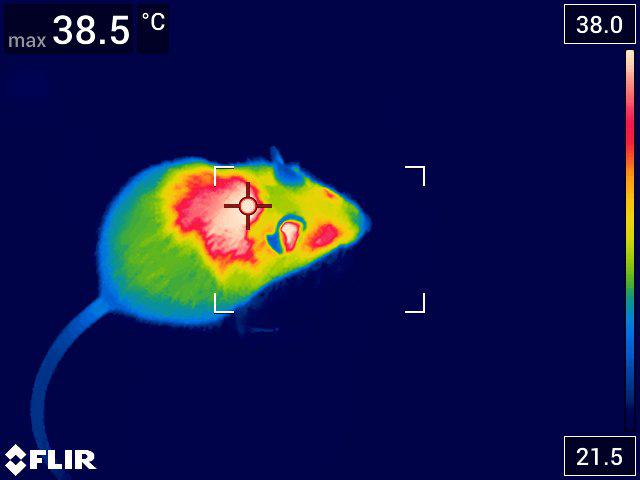

Supplement: Supplementary file 6 — Source data Fig. 4 [file 44318_2025_622_MOESM6_ESM.zip › Figure 4/Fig. 4C/IR_12-11-2019_0068.jpg]

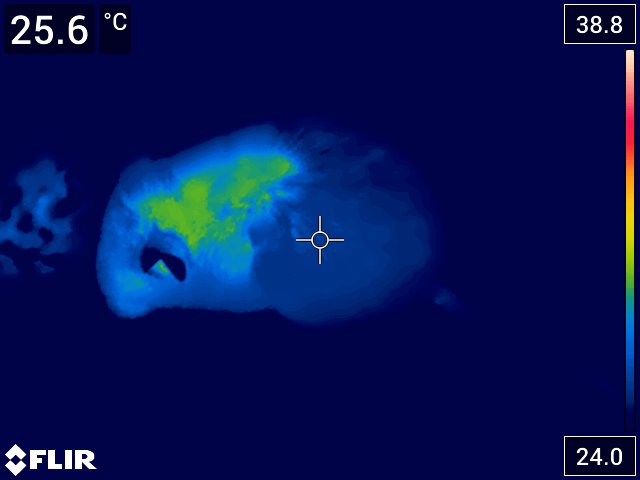

Supplement: Supplementary file 6 — Source data Fig. 4 [file 44318_2025_622_MOESM6_ESM.zip › Figure 4/Fig. 4C/IR_24_04_2021_0001.jpg]

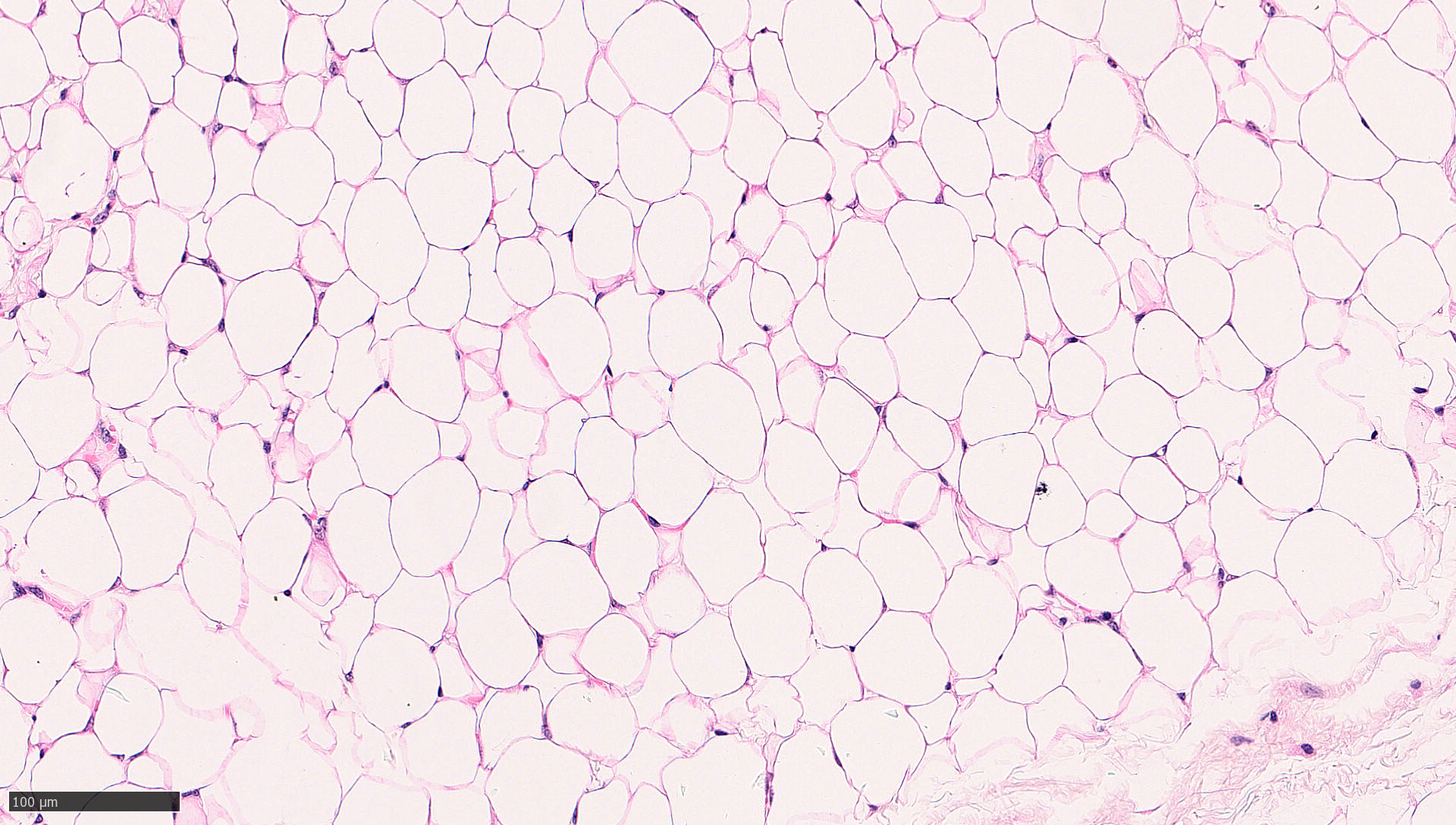

Supplement: Supplementary file 6 — Source data Fig. 4 [file 44318_2025_622_MOESM6_ESM.zip › Figure 4/Fig. 4E/FTH_FTH_20x.tif]

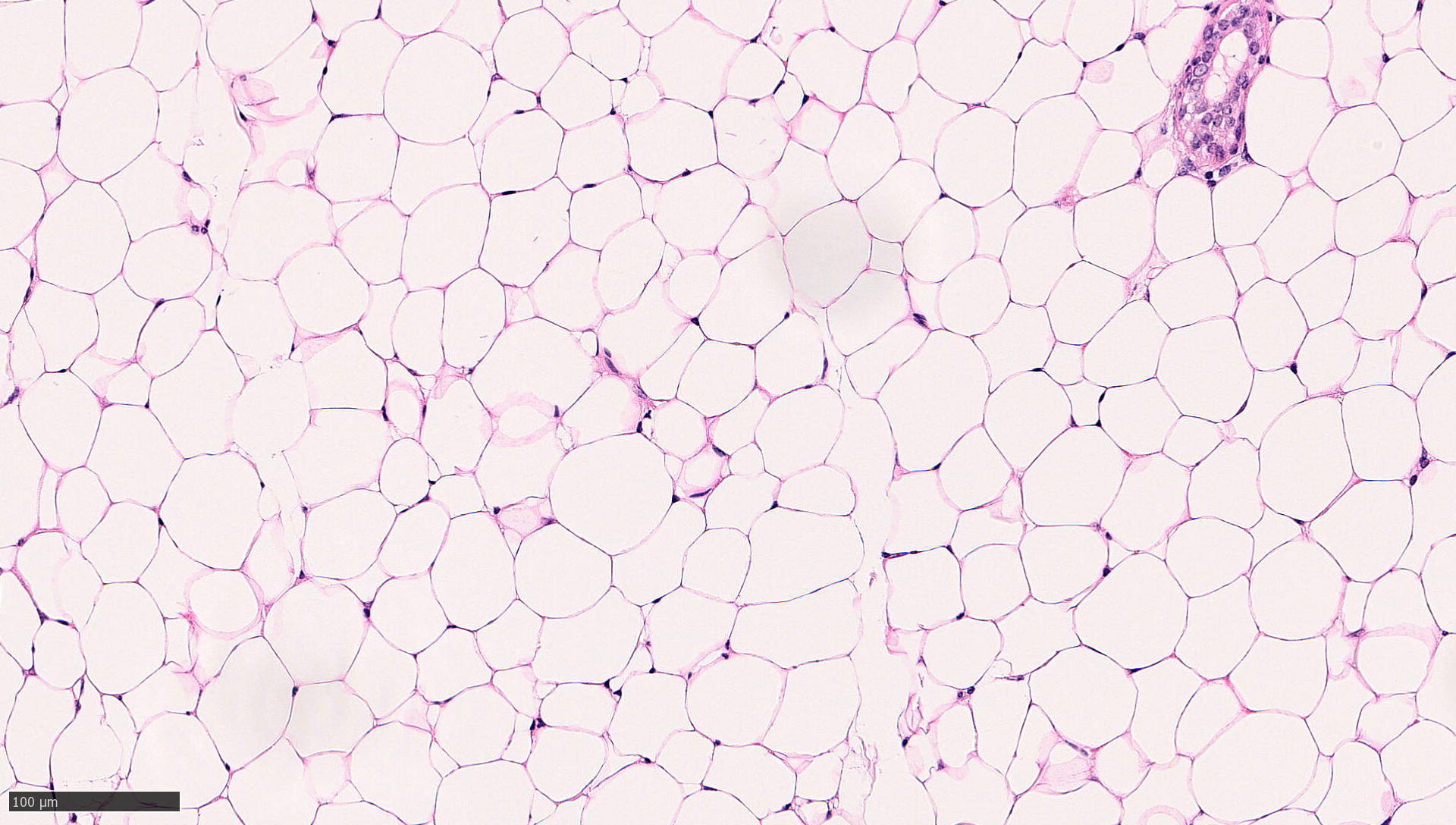

Supplement: Supplementary file 6 — Source data Fig. 4 [file 44318_2025_622_MOESM6_ESM.zip › Figure 4/Fig. 4E/FTH_R26CreFTH_20x.tif]

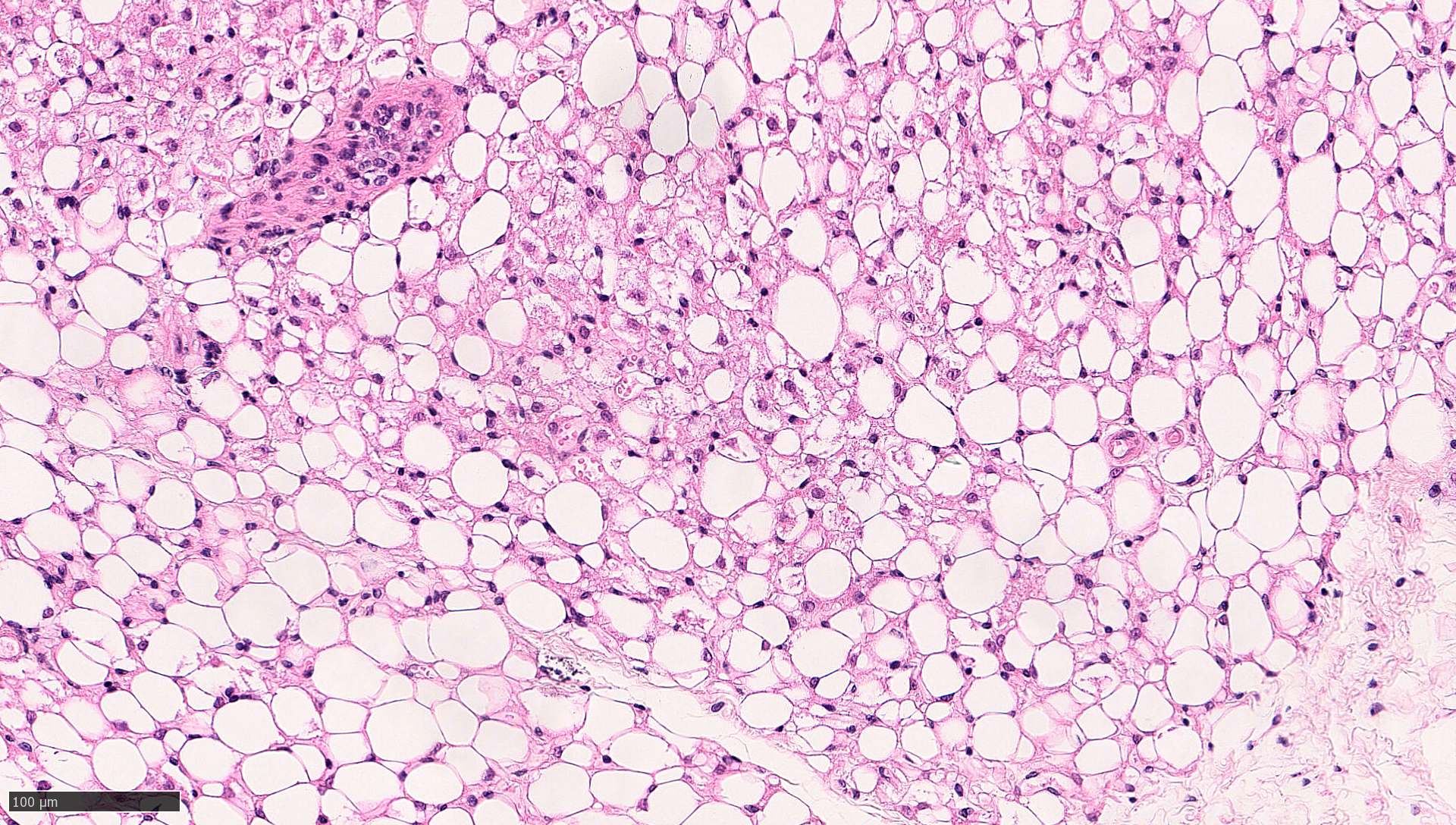

Supplement: Supplementary file 6 — Source data Fig. 4 [file 44318_2025_622_MOESM6_ESM.zip › Figure 4/Fig. 4E/R26CreFTH_R26CreFTH_20x.tif]

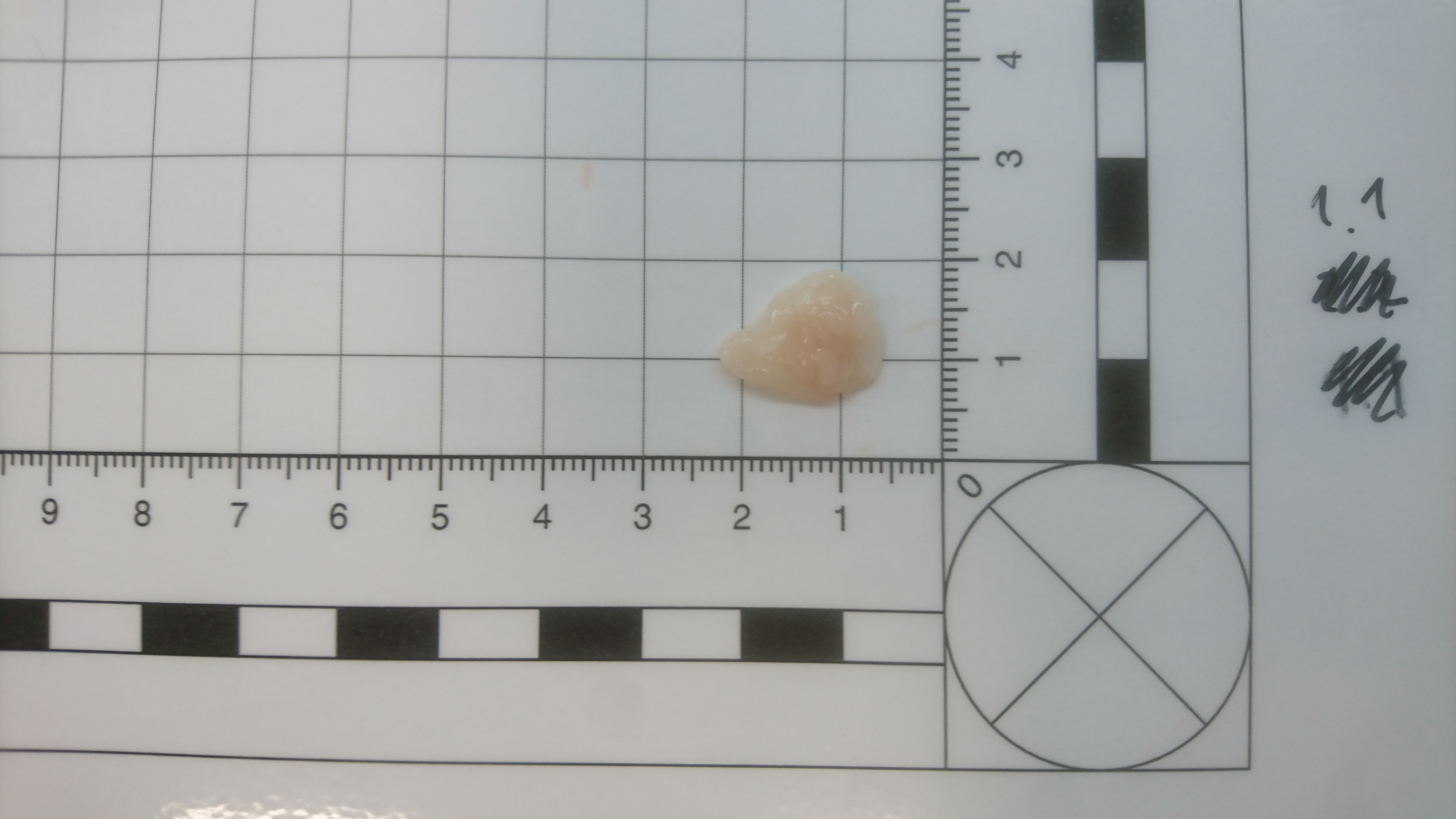

Supplement: Supplementary file 6 — Source data Fig. 4 [file 44318_2025_622_MOESM6_ESM.zip › Figure 4/Fig. 4E/RM098IV_eWAT_1.1_2.jpg]

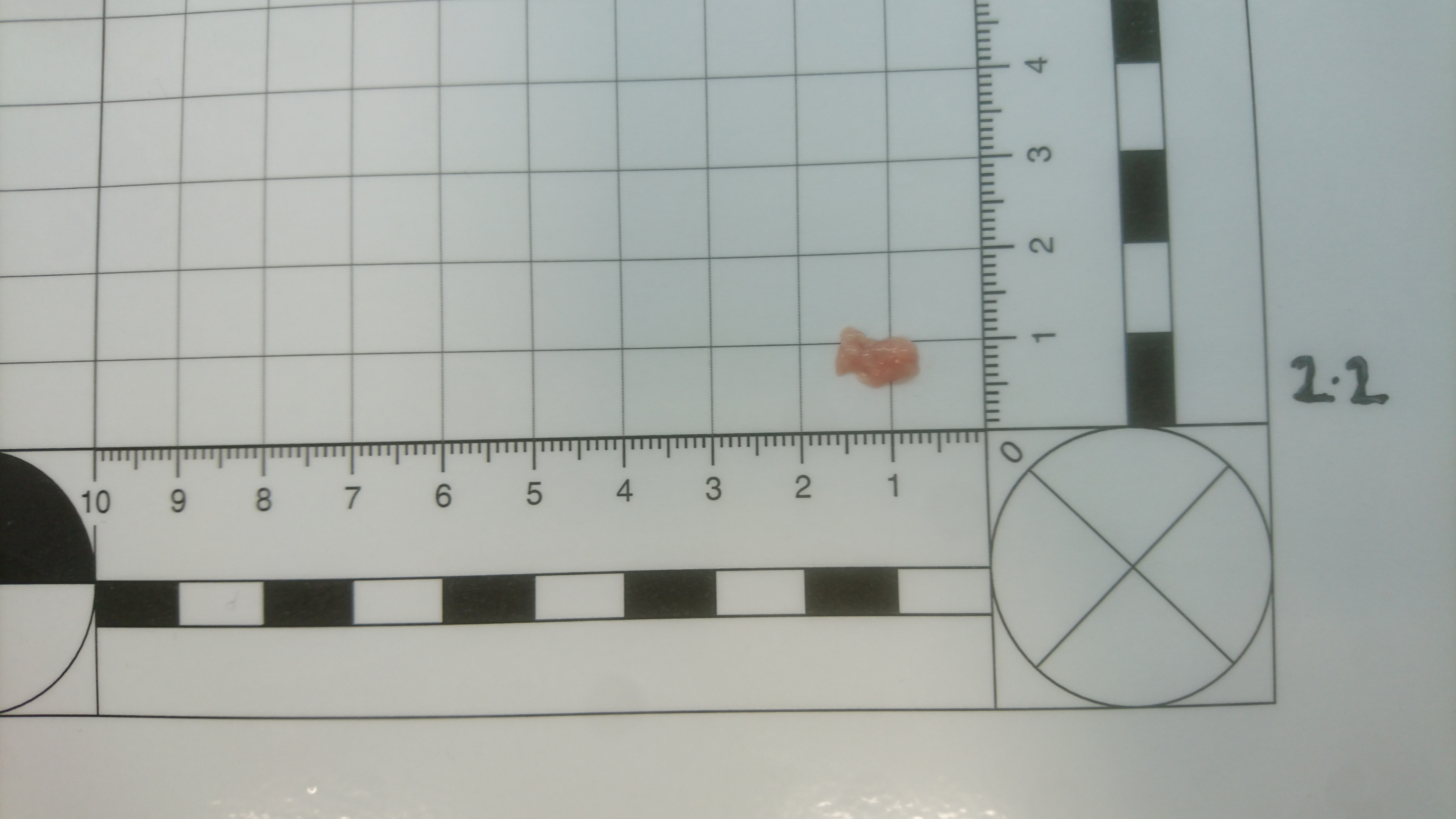

Supplement: Supplementary file 6 — Source data Fig. 4 [file 44318_2025_622_MOESM6_ESM.zip › Figure 4/Fig. 4E/RM098IV_eWAT_2.2_1.jpg]

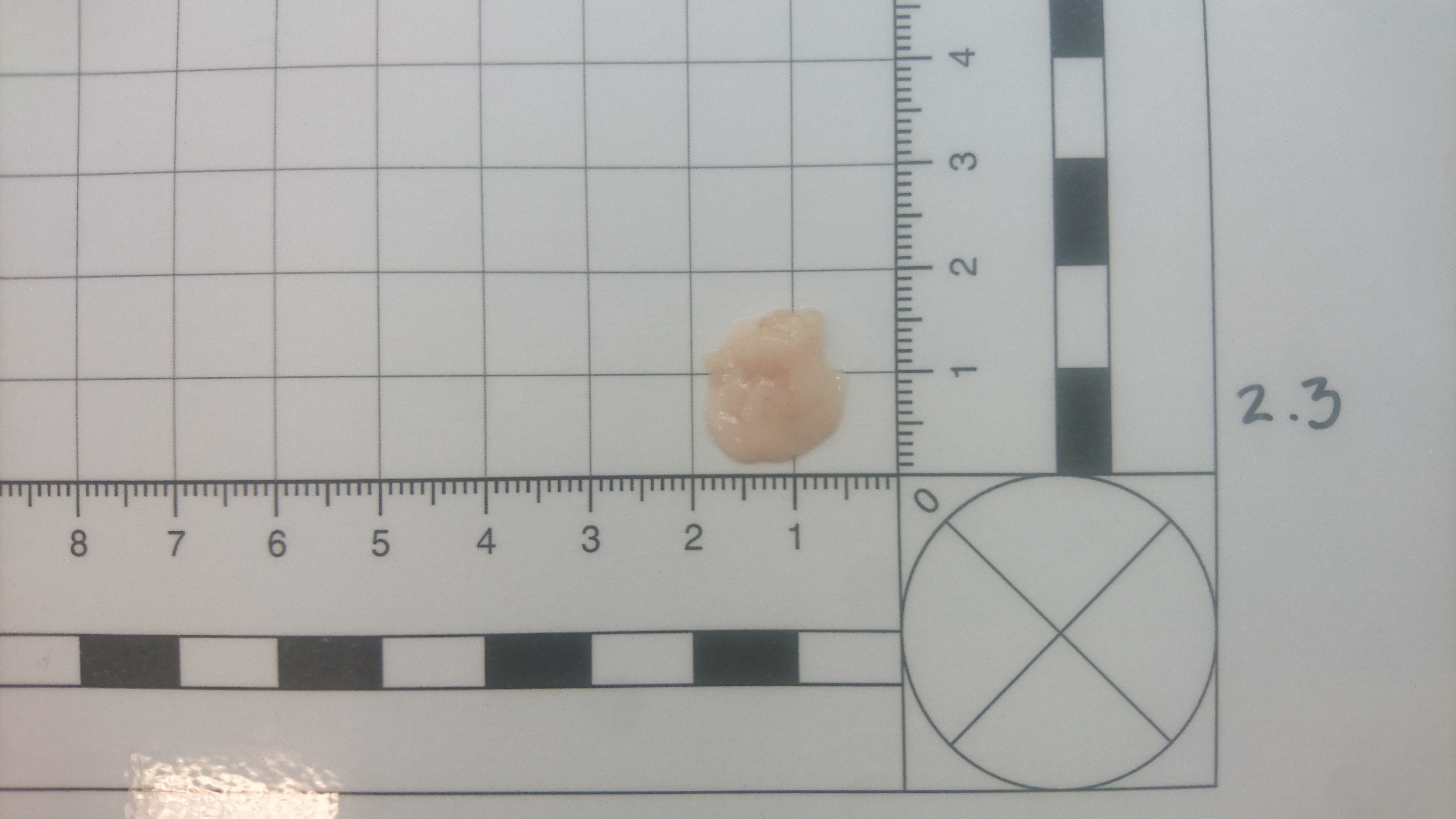

Supplement: Supplementary file 6 — Source data Fig. 4 [file 44318_2025_622_MOESM6_ESM.zip › Figure 4/Fig. 4E/RM098IV_eWAT_2.3_1.jpg]

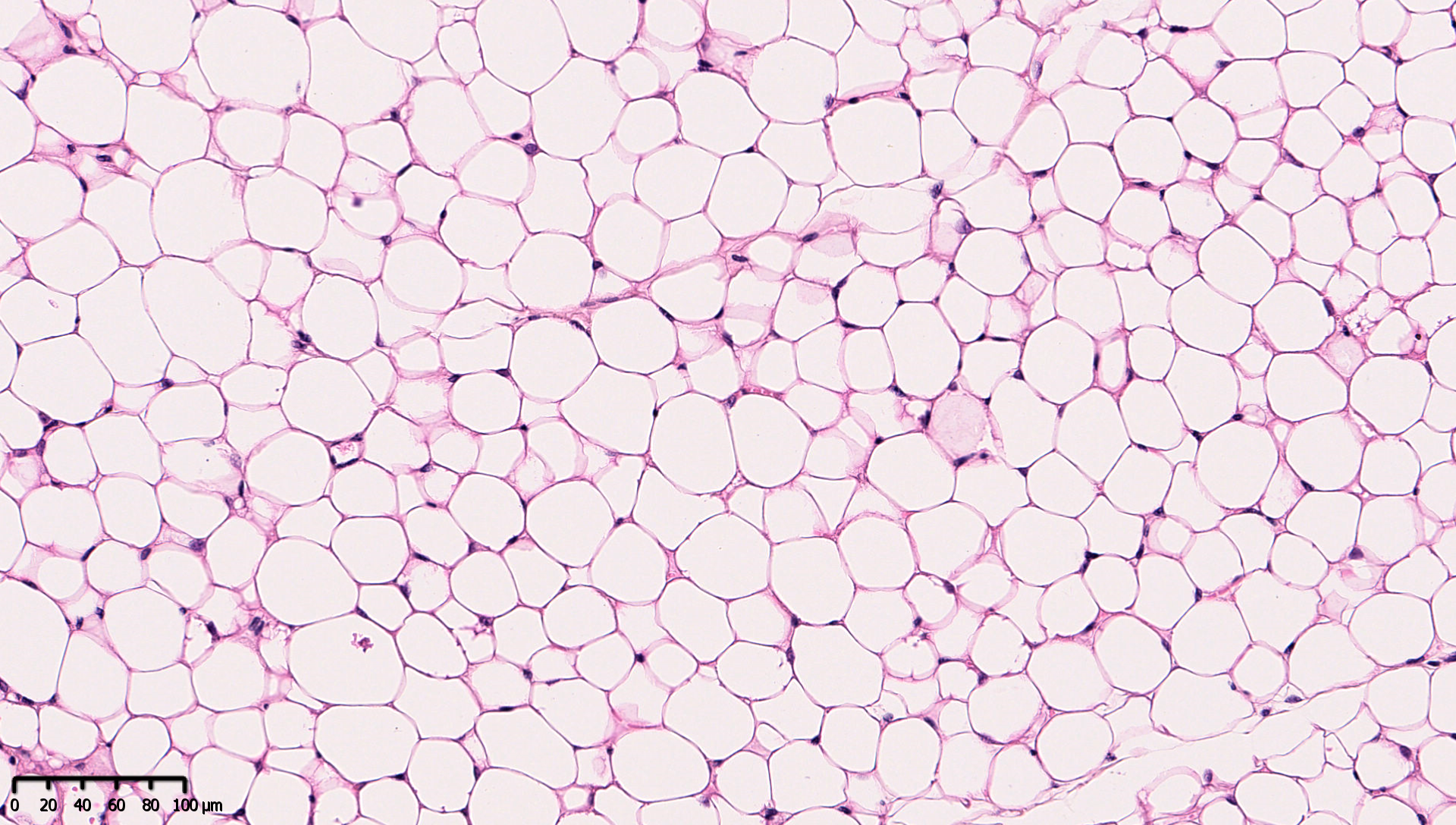

Supplement: Supplementary file 6 — Source data Fig. 4 [file 44318_2025_622_MOESM6_ESM.zip › Figure 4/Fig. 4G/FTH_FTH_20x.tif]

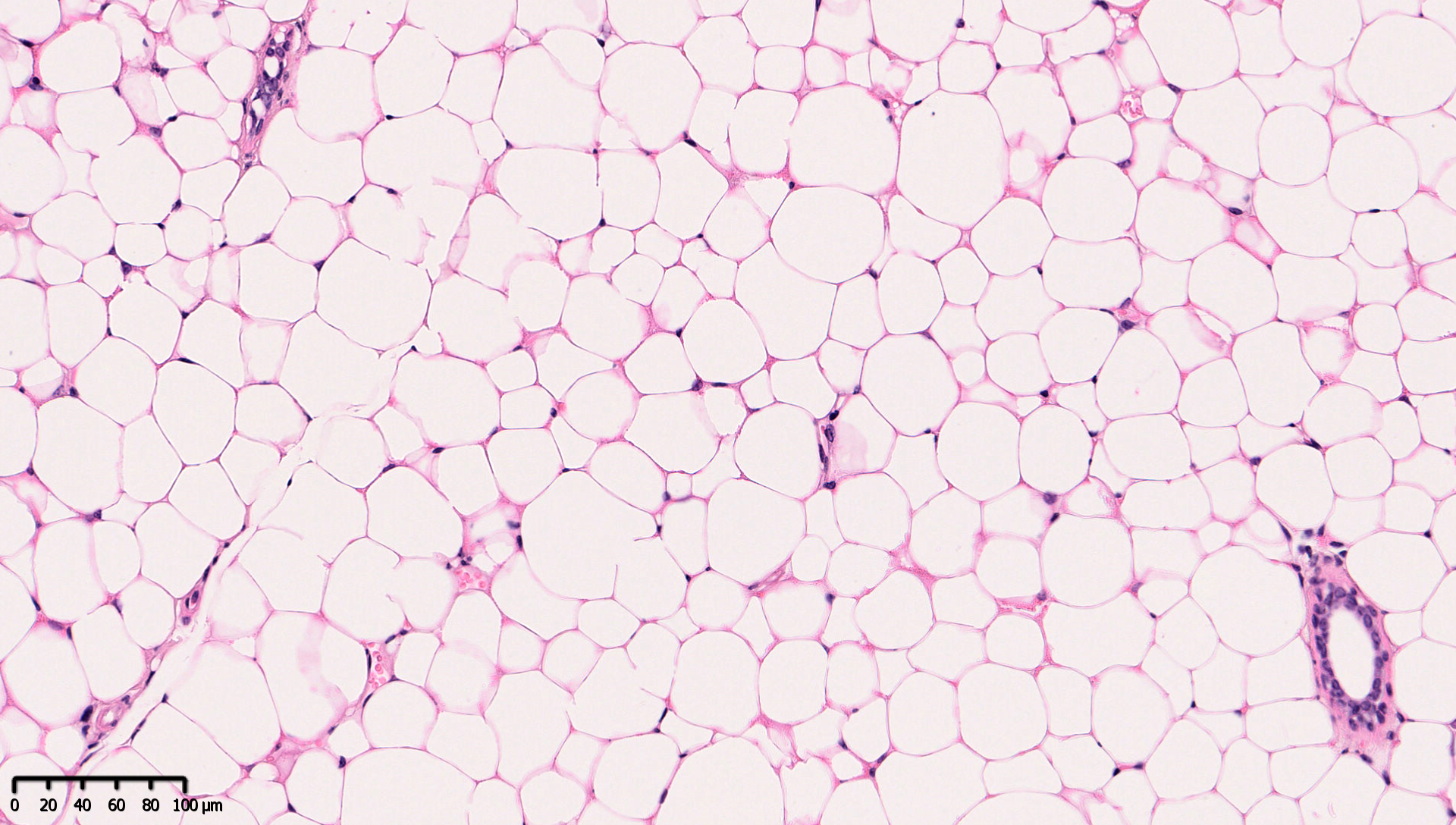

Supplement: Supplementary file 6 — Source data Fig. 4 [file 44318_2025_622_MOESM6_ESM.zip › Figure 4/Fig. 4G/FTH_R26CreFTH_20x.tif]

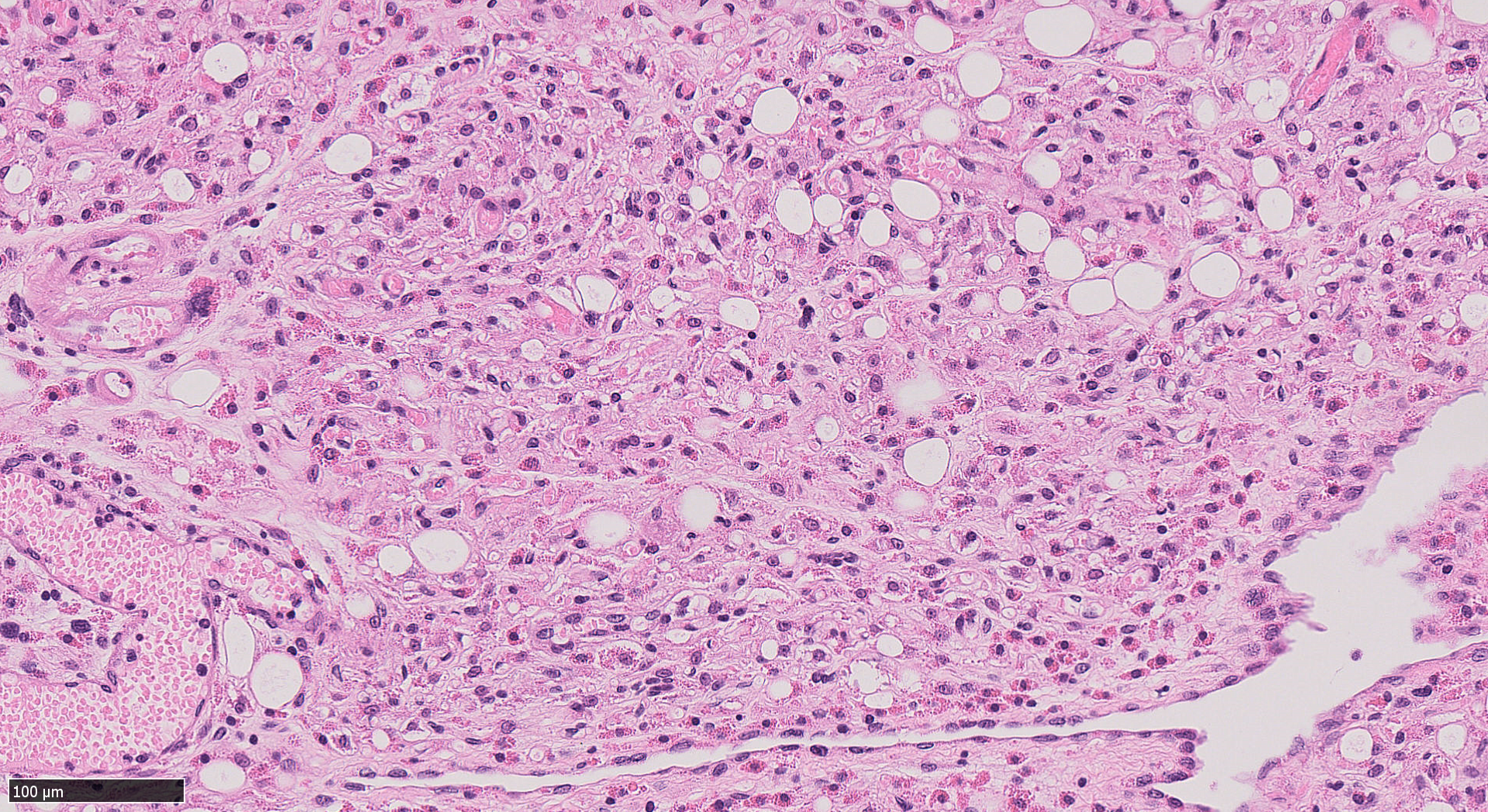

Supplement: Supplementary file 6 — Source data Fig. 4 [file 44318_2025_622_MOESM6_ESM.zip › Figure 4/Fig. 4G/LysMCreFTH_R26CreFTH_20x.tif]

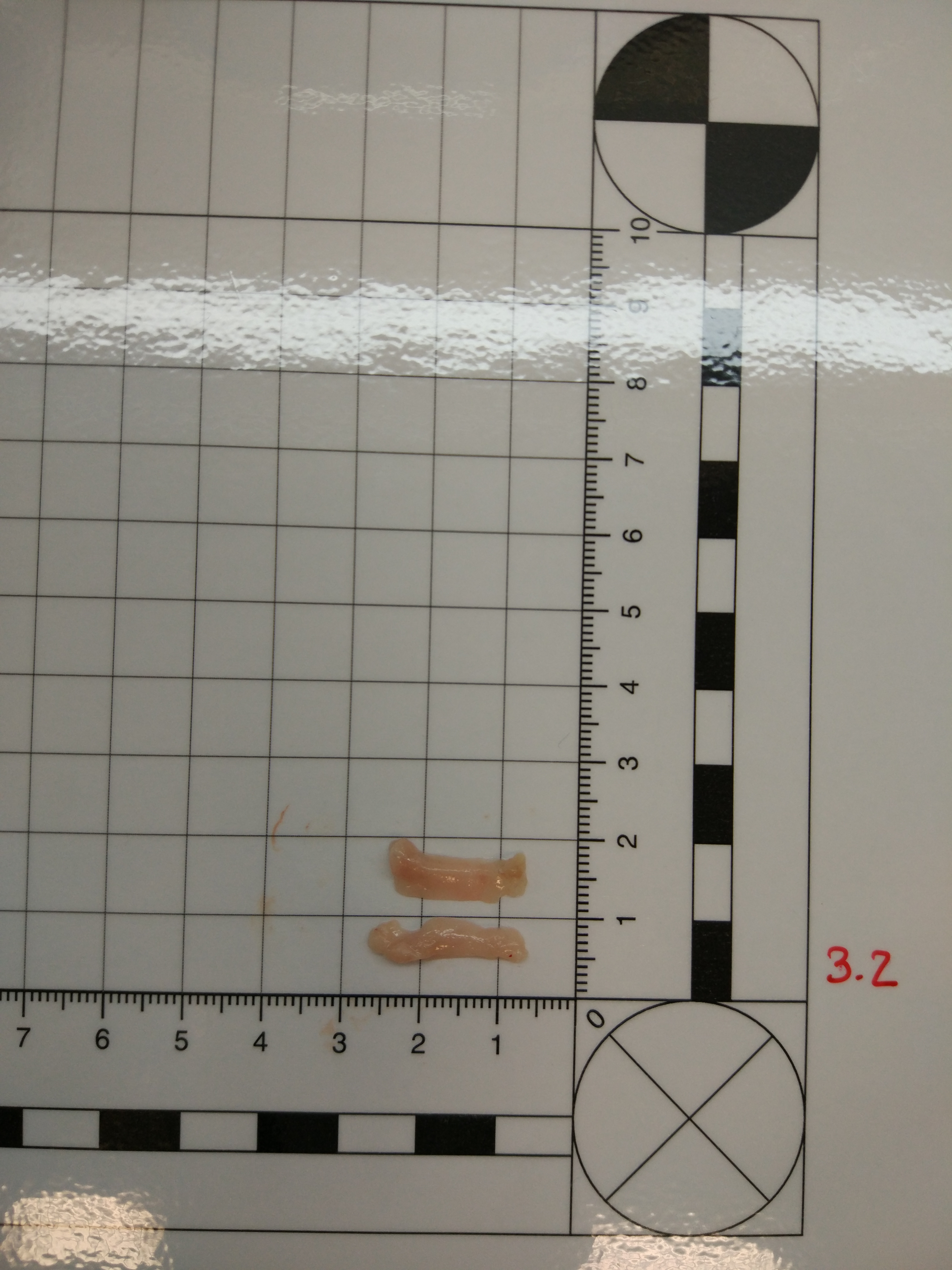

Supplement: Supplementary file 6 — Source data Fig. 4 [file 44318_2025_622_MOESM6_ESM.zip › Figure 4/Fig. 4G/RM098IV_WAT_3.2.jpg]

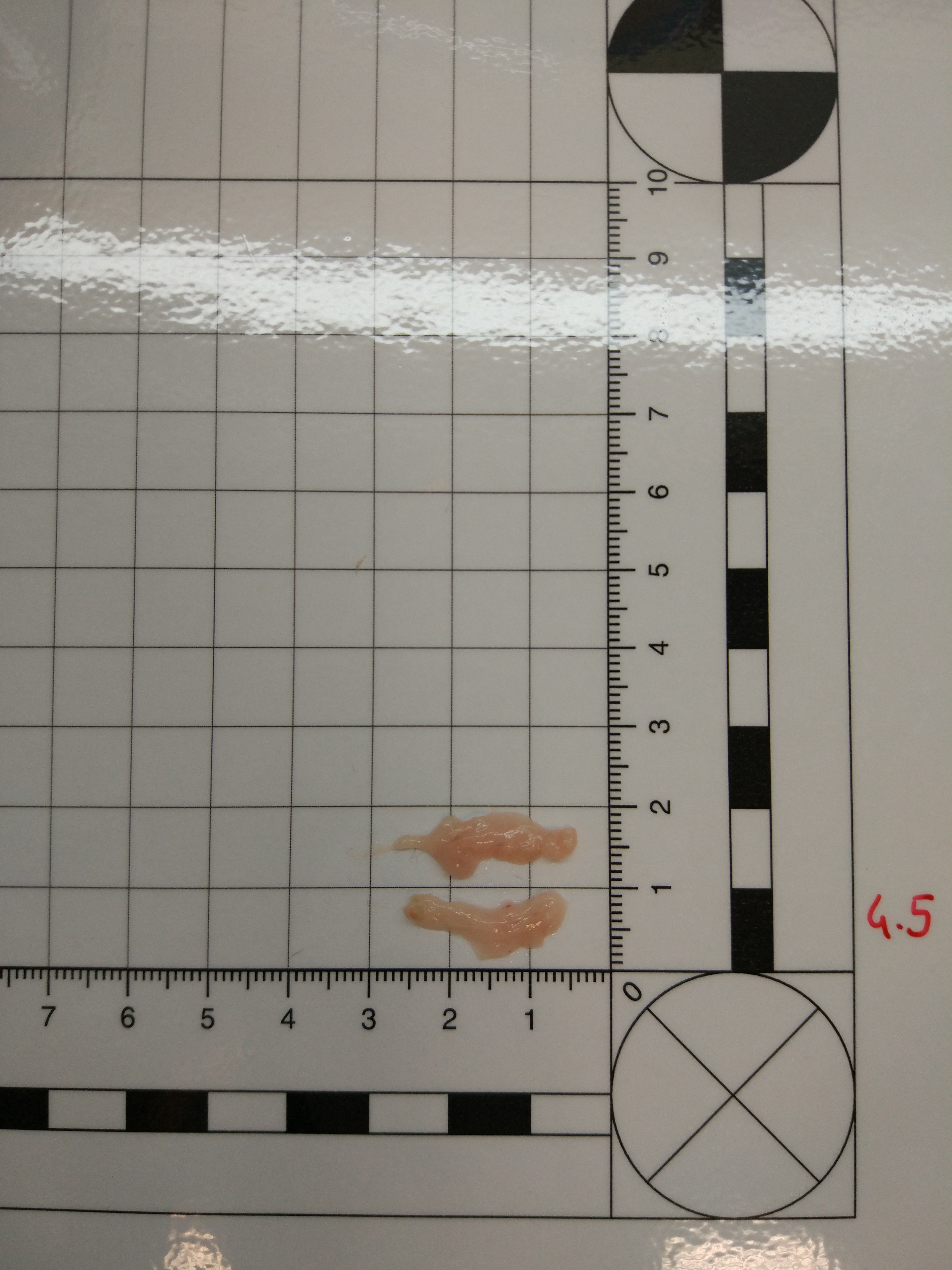

Supplement: Supplementary file 6 — Source data Fig. 4 [file 44318_2025_622_MOESM6_ESM.zip › Figure 4/Fig. 4G/RM098IV_WAT_4.5.jpg]

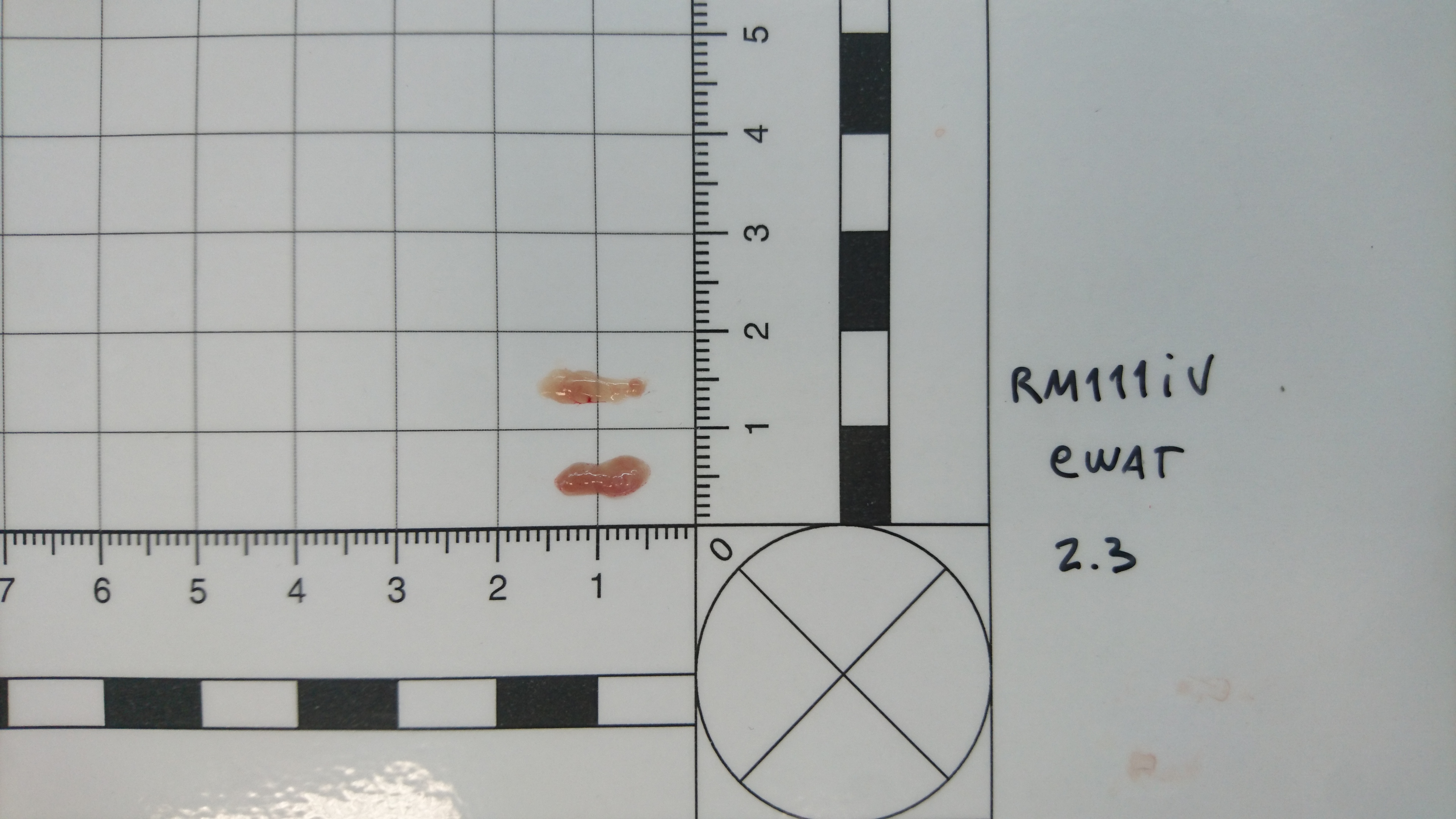

Supplement: Supplementary file 6 — Source data Fig. 4 [file 44318_2025_622_MOESM6_ESM.zip › Figure 4/Fig. 4G/RM111IV_WAT_2.3.jpg]

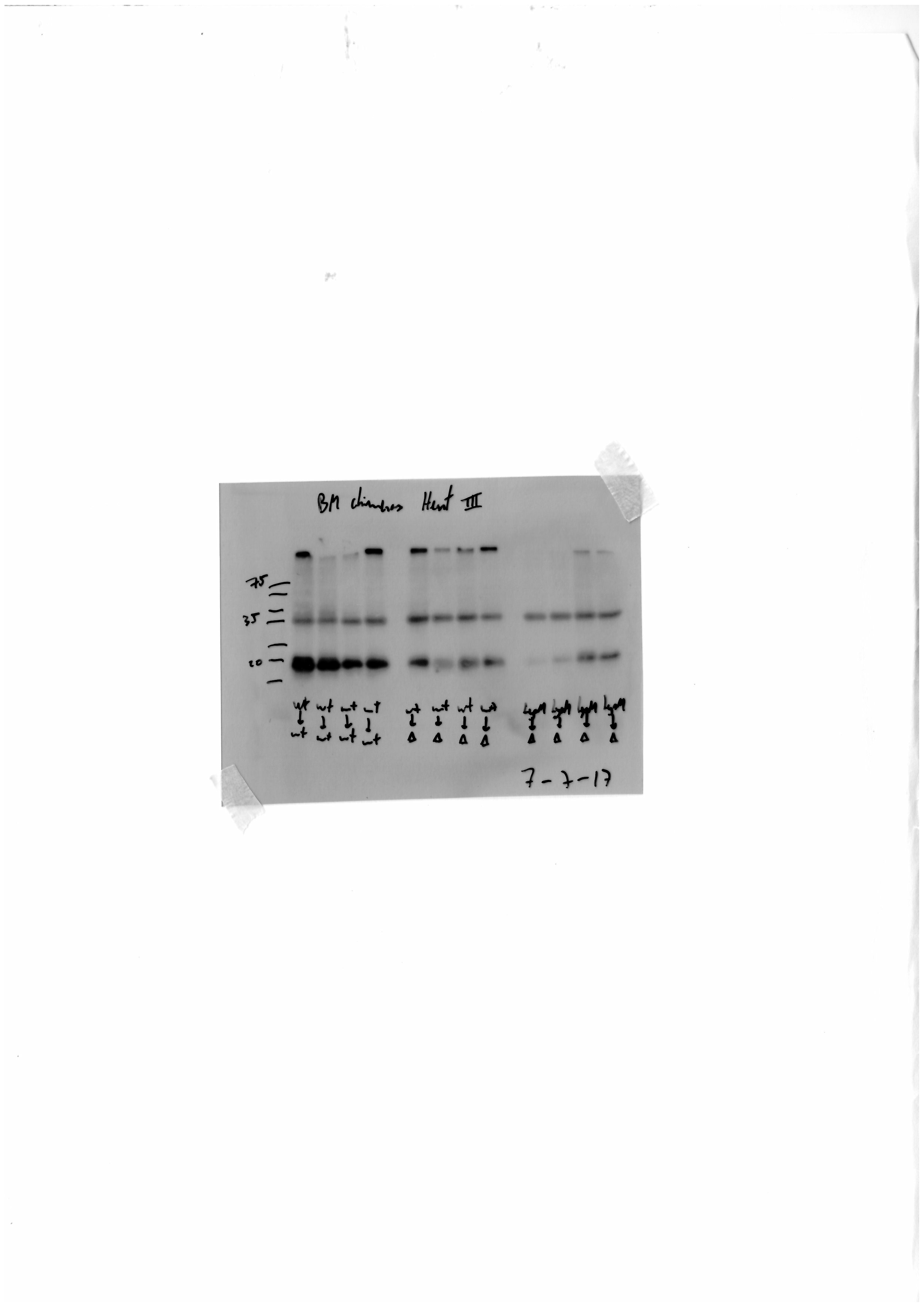

Supplement: Supplementary file 7 — Source data Fig. 5 [file 44318_2025_622_MOESM7_ESM.zip › Figure 5/Fig. 5A/Heart gapdh+fth.tif]

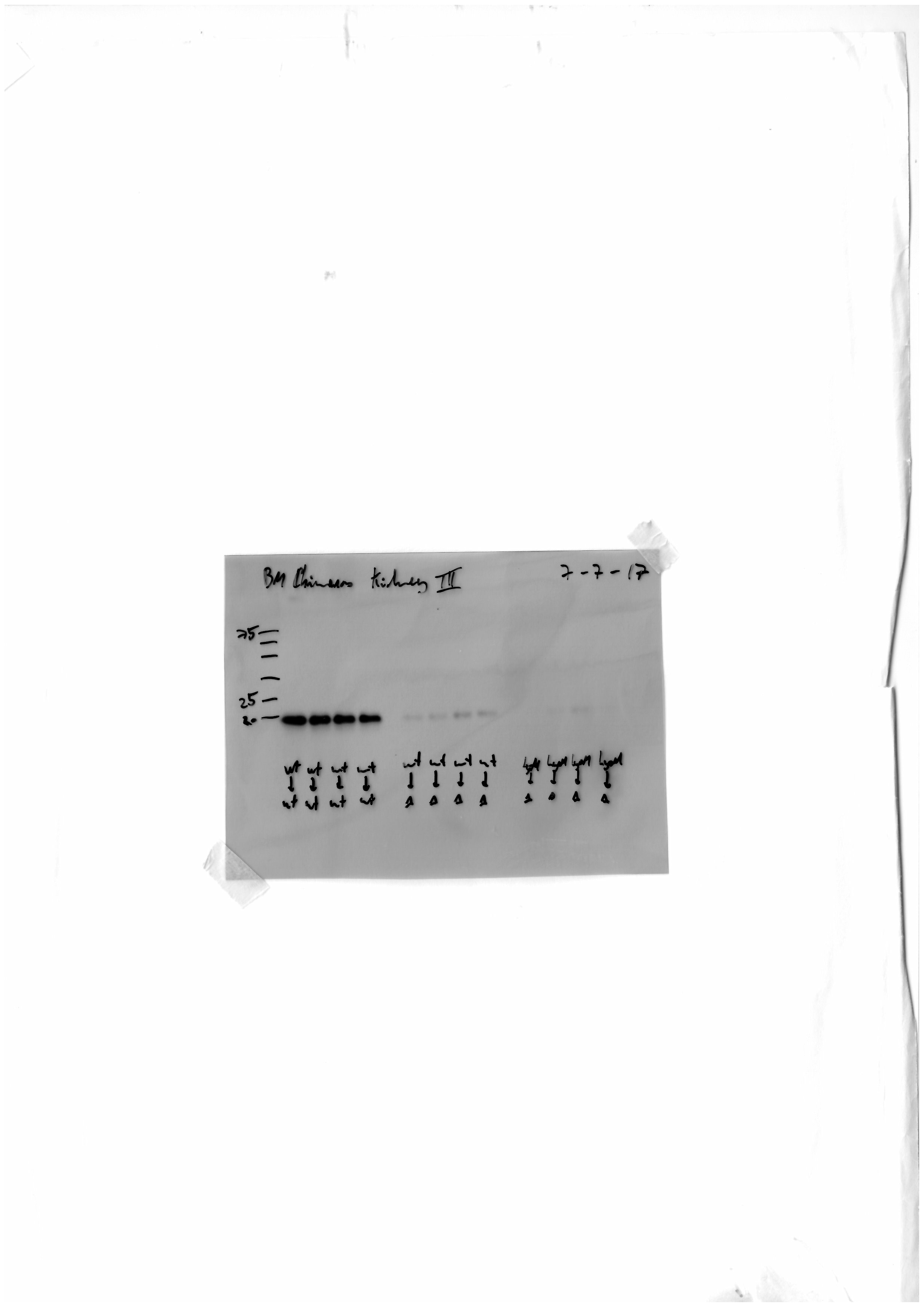

Supplement: Supplementary file 7 — Source data Fig. 5 [file 44318_2025_622_MOESM7_ESM.zip › Figure 5/Fig. 5A/kidney fth.tif]

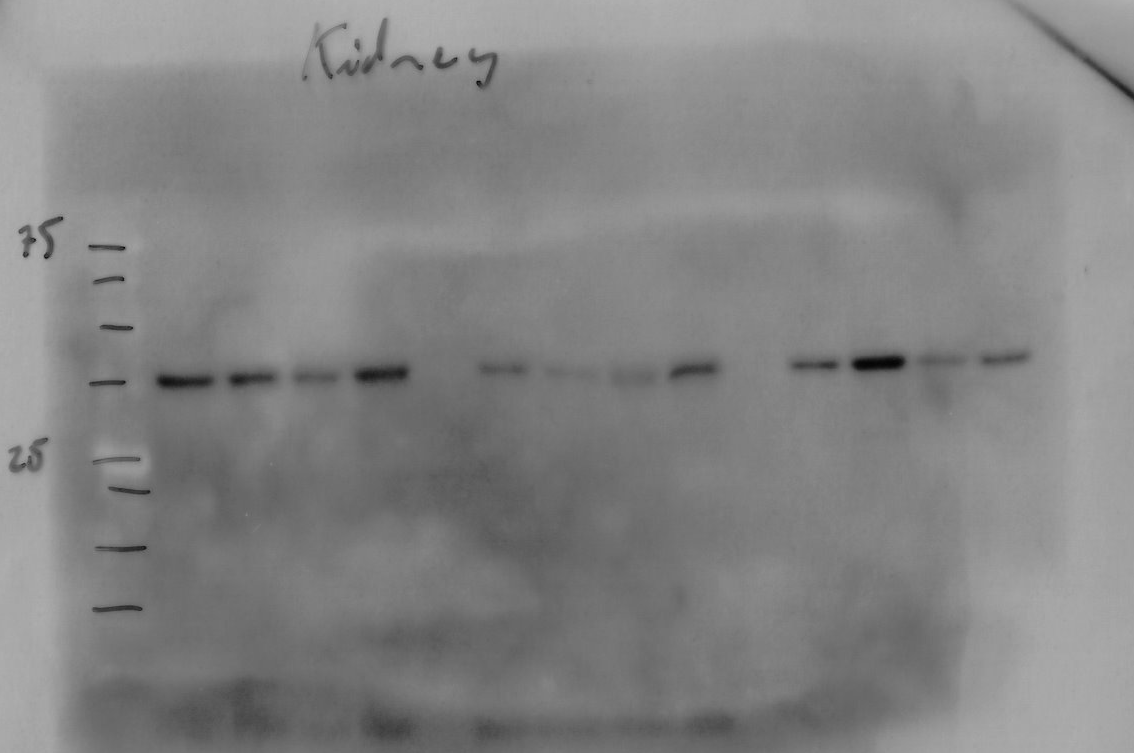

Supplement: Supplementary file 7 — Source data Fig. 5 [file 44318_2025_622_MOESM7_ESM.zip › Figure 5/Fig. 5A/Kidney gapdh.tif]

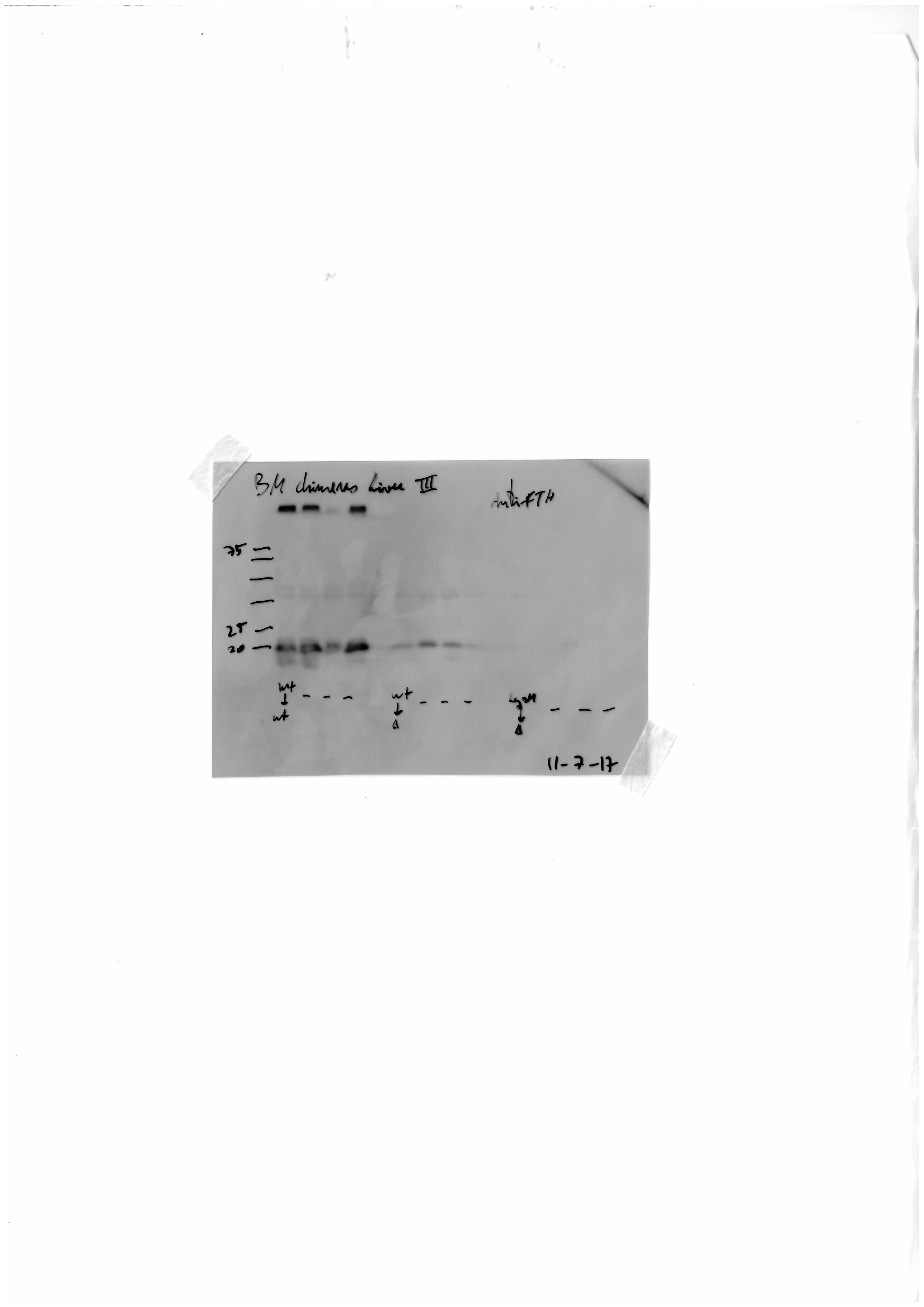

Supplement: Supplementary file 7 — Source data Fig. 5 [file 44318_2025_622_MOESM7_ESM.zip › Figure 5/Fig. 5A/Liver fth.tif]

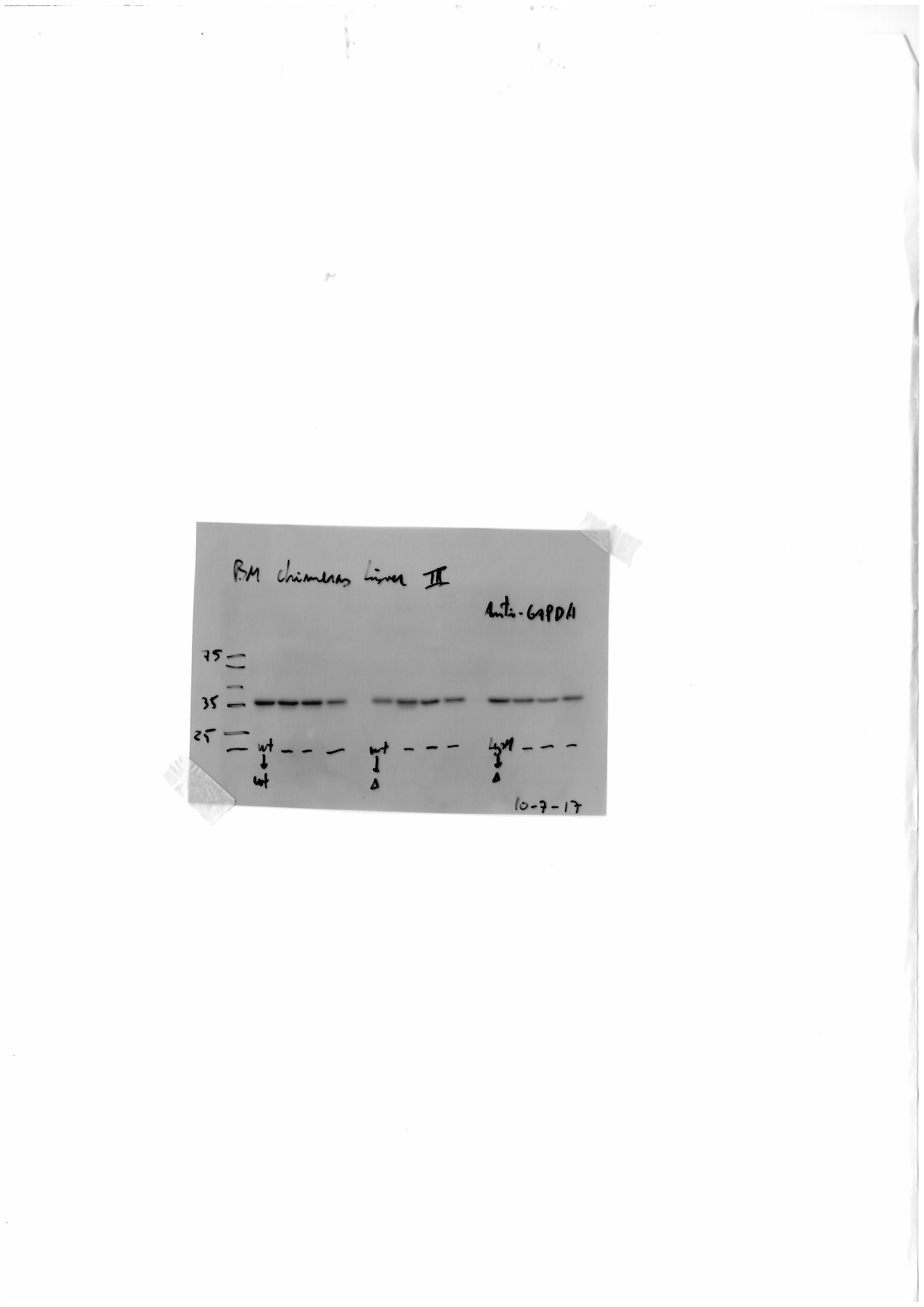

Supplement: Supplementary file 7 — Source data Fig. 5 [file 44318_2025_622_MOESM7_ESM.zip › Figure 5/Fig. 5A/Liver gapdh.tif]

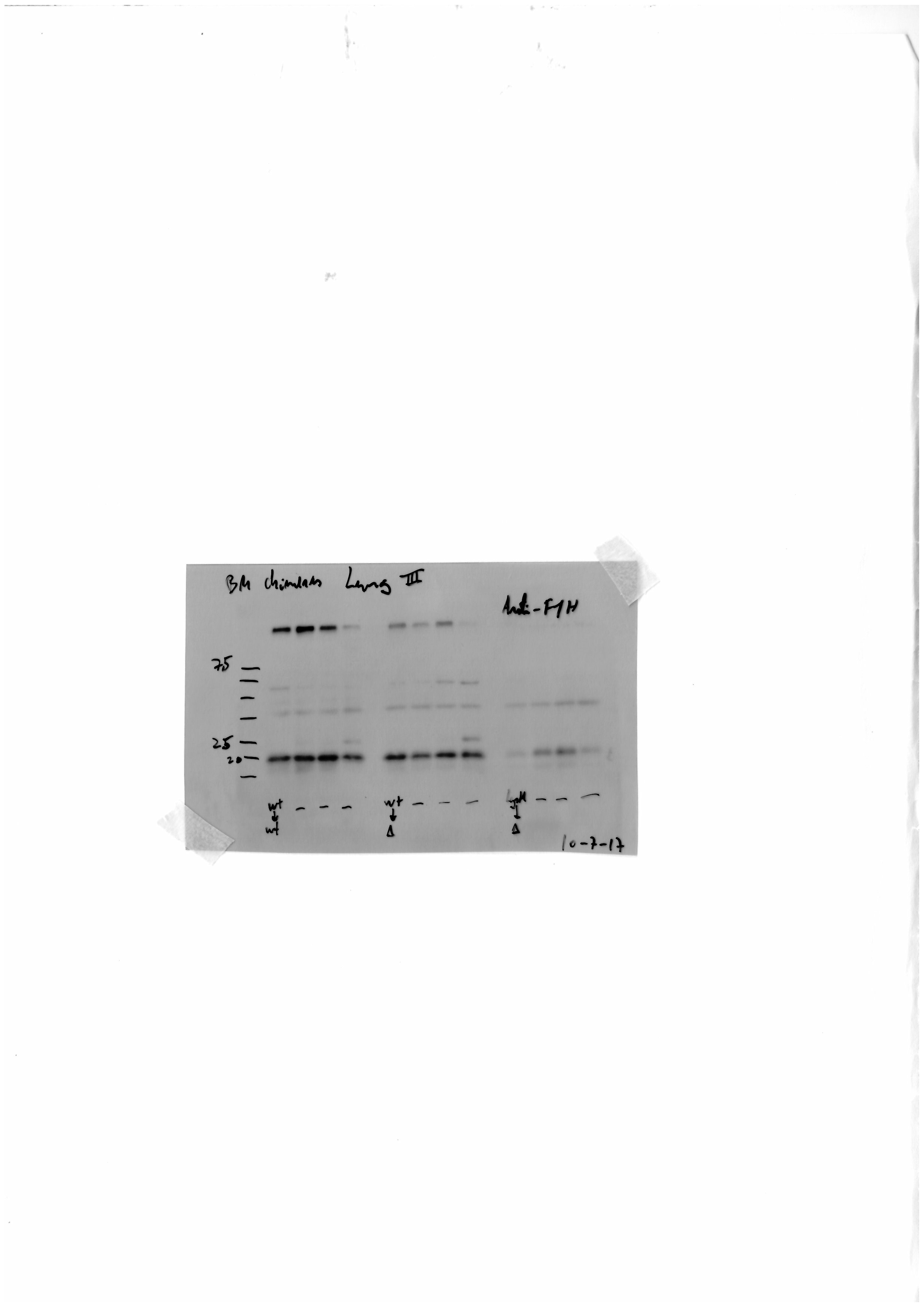

Supplement: Supplementary file 7 — Source data Fig. 5 [file 44318_2025_622_MOESM7_ESM.zip › Figure 5/Fig. 5A/Lung fth.tif]

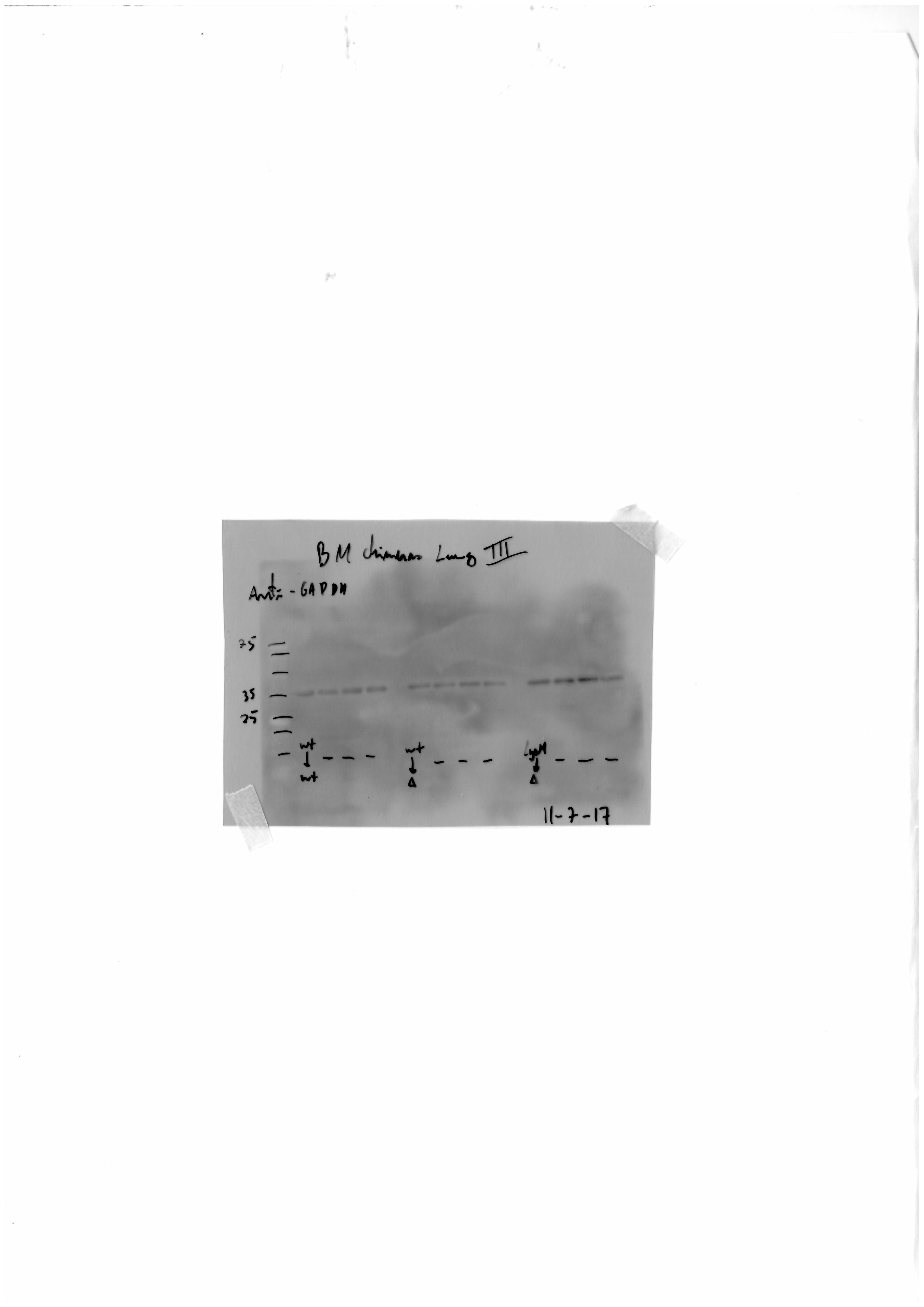

Supplement: Supplementary file 7 — Source data Fig. 5 [file 44318_2025_622_MOESM7_ESM.zip › Figure 5/Fig. 5A/Lung gapdh.tif]

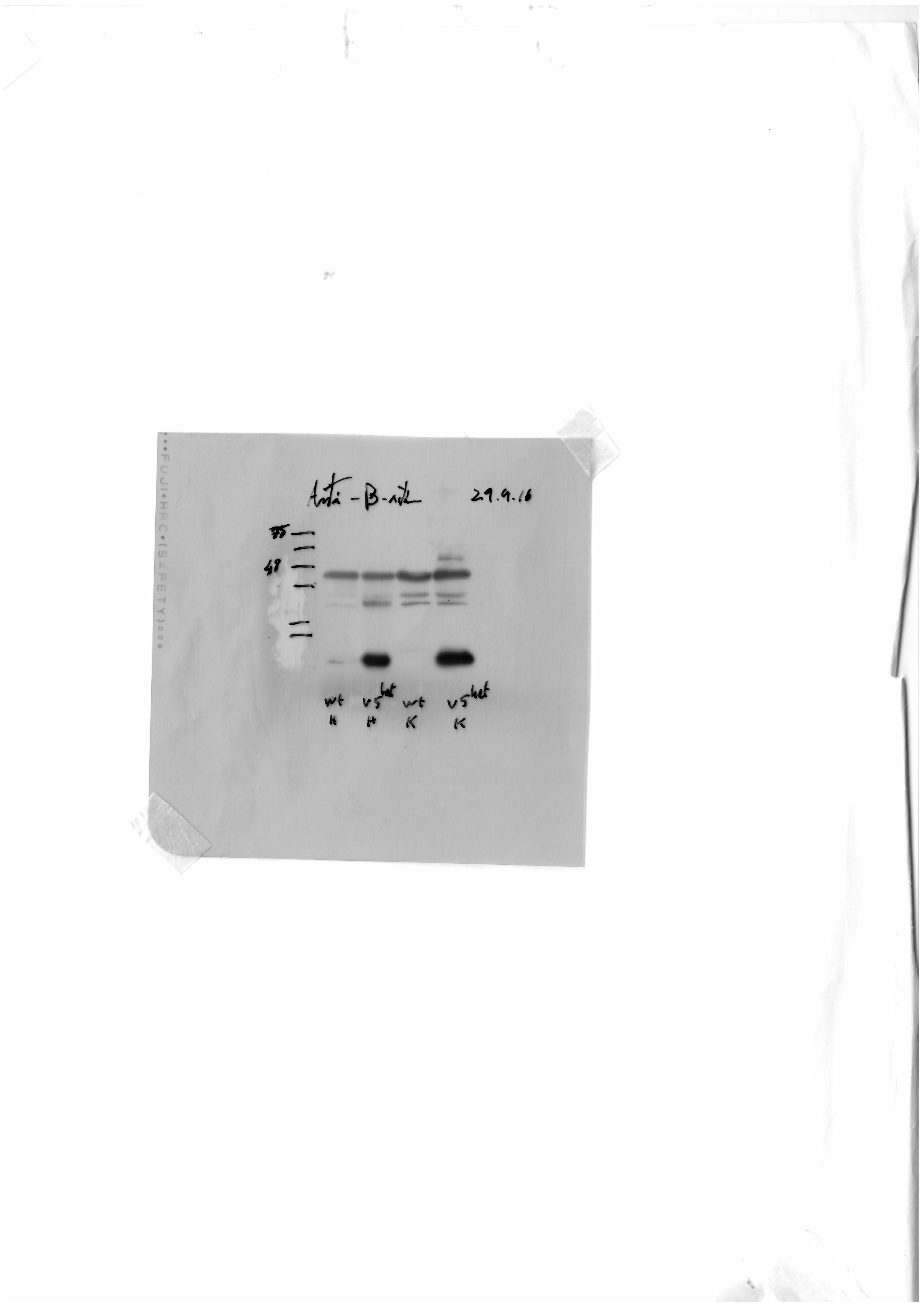

Supplement: Supplementary file 7 — Source data Fig. 5 [file 44318_2025_622_MOESM7_ESM.zip › Figure 5/Fig. 5D/b-actin wt v5.tif]

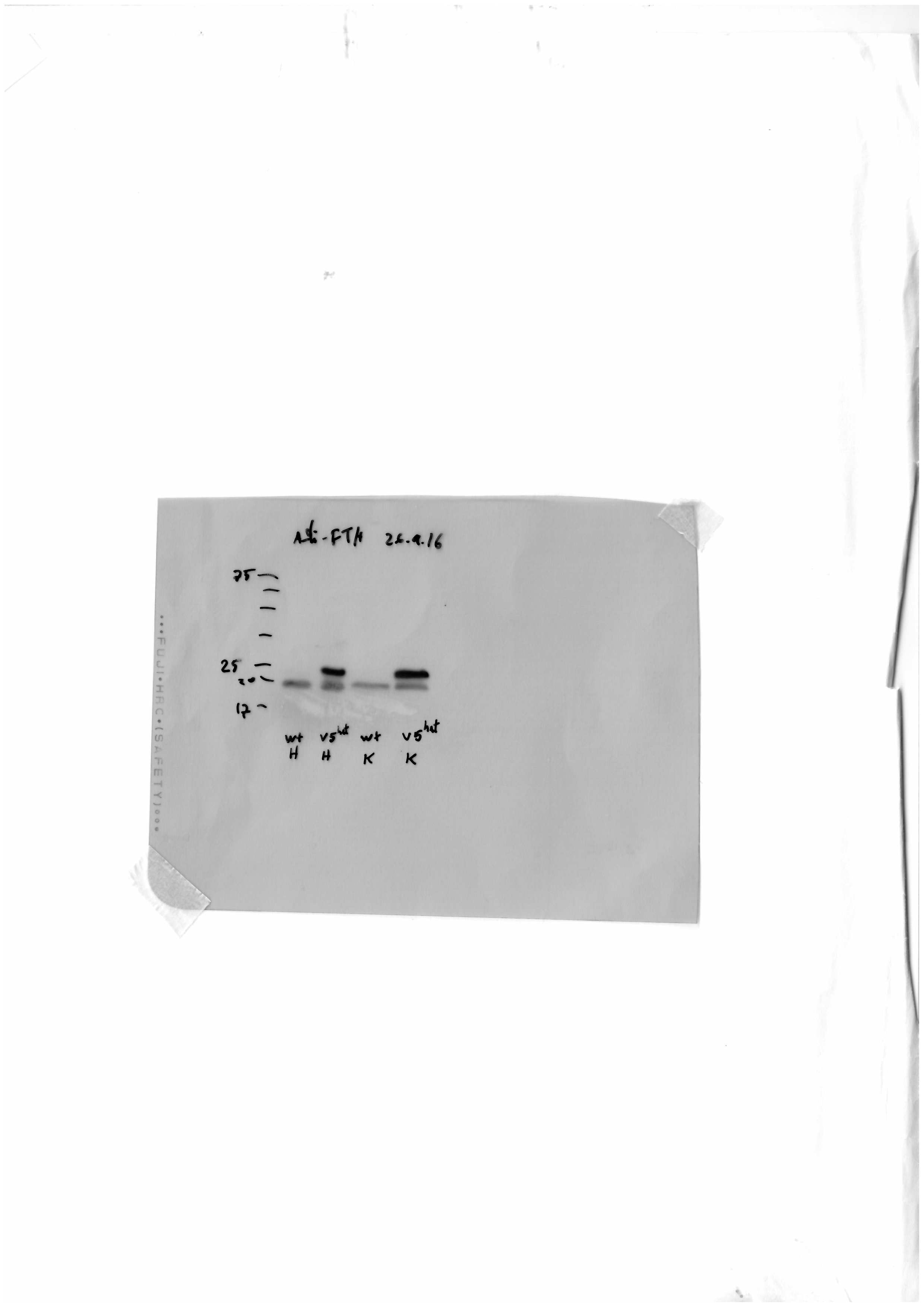

Supplement: Supplementary file 7 — Source data Fig. 5 [file 44318_2025_622_MOESM7_ESM.zip › Figure 5/Fig. 5D/fth wt v5.tif]

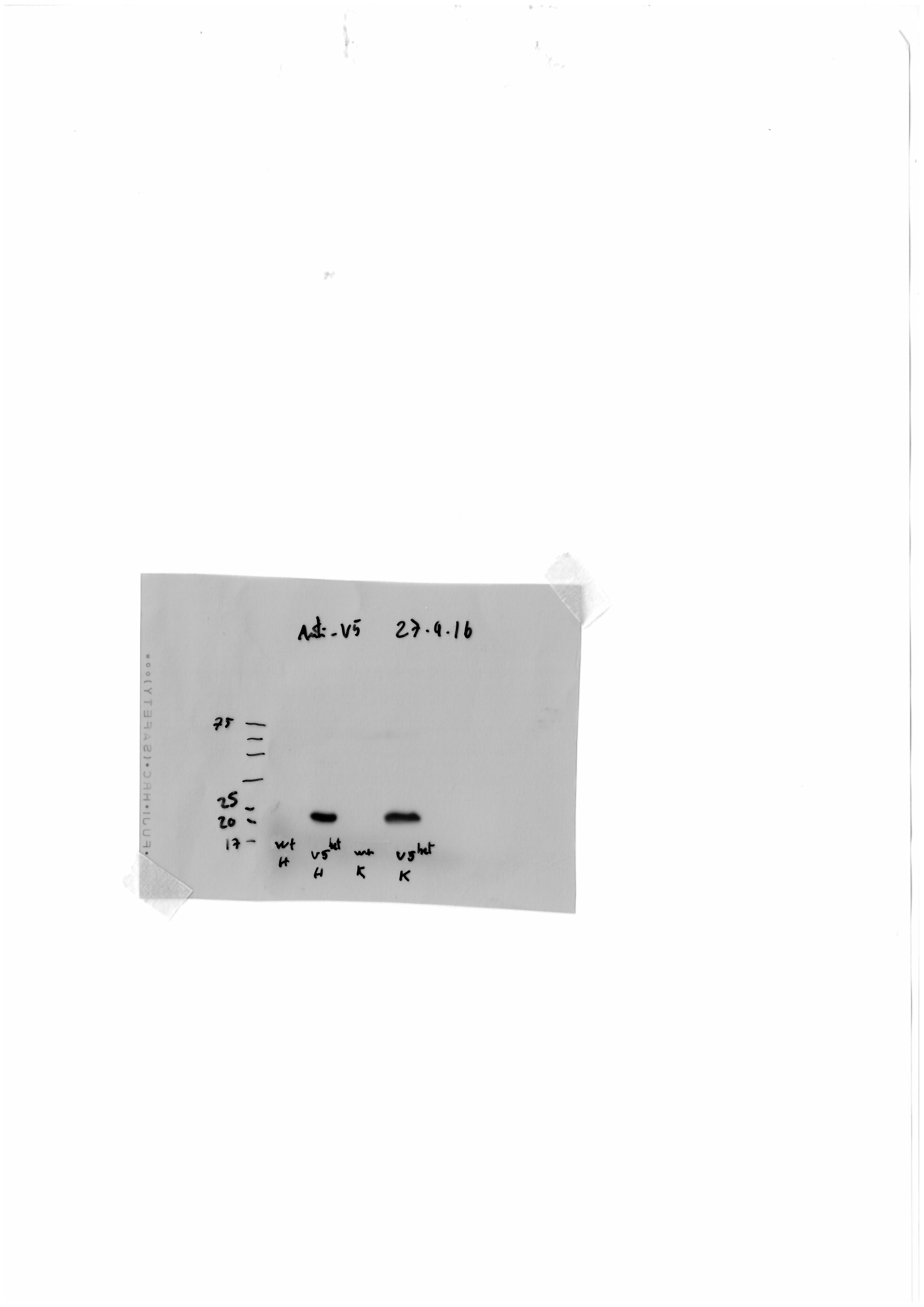

Supplement: Supplementary file 7 — Source data Fig. 5 [file 44318_2025_622_MOESM7_ESM.zip › Figure 5/Fig. 5D/v5 wt v5.tif]

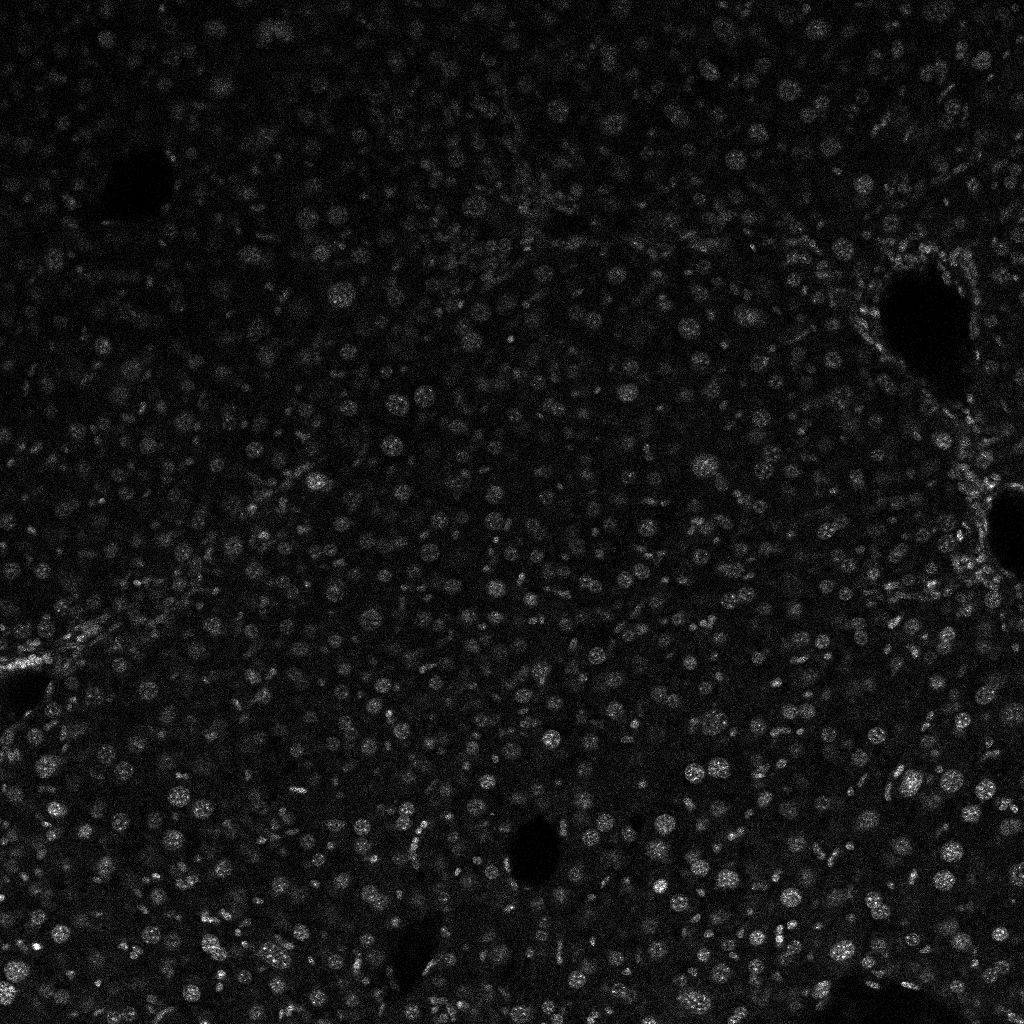

Supplement: Supplementary file 7 — Source data Fig. 5 [file 44318_2025_622_MOESM7_ESM.zip › Figure 5/Fig. 5F/26-7-17 v5 bm immuno _liver v5--del___v5+fth 2_RAW_ch00.tif]

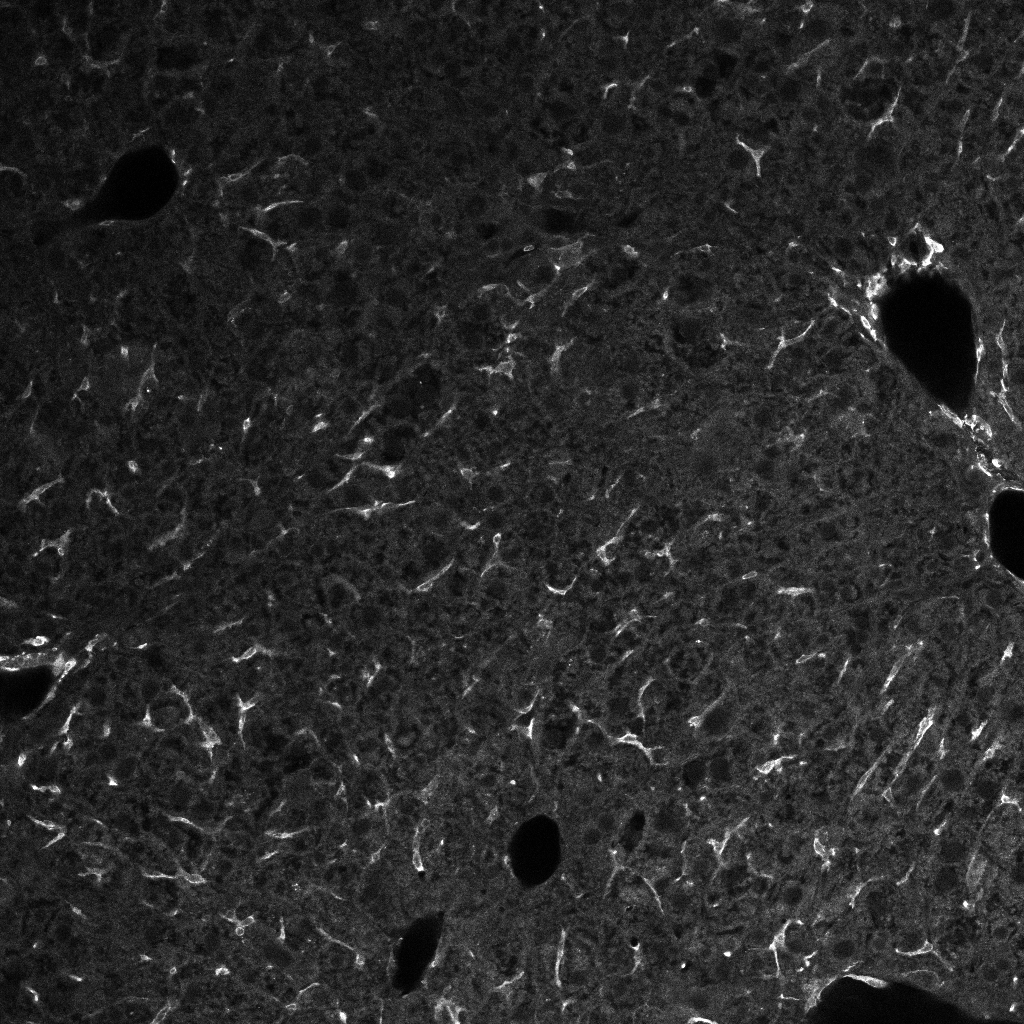

Supplement: Supplementary file 7 — Source data Fig. 5 [file 44318_2025_622_MOESM7_ESM.zip › Figure 5/Fig. 5F/26-7-17 v5 bm immuno _liver v5--del___v5+fth 2_RAW_ch02.tif]

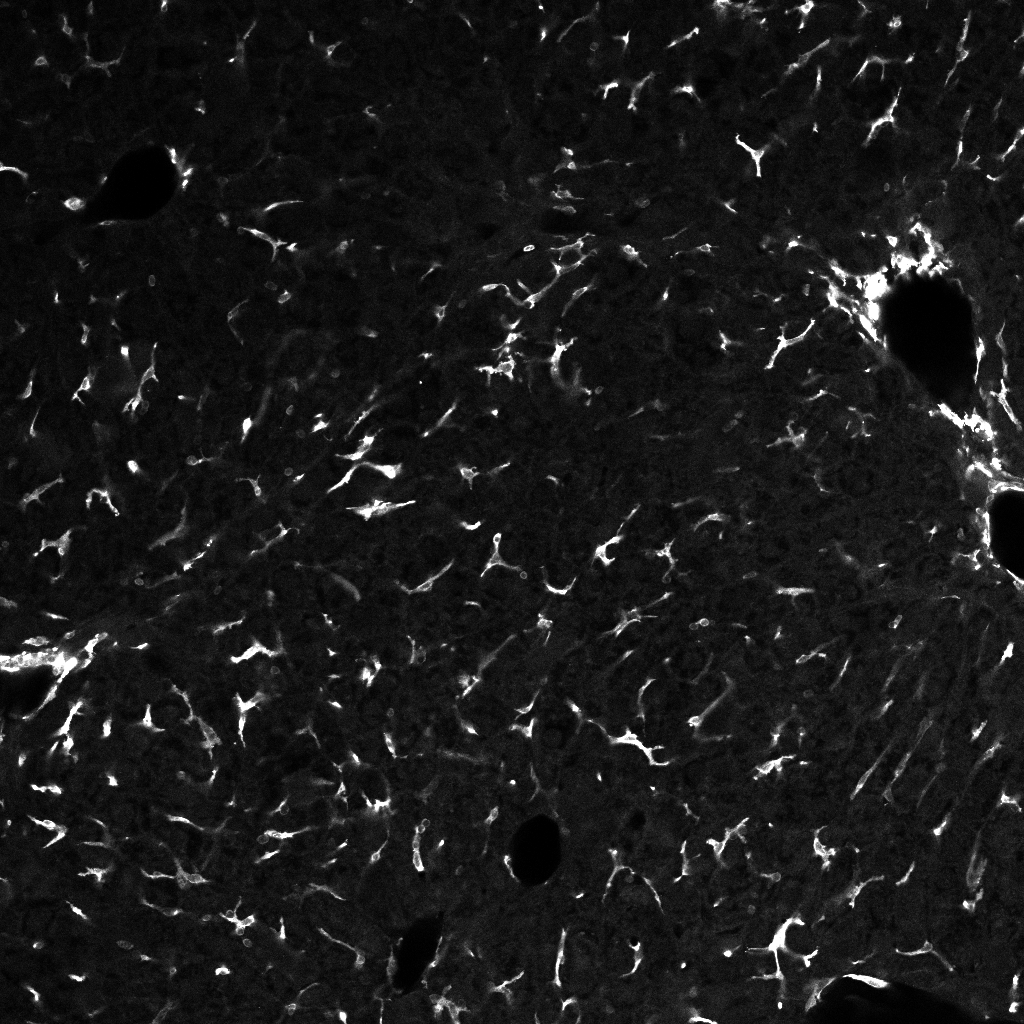

Supplement: Supplementary file 7 — Source data Fig. 5 [file 44318_2025_622_MOESM7_ESM.zip › Figure 5/Fig. 5F/26-7-17 v5 bm immuno _liver v5--del___v5+fth 2_RAW_ch03.tif]

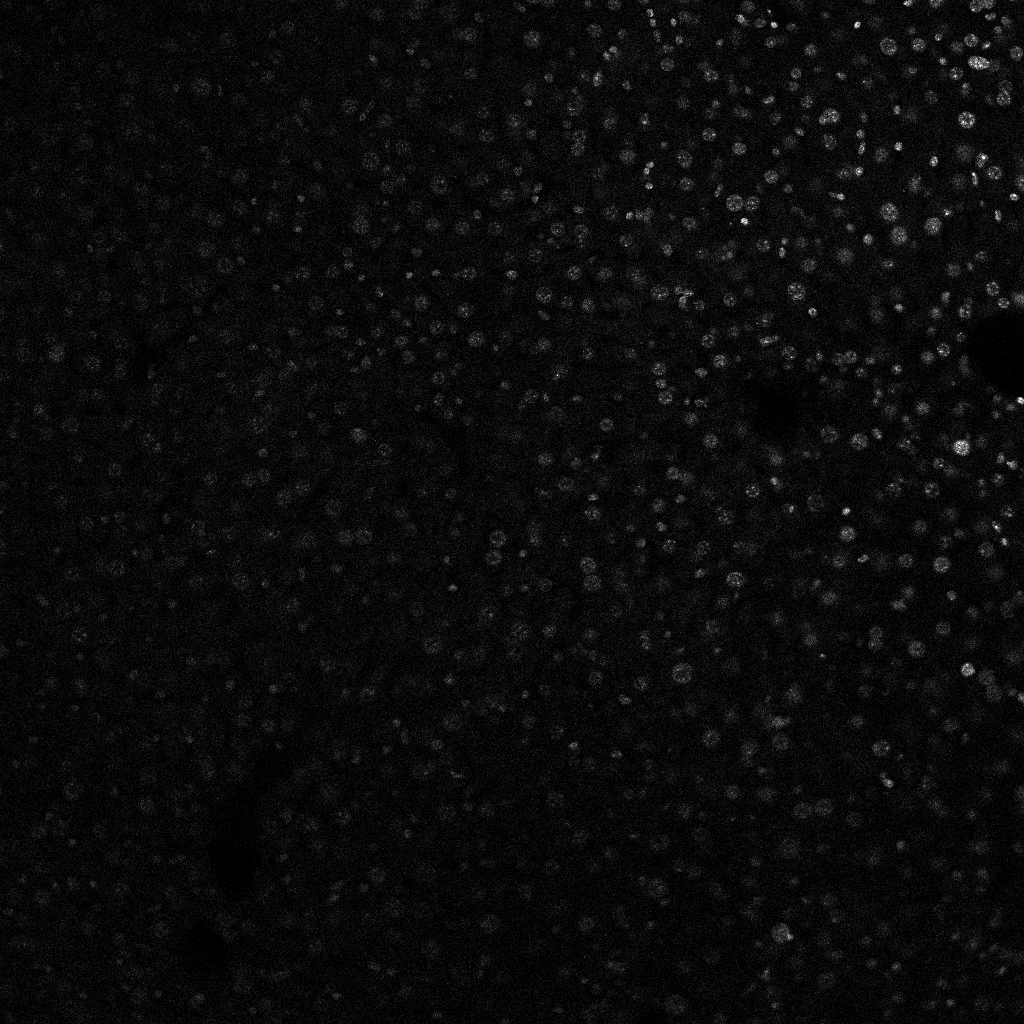

Supplement: Supplementary file 7 — Source data Fig. 5 [file 44318_2025_622_MOESM7_ESM.zip › Figure 5/Fig. 5F/26-7-17 v5 bm immuno _liver v5--wt___v5+fth 1_RAW_ch00.tif]

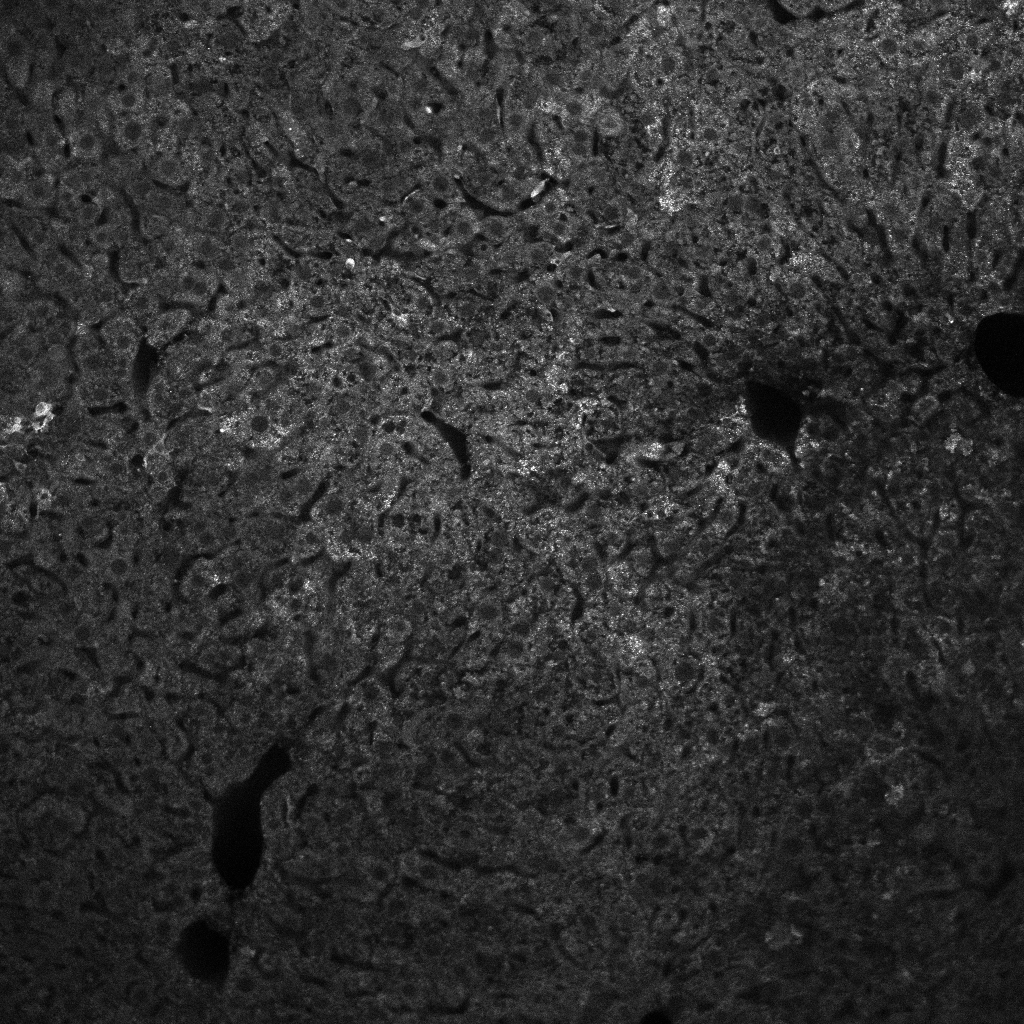

Supplement: Supplementary file 7 — Source data Fig. 5 [file 44318_2025_622_MOESM7_ESM.zip › Figure 5/Fig. 5F/26-7-17 v5 bm immuno _liver v5--wt___v5+fth 1_RAW_ch02.tif]

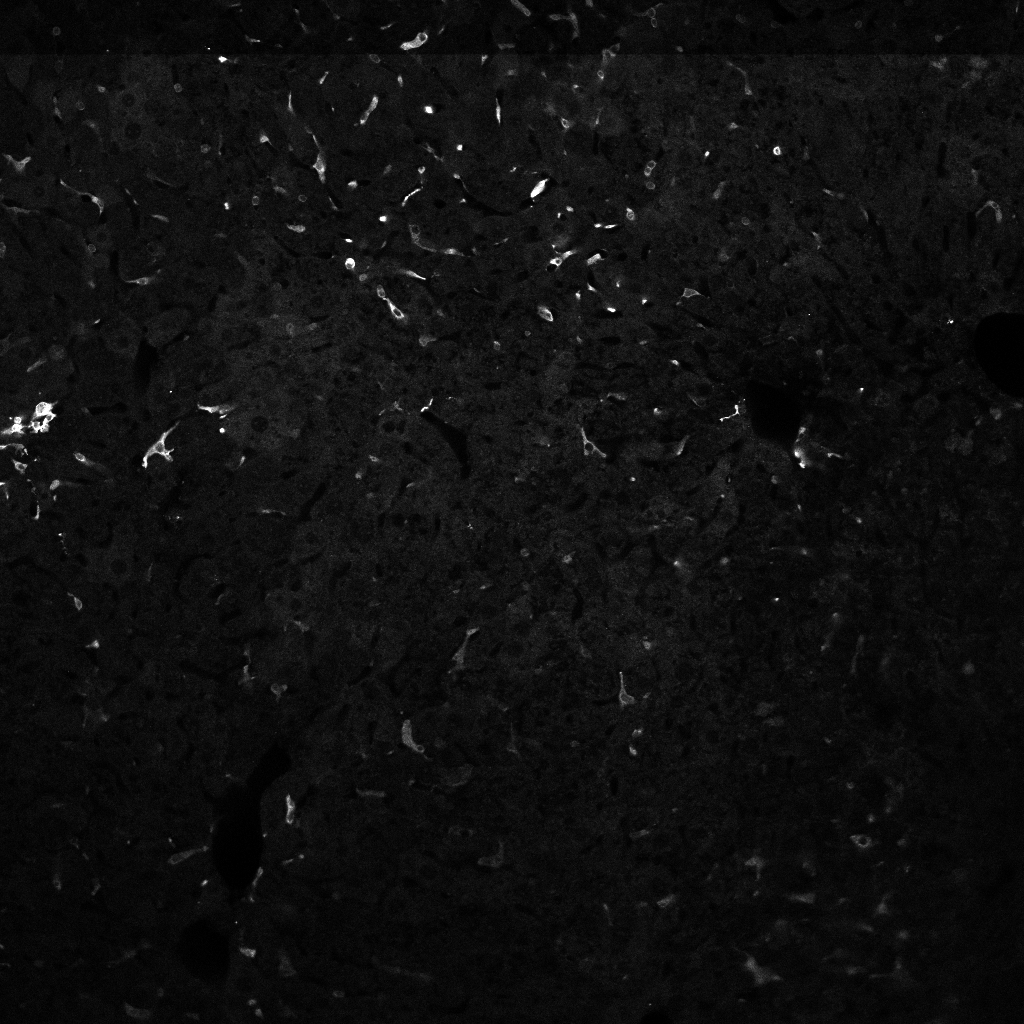

Supplement: Supplementary file 7 — Source data Fig. 5 [file 44318_2025_622_MOESM7_ESM.zip › Figure 5/Fig. 5F/26-7-17 v5 bm immuno _liver v5--wt___v5+fth 1_RAW_ch03.tif]

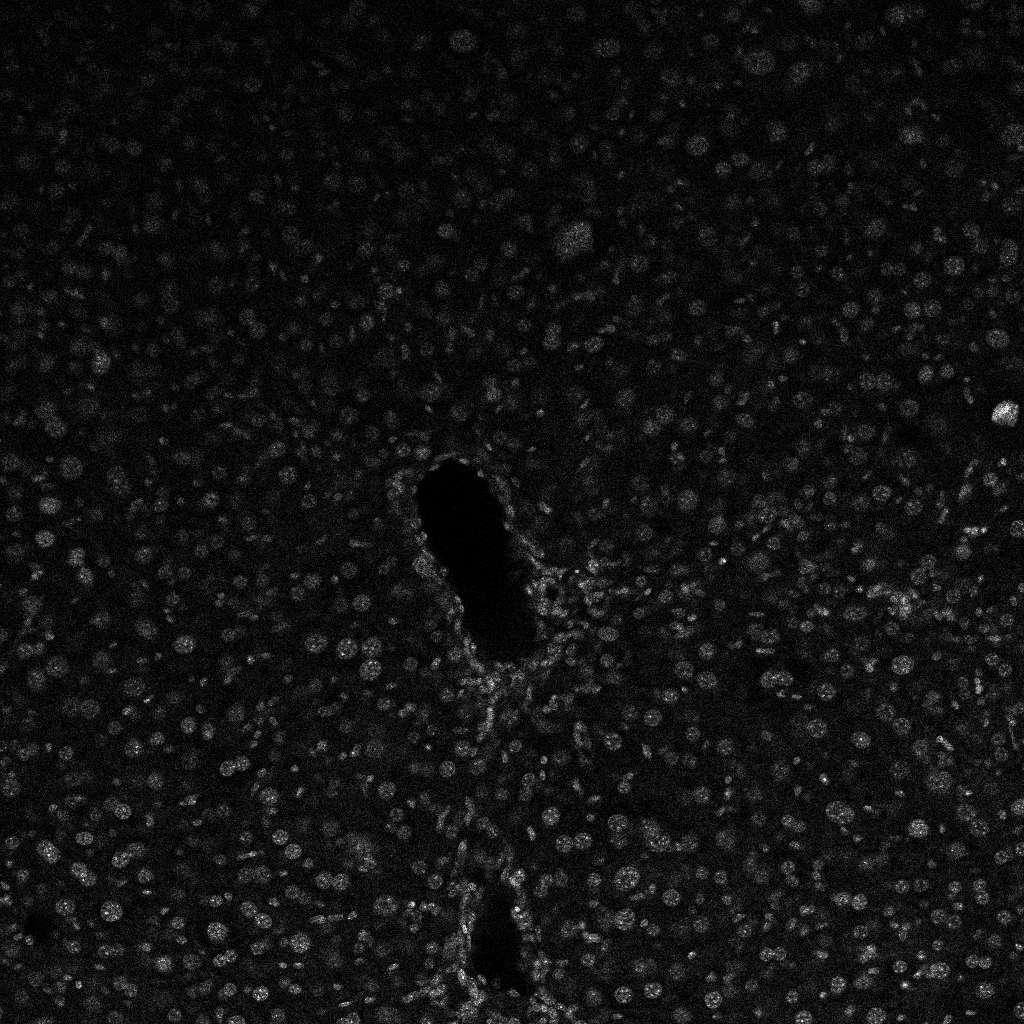

Supplement: Supplementary file 7 — Source data Fig. 5 [file 44318_2025_622_MOESM7_ESM.zip › Figure 5/Fig. 5G/26-7-17 v5 bm immuno _liver v5--del___v5+cd68 1_RAW_ch00.tif]

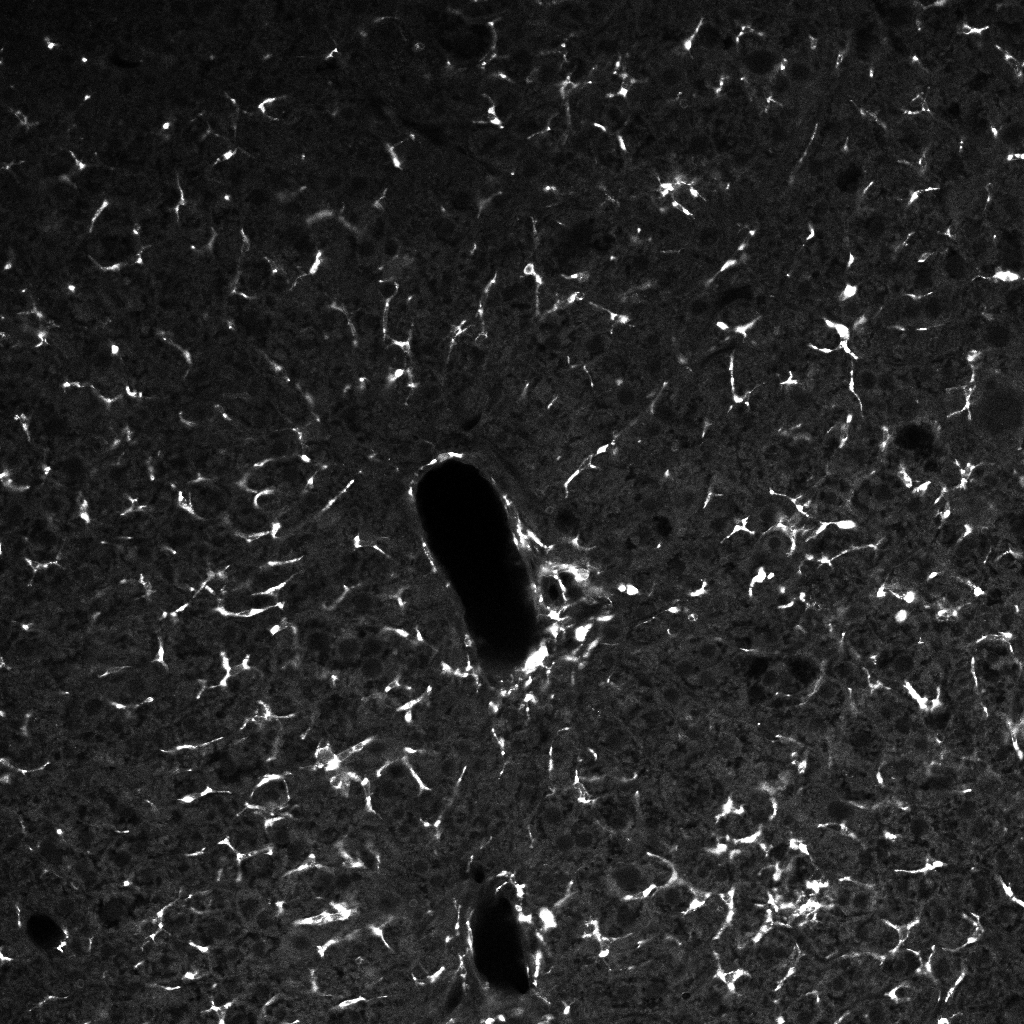

Supplement: Supplementary file 7 — Source data Fig. 5 [file 44318_2025_622_MOESM7_ESM.zip › Figure 5/Fig. 5G/26-7-17 v5 bm immuno _liver v5--del___v5+cd68 1_RAW_ch02.tif]

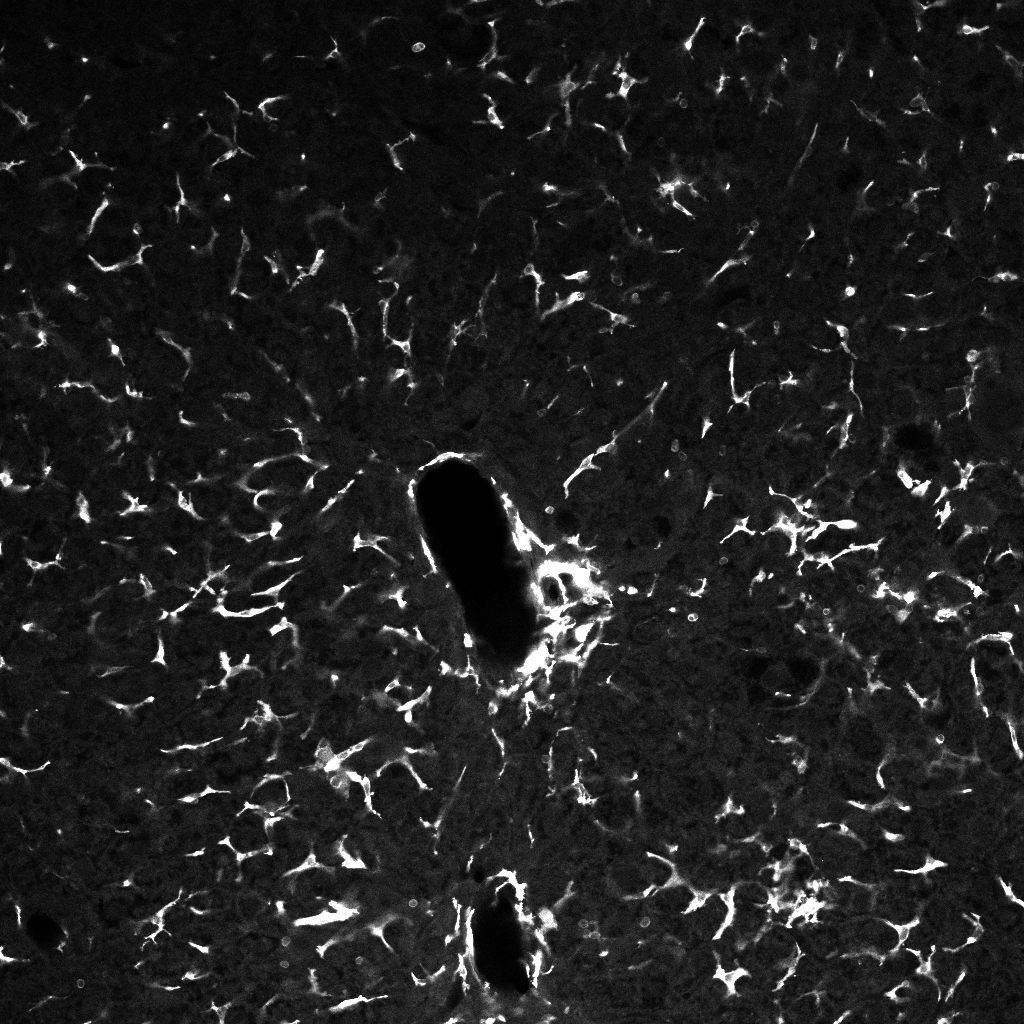

Supplement: Supplementary file 7 — Source data Fig. 5 [file 44318_2025_622_MOESM7_ESM.zip › Figure 5/Fig. 5G/26-7-17 v5 bm immuno _liver v5--del___v5+cd68 1_RAW_ch03.tif]

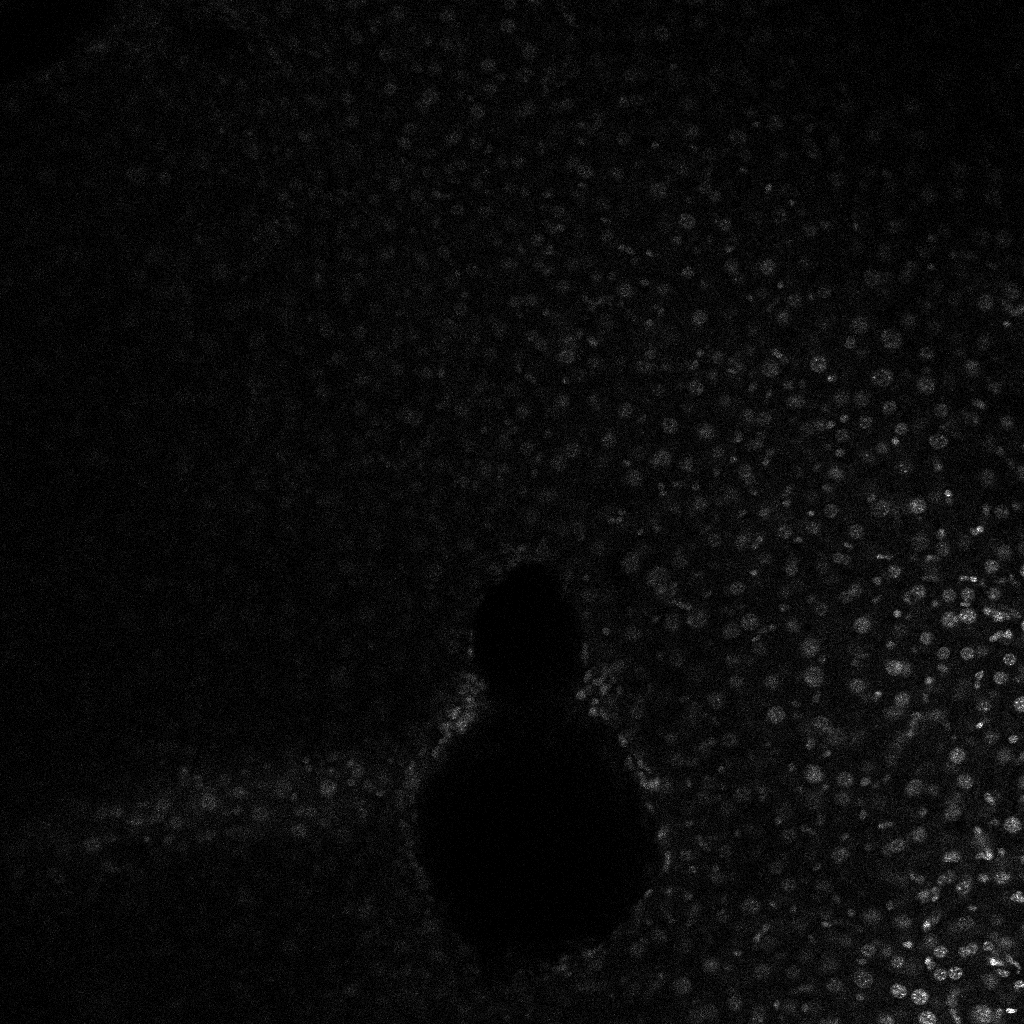

Supplement: Supplementary file 7 — Source data Fig. 5 [file 44318_2025_622_MOESM7_ESM.zip › Figure 5/Fig. 5G/26-7-17 v5 bm immuno _liver v5--wt___v5+cd68 1_RAW_ch00.tif]

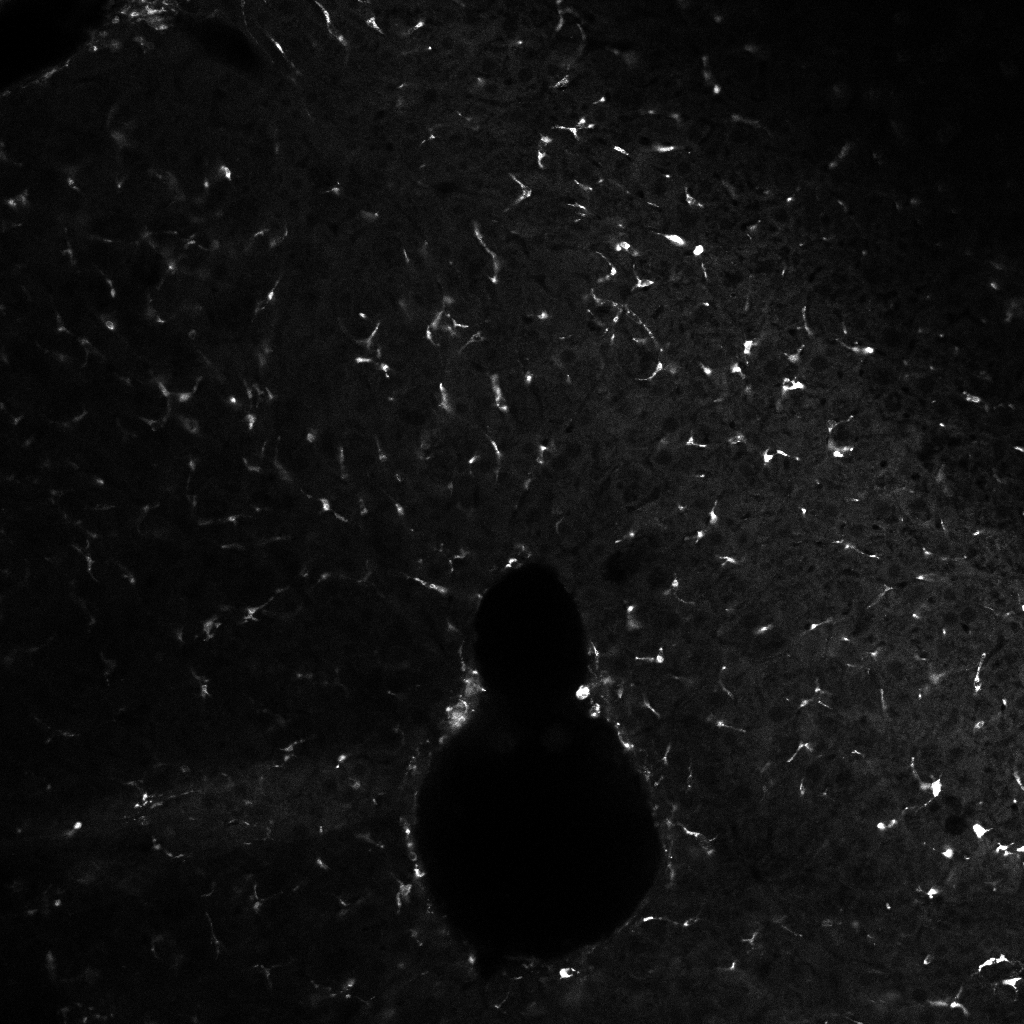

Supplement: Supplementary file 7 — Source data Fig. 5 [file 44318_2025_622_MOESM7_ESM.zip › Figure 5/Fig. 5G/26-7-17 v5 bm immuno _liver v5--wt___v5+cd68 1_RAW_ch02.tif]

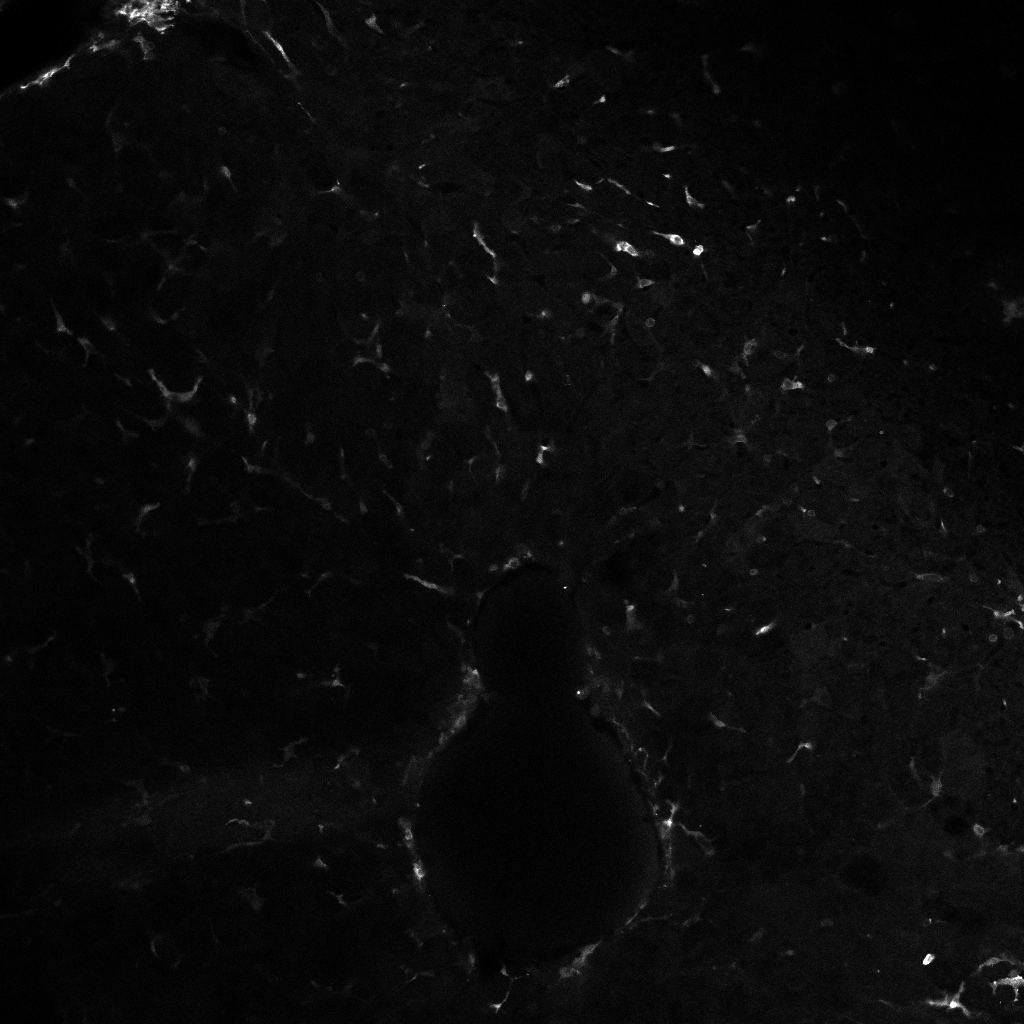

Supplement: Supplementary file 7 — Source data Fig. 5 [file 44318_2025_622_MOESM7_ESM.zip › Figure 5/Fig. 5G/26-7-17 v5 bm immuno _liver v5--wt___v5+cd68 1_RAW_ch03.tif]

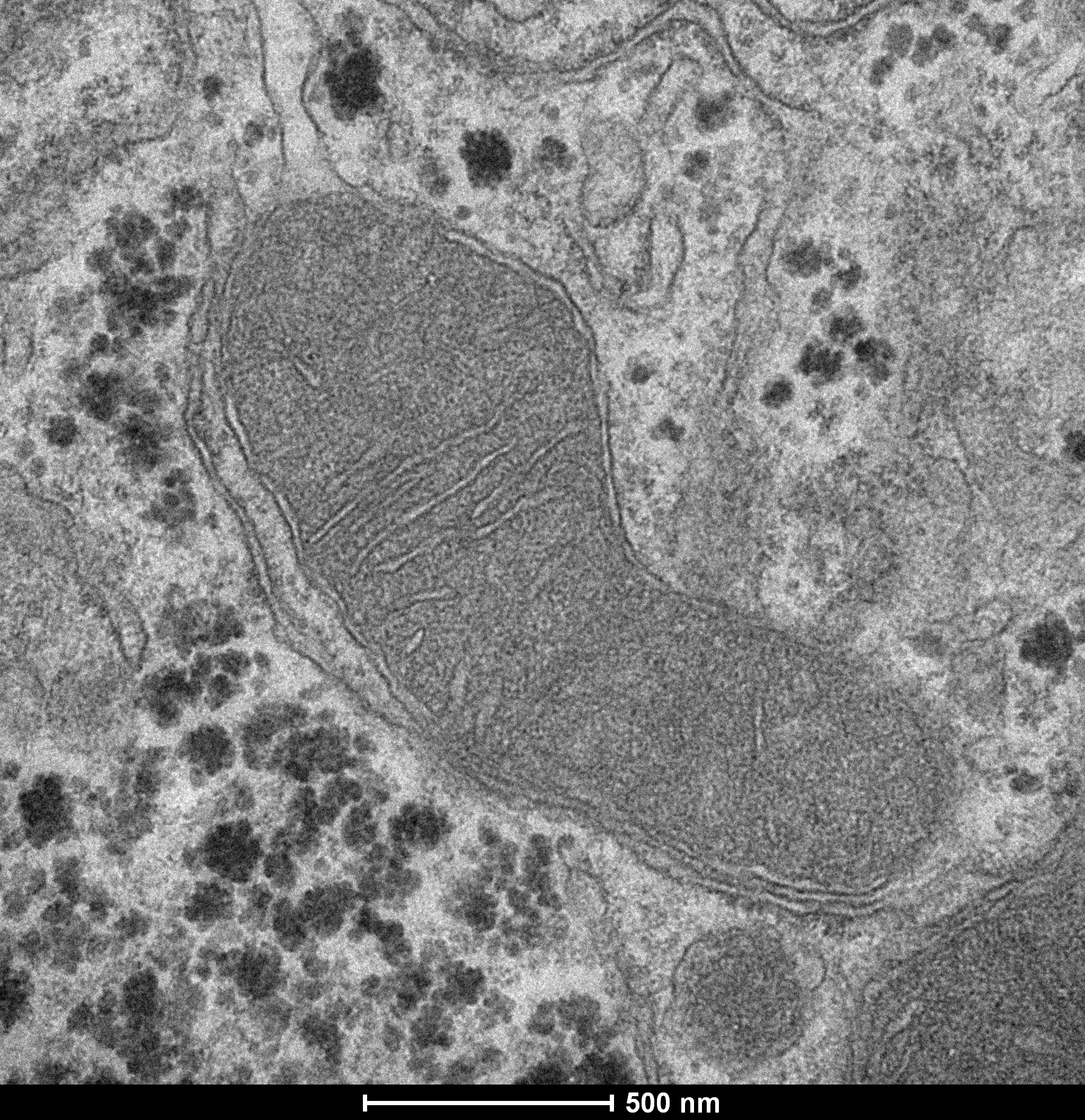

Supplement: Supplementary file 8 — Source data Fig. 6 [file 44318_2025_622_MOESM8_ESM.zip › Figure 6/Fig. 6D/Liver_1_Rescued_Early_B4_43k_4.tif]

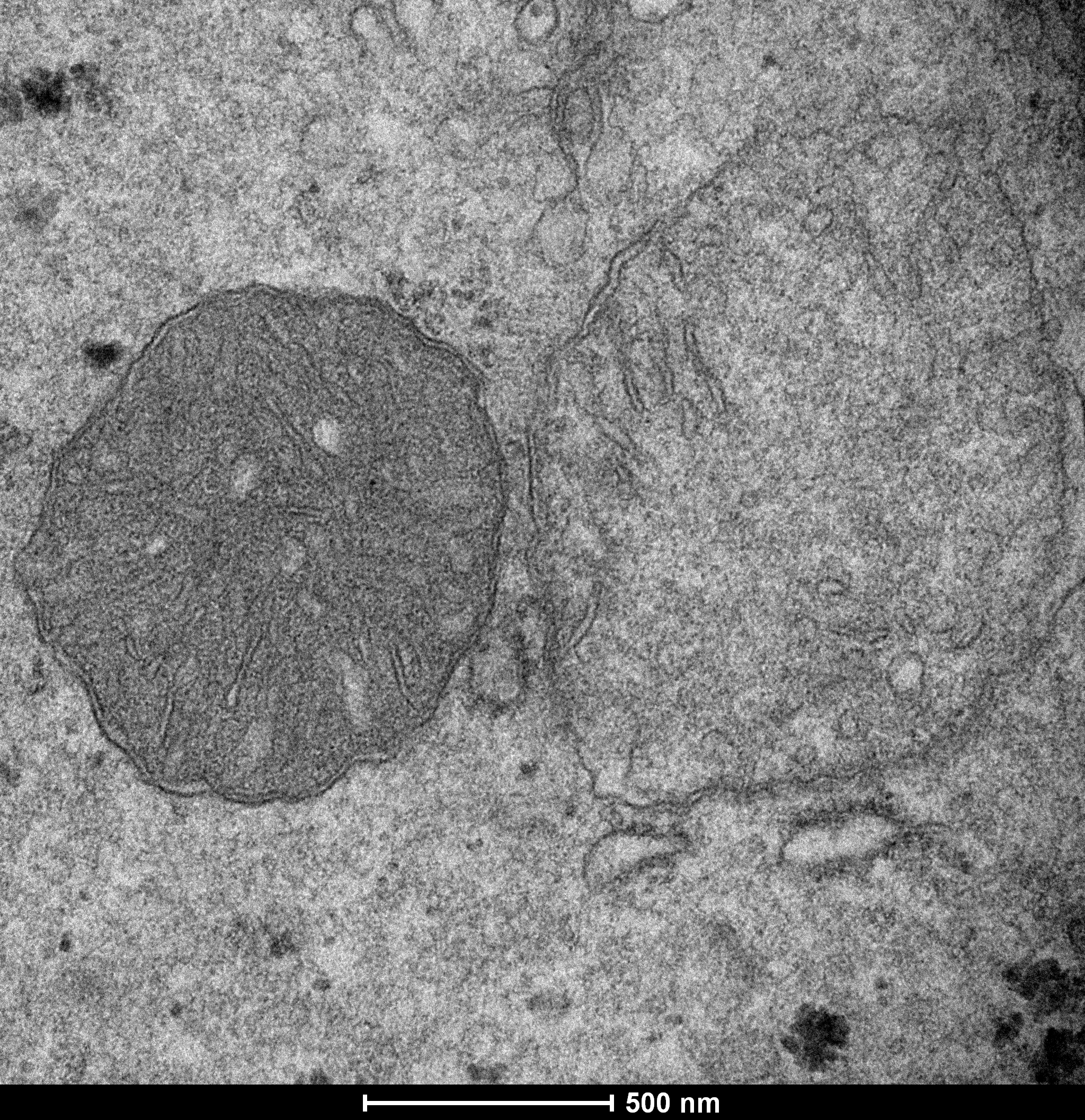

Supplement: Supplementary file 8 — Source data Fig. 6 [file 44318_2025_622_MOESM8_ESM.zip › Figure 6/Fig. 6D/Liver_4_LysM_late_O4_43k_8.tif]

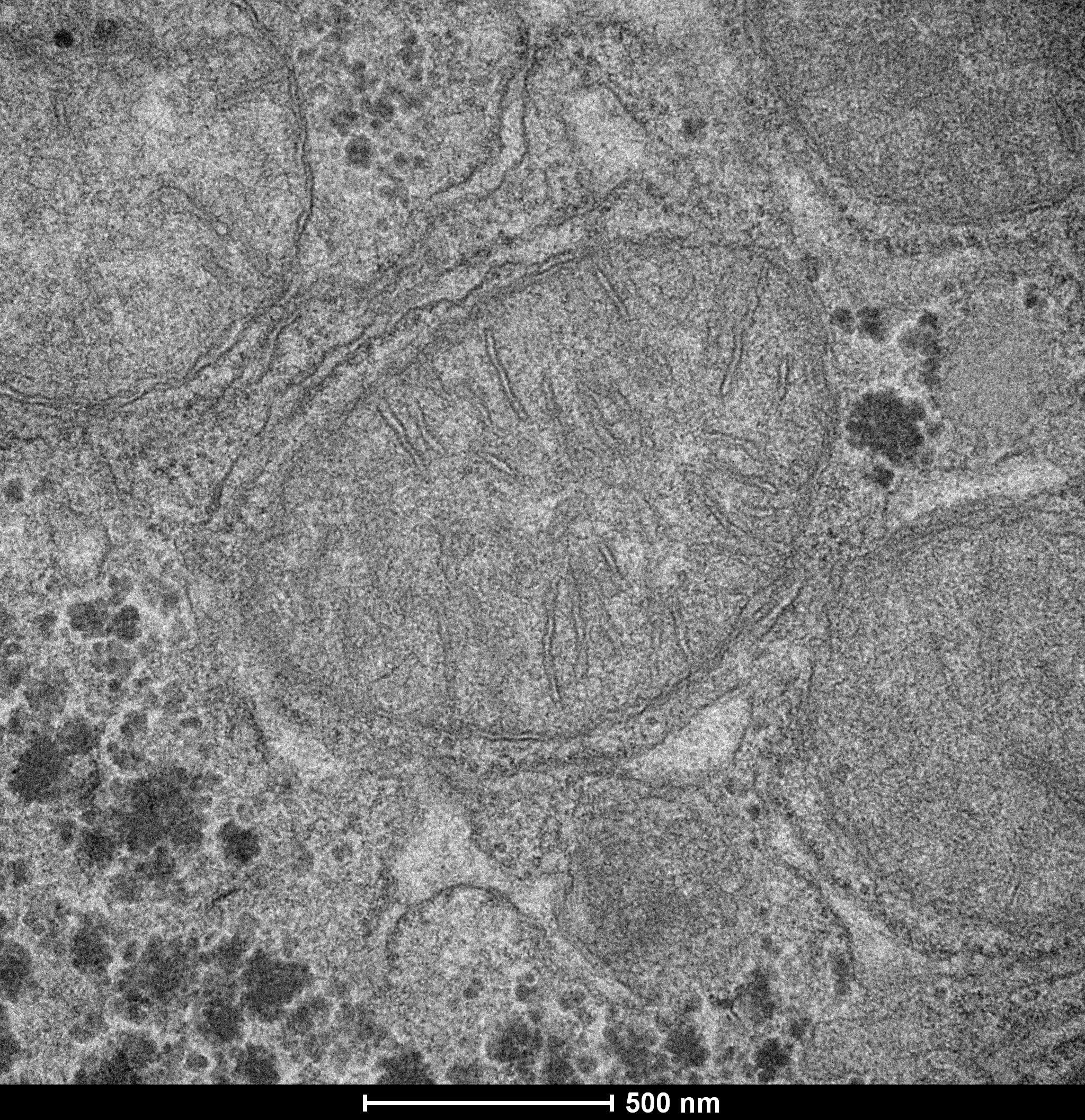

Supplement: Supplementary file 8 — Source data Fig. 6 [file 44318_2025_622_MOESM8_ESM.zip › Figure 6/Fig. 6D/Liver1_Q2_B6711A_43k_02b.tif]

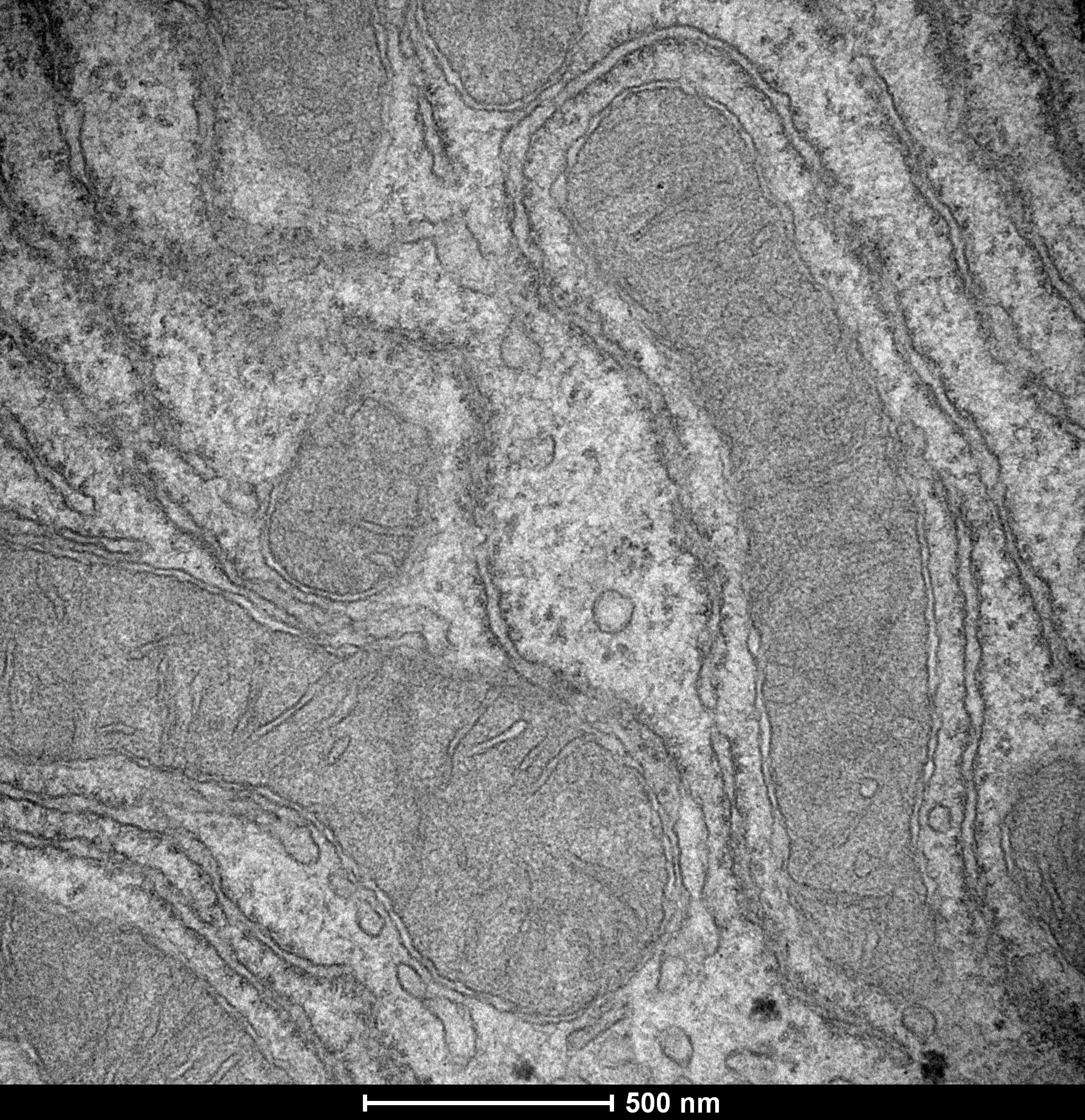

Supplement: Supplementary file 8 — Source data Fig. 6 [file 44318_2025_622_MOESM8_ESM.zip › Figure 6/Fig. 6D/Liver2_b6411B_011_43k.tif]

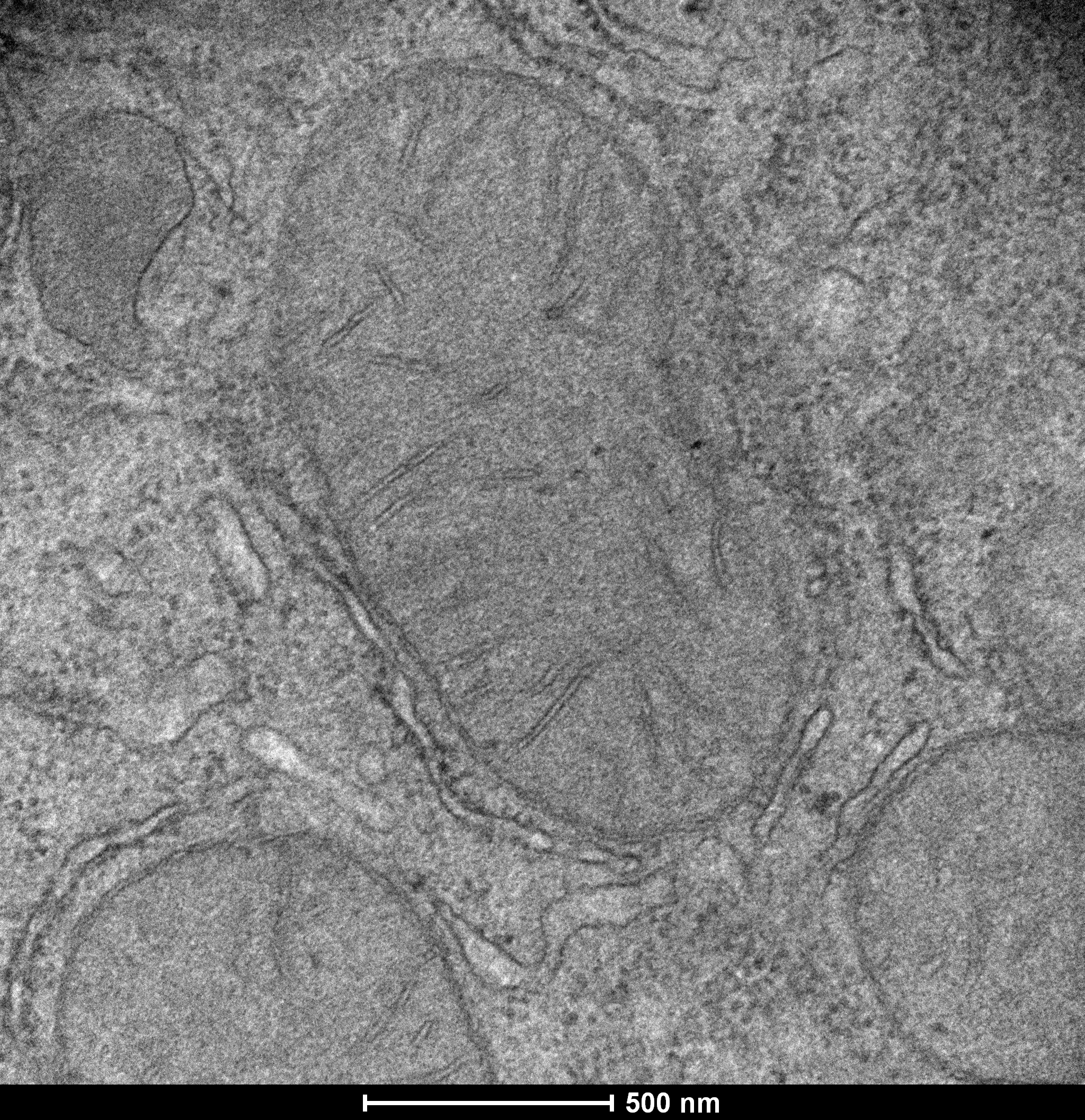

Supplement: Supplementary file 8 — Source data Fig. 6 [file 44318_2025_622_MOESM8_ESM.zip › Figure 6/Fig. 6D/Liver3_K4_6411B_43k_01c.tif]

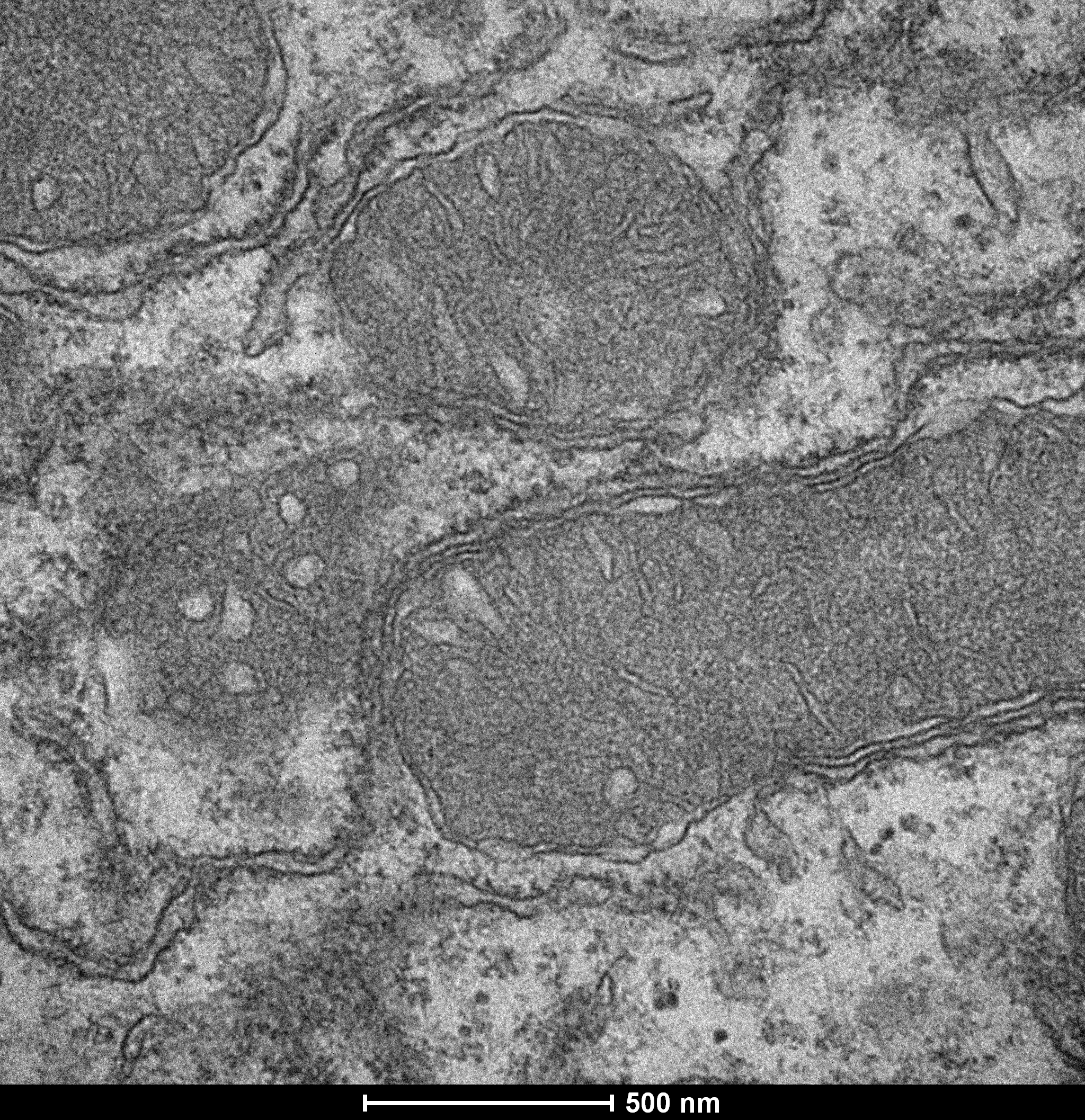

Supplement: Supplementary file 8 — Source data Fig. 6 [file 44318_2025_622_MOESM8_ESM.zip › Figure 6/Fig. 6D/Liver3_S2_B6711A_43k_05.tif]

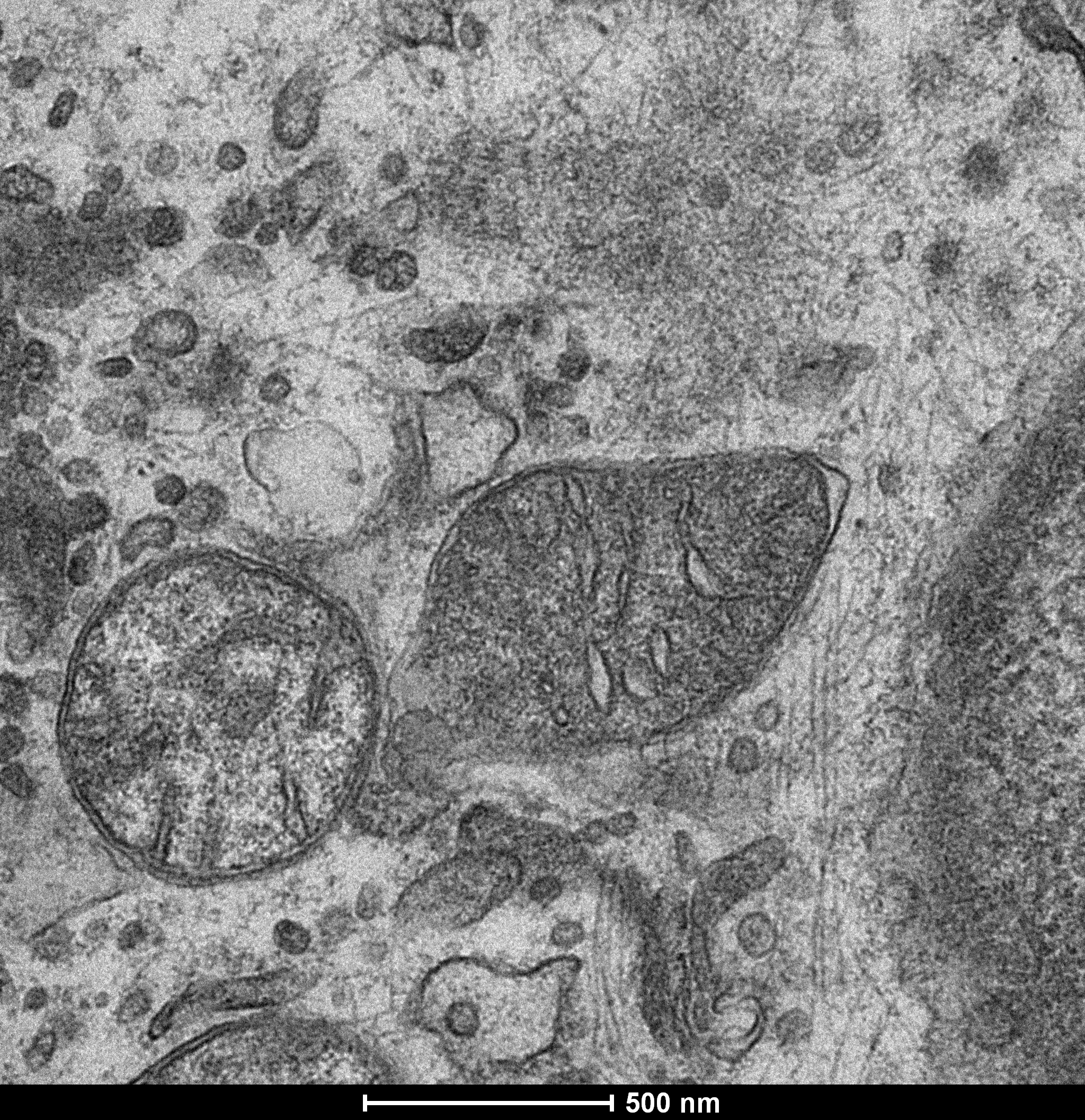

Supplement: Supplementary file 8 — Source data Fig. 6 [file 44318_2025_622_MOESM8_ESM.zip › Figure 6/Fig. 6D/WAT_4_LysM_late_P3_43k_5.tif]

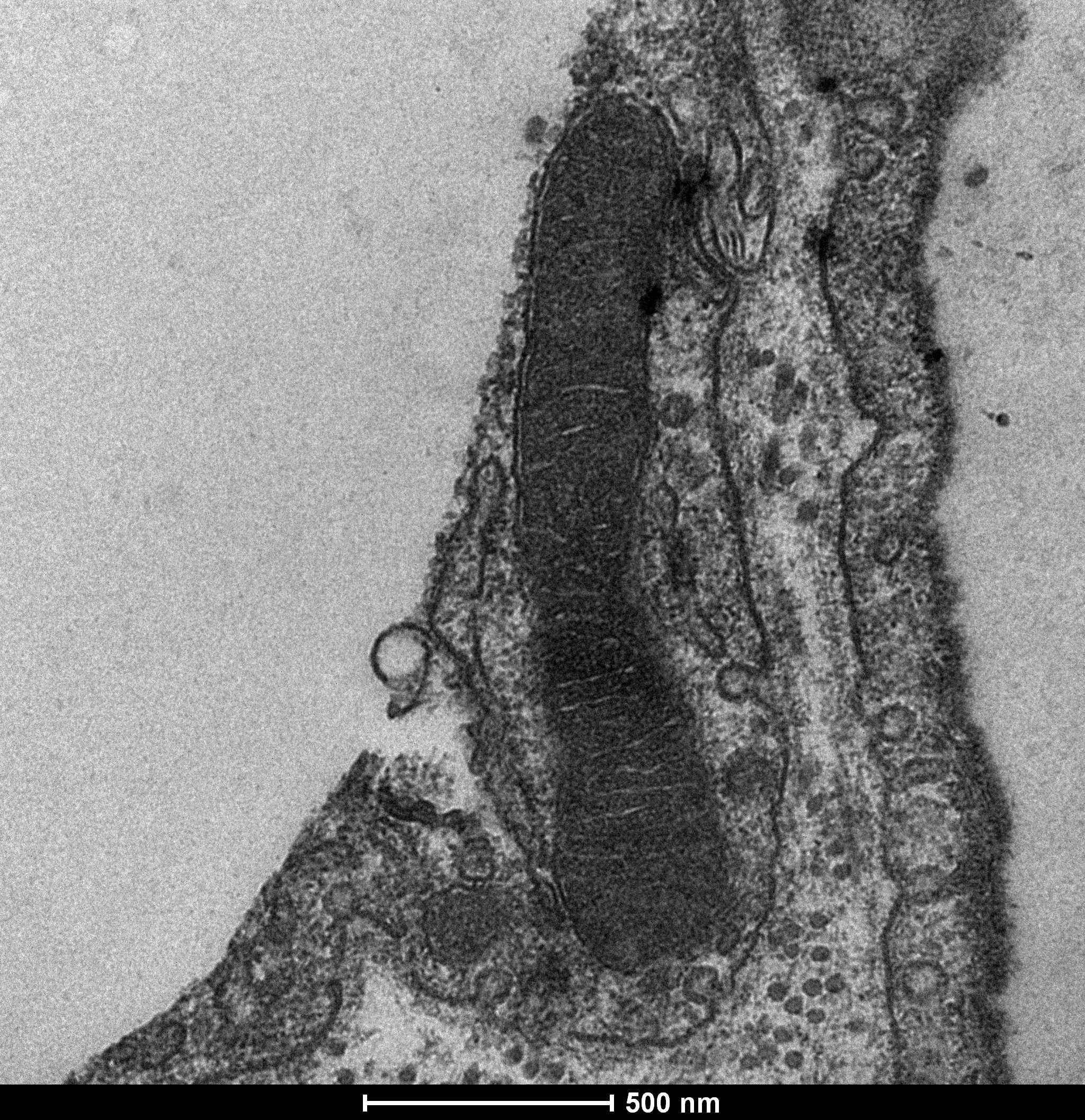

Supplement: Supplementary file 8 — Source data Fig. 6 [file 44318_2025_622_MOESM8_ESM.zip › Figure 6/Fig. 6D/WAT1_Q10_B6707A_43k_02.tif]

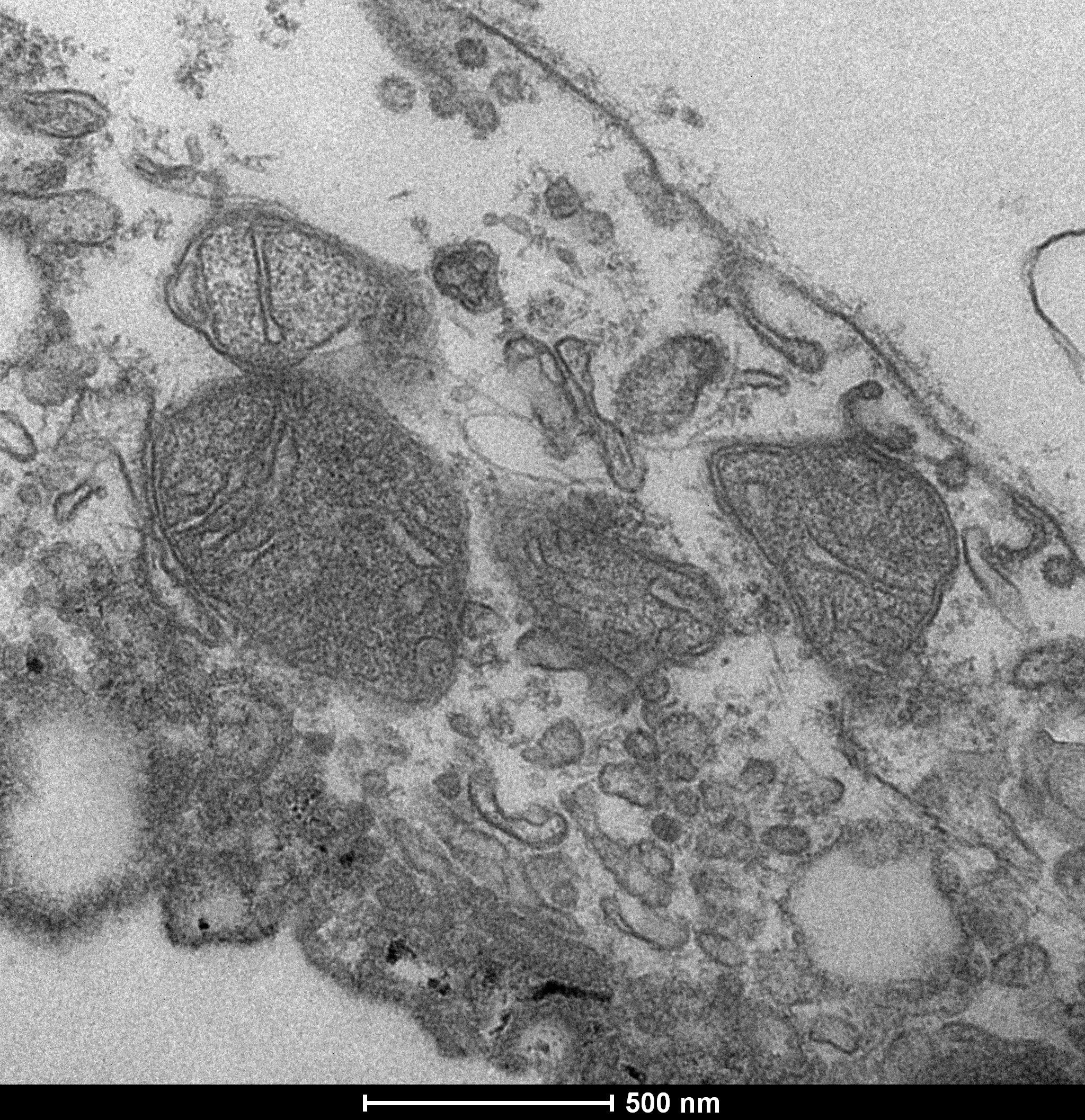

Supplement: Supplementary file 8 — Source data Fig. 6 [file 44318_2025_622_MOESM8_ESM.zip › Figure 6/Fig. 6D/WAT2_R9_B6715B_43k_04d.tif]

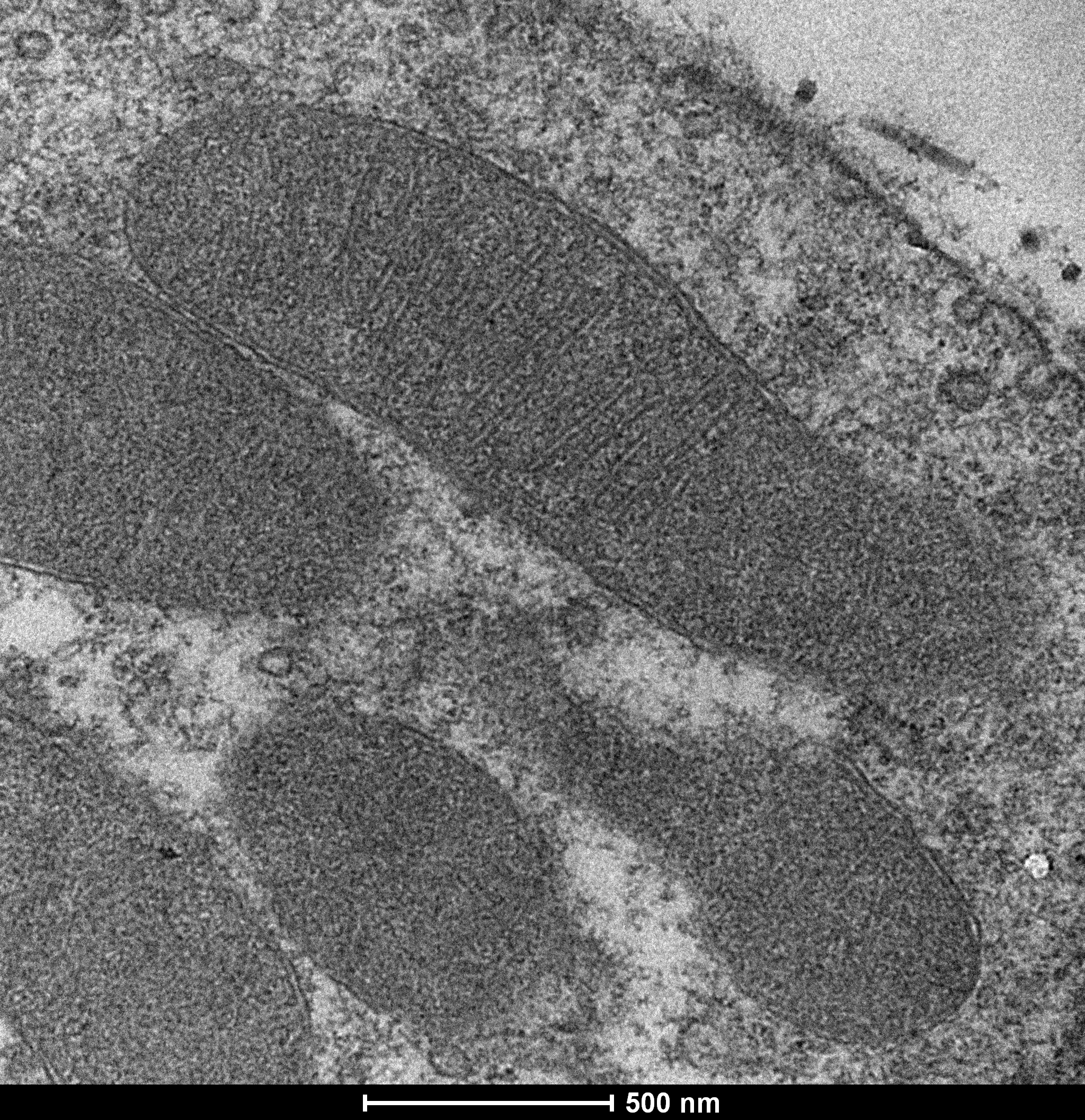

Supplement: Supplementary file 8 — Source data Fig. 6 [file 44318_2025_622_MOESM8_ESM.zip › Figure 6/Fig. 6D/WAT3_N3_6426A_43k_02.tif]

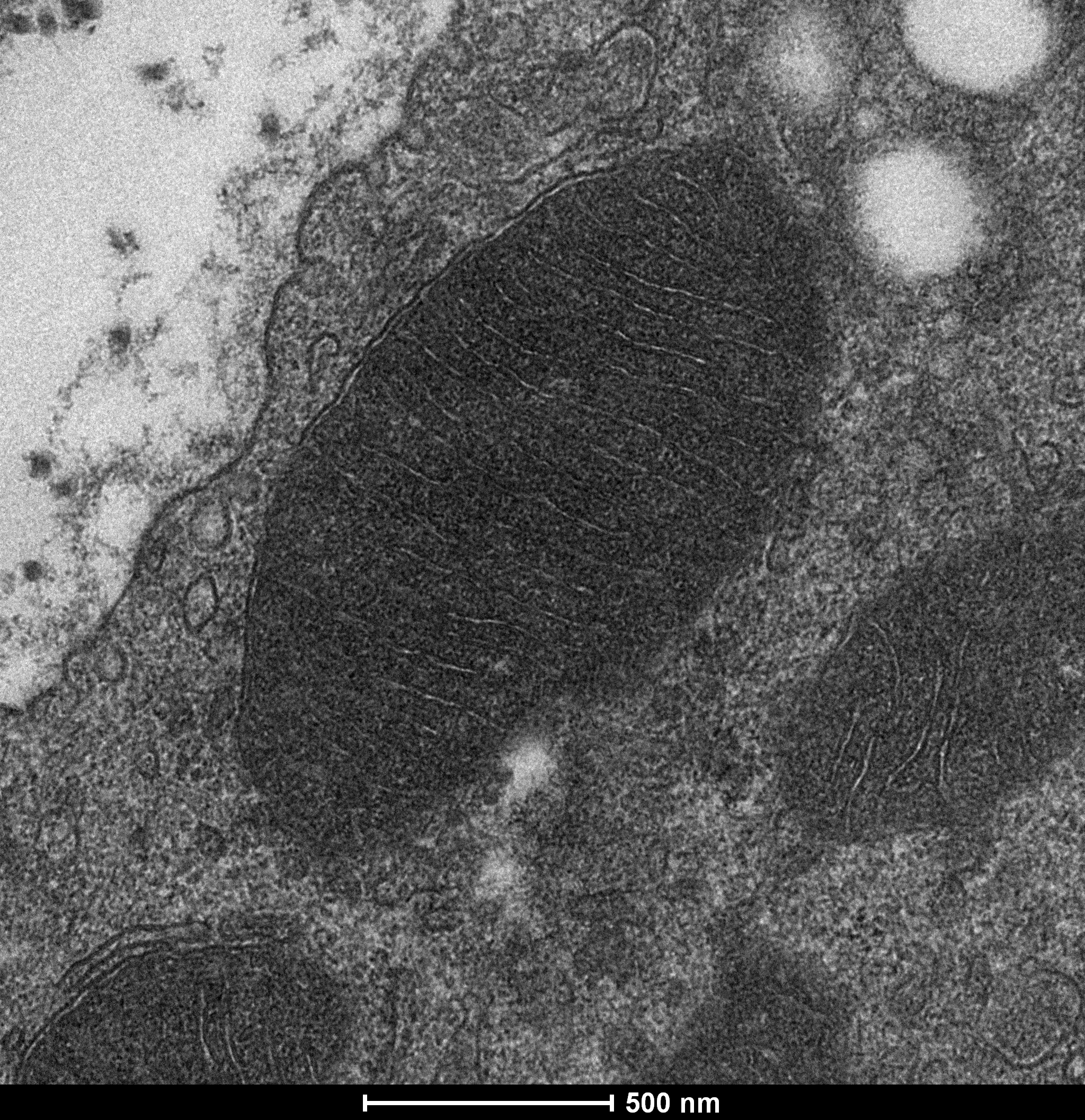

Supplement: Supplementary file 8 — Source data Fig. 6 [file 44318_2025_622_MOESM8_ESM.zip › Figure 6/Fig. 6D/WAT4_S10_B6727B_43k_02b.tif]

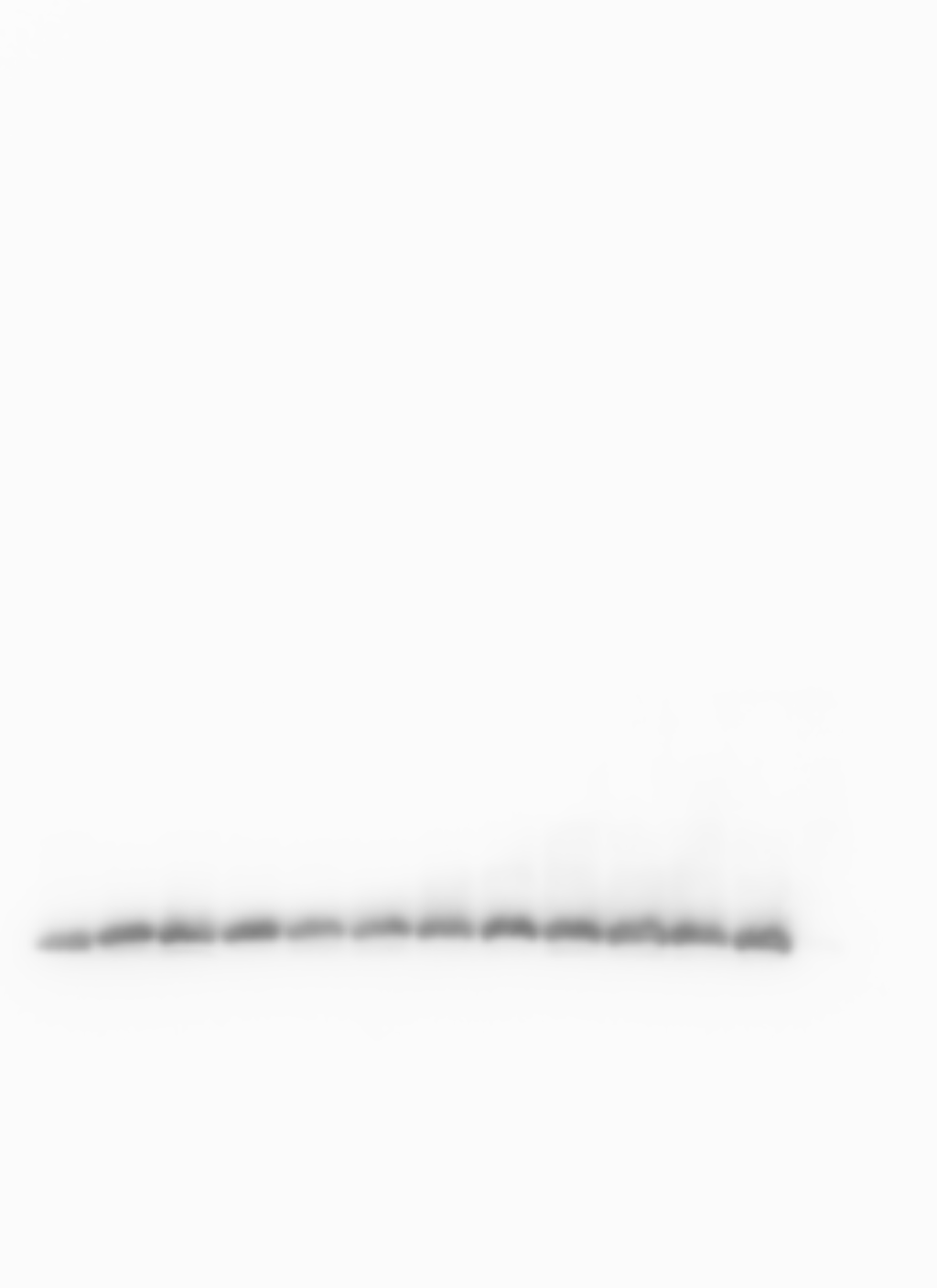

Supplement: Supplementary file 8 — Source data Fig. 6 [file 44318_2025_622_MOESM8_ESM.zip › Figure 6/Fig. 6I/H3 OXPHOS 131124 2024.11.13_08.20.06_Ch.tif]

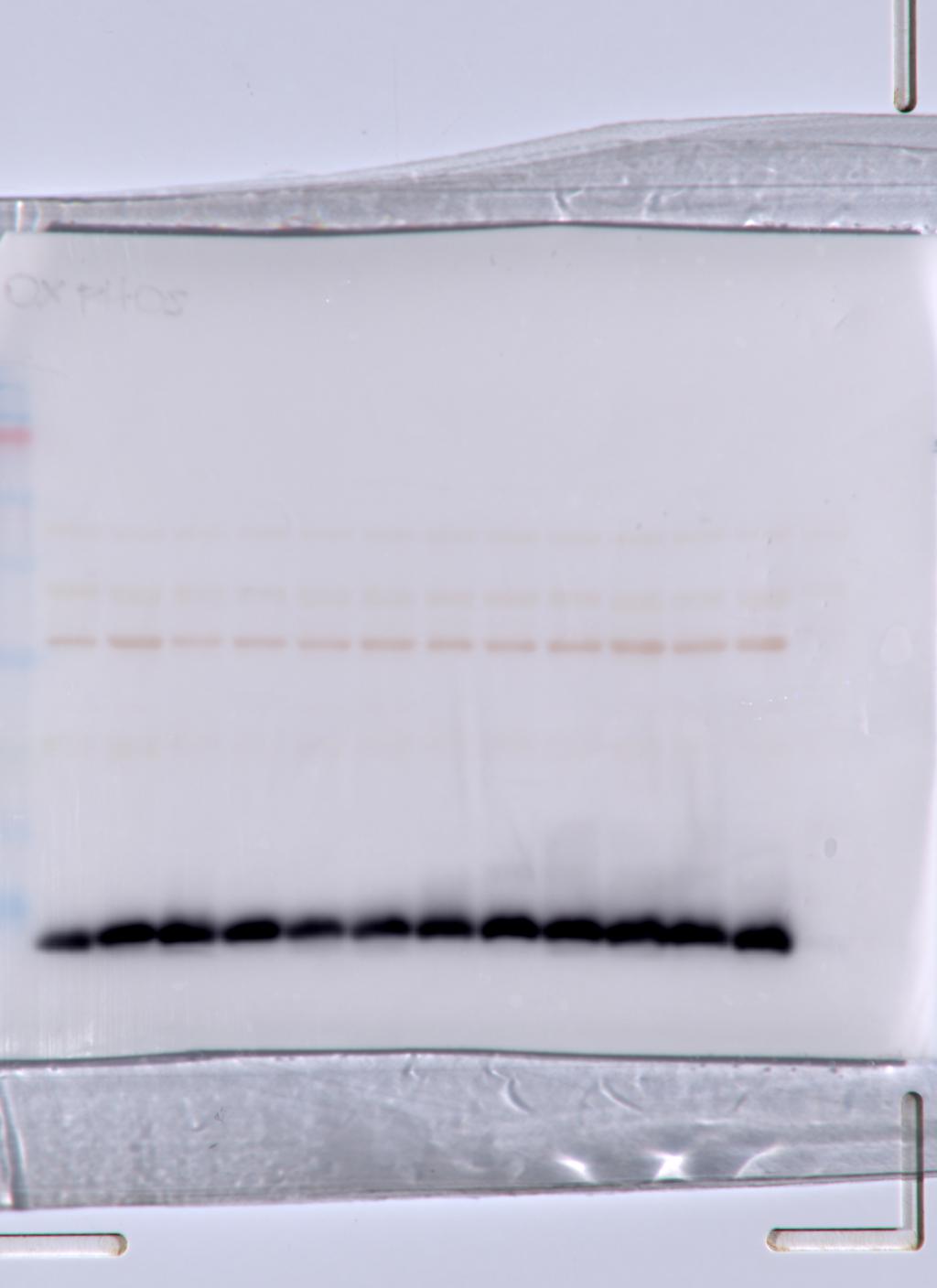

Supplement: Supplementary file 8 — Source data Fig. 6 [file 44318_2025_622_MOESM8_ESM.zip › Figure 6/Fig. 6I/H3 OXPHOS 131124 2024.11.13_08.20.06_Ch+Marker.jpg]

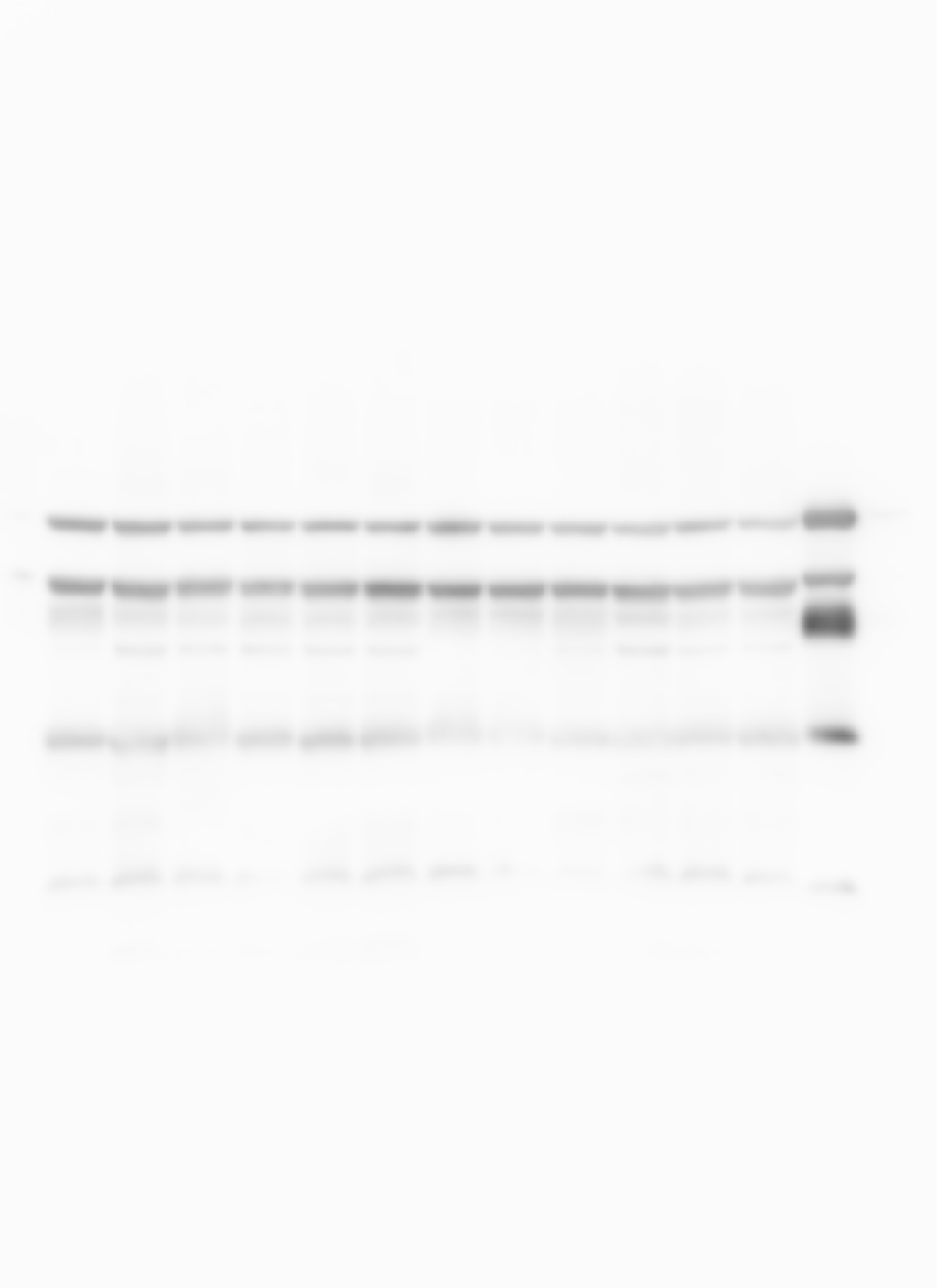

Supplement: Supplementary file 8 — Source data Fig. 6 [file 44318_2025_622_MOESM8_ESM.zip › Figure 6/Fig. 6I/OXPHOS 12% gel 011124 2024.11.01_10.49.42_Ch.tif]

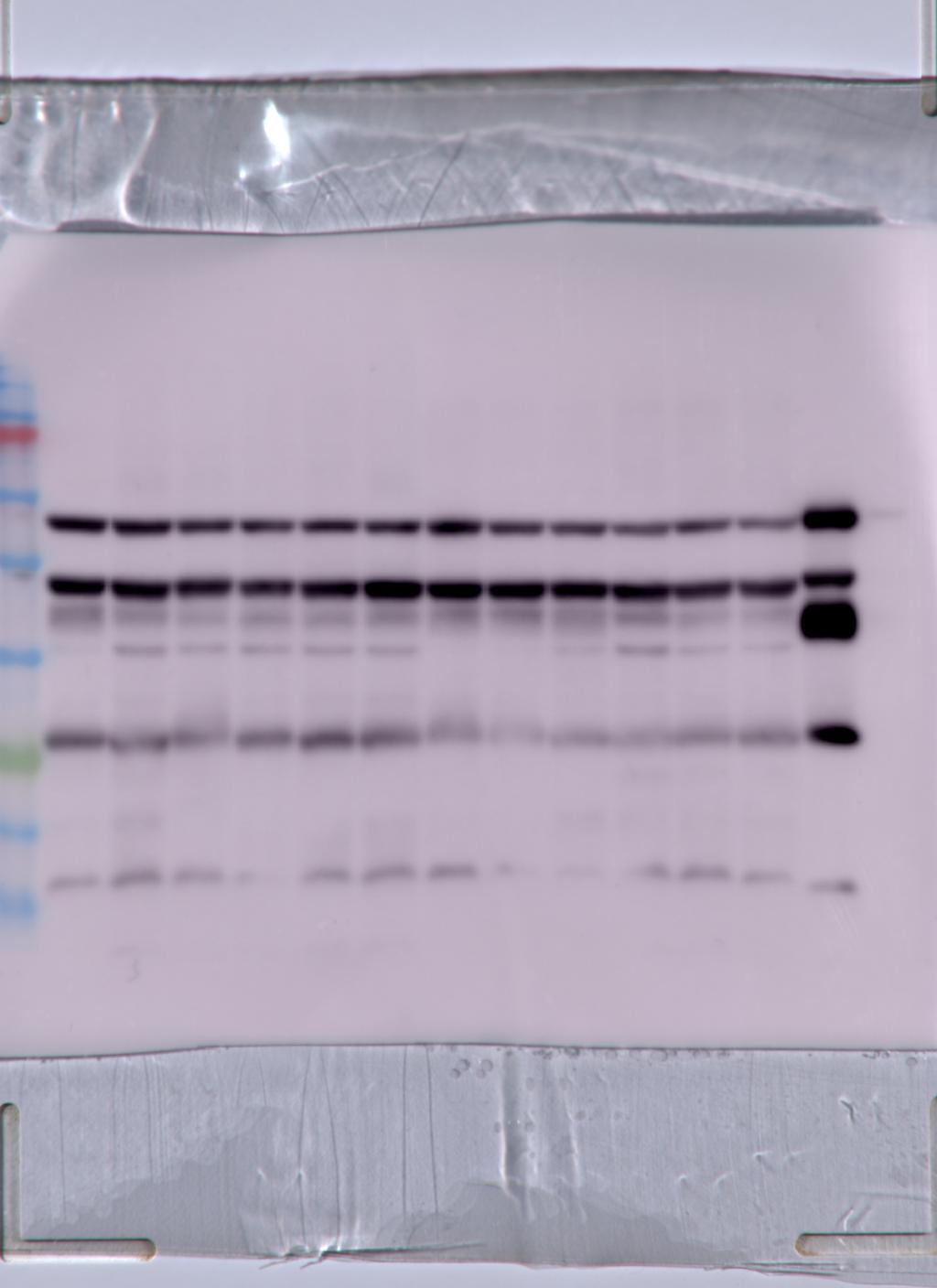

Supplement: Supplementary file 8 — Source data Fig. 6 [file 44318_2025_622_MOESM8_ESM.zip › Figure 6/Fig. 6I/OXPHOS 12% gel 011124 2024.11.01_10.49.42_Ch+Marker.jpg]

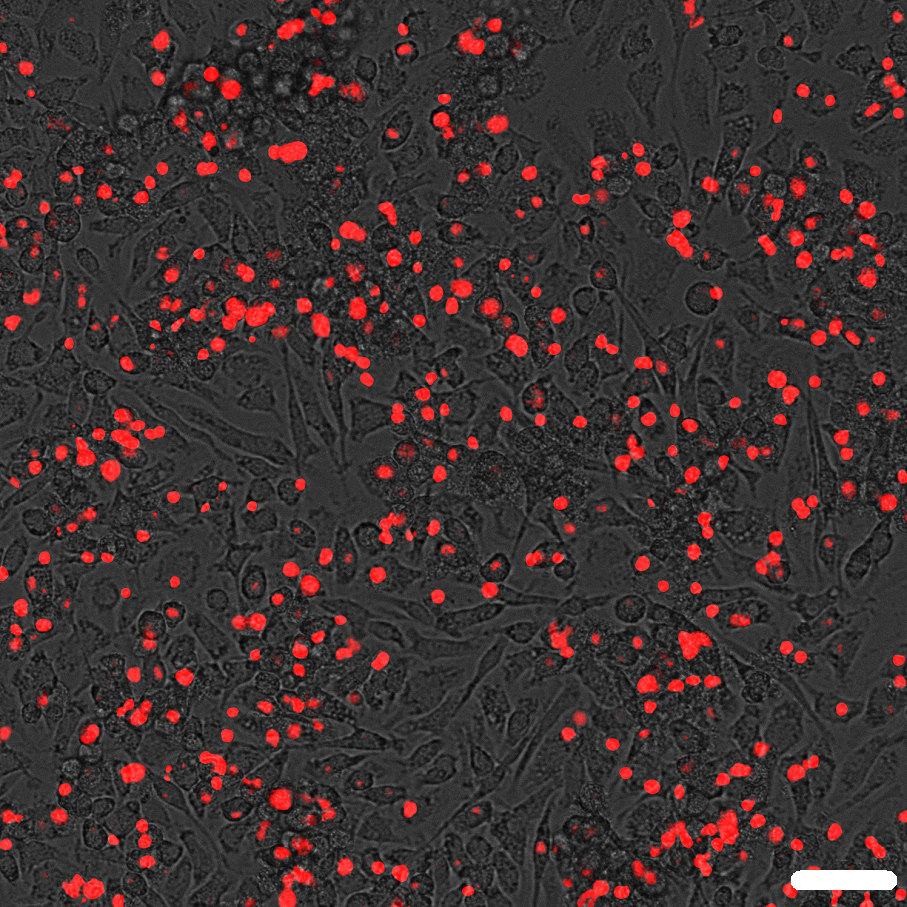

Supplement: Supplementary file 10 — Source data Fig. 8 [file 44318_2025_622_MOESM10_ESM.zip › Figure 8/Fig. 8J/cre mito.png]

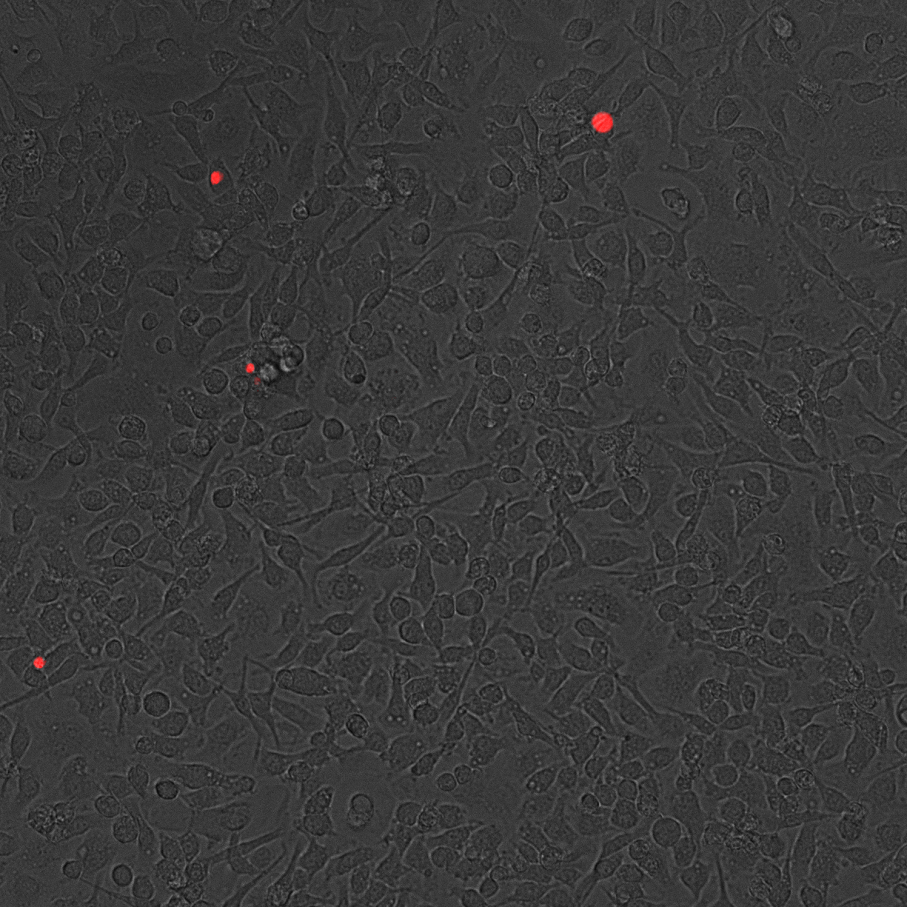

Supplement: Supplementary file 10 — Source data Fig. 8 [file 44318_2025_622_MOESM10_ESM.zip › Figure 8/Fig. 8J/cre vehicle.png]

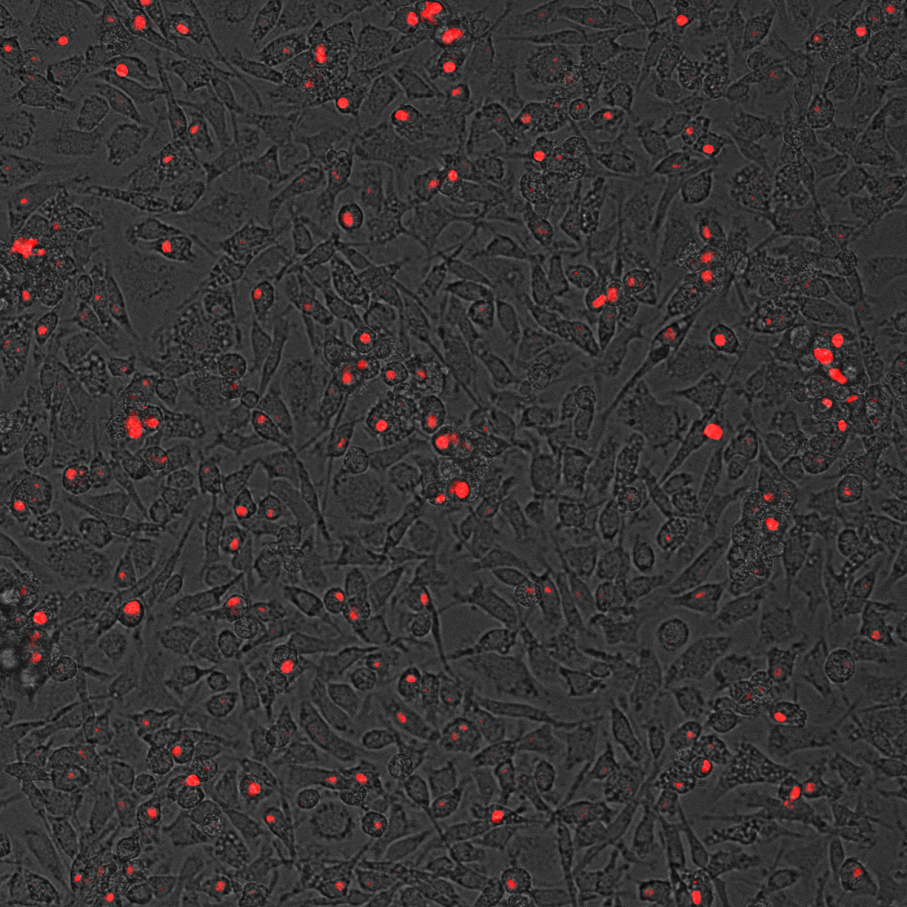

Supplement: Supplementary file 10 — Source data Fig. 8 [file 44318_2025_622_MOESM10_ESM.zip › Figure 8/Fig. 8J/lox mito.png]

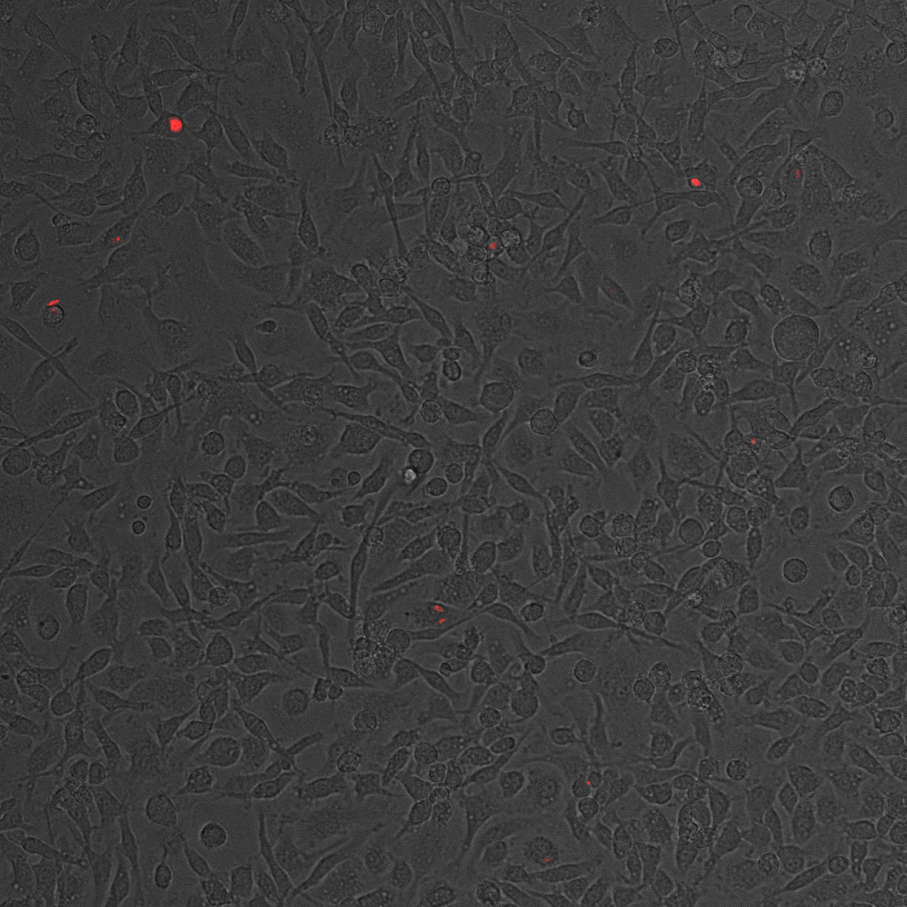

Supplement: Supplementary file 10 — Source data Fig. 8 [file 44318_2025_622_MOESM10_ESM.zip › Figure 8/Fig. 8J/lox vehicle.png]
