# Supplementary material for: Homeostatic control of energy metabolism by monocyte-derived macrophages
Source: EMBO J. 2025 Nov 17;45(1):106–50. doi: 10.1038/s44318-025-00622-x (PMC12759084; doi:10.1038/s44318-025-00622-x)
Supplement: Supplementary file 11 — Expanded View Figures [file 44318_2025_622_MOESM11_ESM.pdf]

Expanded View Figures

Figure EV1. Monocyte-derived macrophages are depleted in chimeric *Fth*-deleted mice.

Schematic representation of TAM-induced *Fth* deletion in chimeric mice (day 0) and representative flow cytometry dot plots for (**A**) monocyte-derived macrophage (backgated as Ly6G^{-}, CD11b^{+}, F4/80^{low}) and (**B**) tissue-resident macrophage (Ly6G^{-}, CD11b$^{-/low}$, F4/80^{high}) populations present in the liver, heart, lungs and kidneys of $Fth^{fl/f}$⇨$Fth^{fl/fl}$ ($n = 7$), $Fth^{R26\Delta/\Delta}$⇨$Fth^{R26\Delta/\Delta}$ ($n = 5$), $Fth^{fl/fl}$⇨$Fth^{R26\Delta/\Delta}$ ($n = 7$) and $Fth^{LysM\Delta/\Delta}$⇨$Fth^{R26\Delta/\Delta}$ ($n = 6$) chimeric mice, 7 to 19 days post-TAM administration. Data in (**A**, **B**) is pooled from 3 independent experiments with similar trends. (**C**) Absolute number of tissue-resident macrophages (Ly6G^{-}CD11b$^{-/low}$F4/80^{high}) in the liver, heart, lungs and kidneys of $Fth^{fl/fl}$⇨$Fth^{fl/fl}$ ($n = 7$), $Fth^{R26\Delta/\Delta}$⇨$Fth^{R26\Delta/\Delta}$ ($n = 5$), $Fth^{fl/fl}$⇨$Fth^{R26\Delta/\Delta}$ ($n = 7$) and $Fth^{LysM\Delta/\Delta}$⇨$Fth^{R26\Delta/\Delta}$ ($n = 6$) chimeric mice, 7 to 19 days post-TAM administration. Data in (**C**) presented as individual values (circles) and mean (red bars), pooled from 3 independent experiments with similar trends. (**D**) Schematic representation of TAM-induced *Fth* deletion (day 0) in chimeric mice, and survival of $Fth^{fl/fl}$⇨$Fth^{fl/fl}$ ($n = 10$), $Fth^{Cx3cr1\Delta/\Delta}$⇨$Fth^{R26\Delta/\Delta}$ ($n = 12$) and $Fth^{fl/fl}$⇨$Fth^{R26\Delta/\Delta}$ ($n = 10$) chimeric mice. Data in (**D**) pooled from 3 independent experiments with similar trends. (**E**) Schematic representation of TAM-induced *Fth* deletion in chimeric mice, and survival of $Ccr2^{+/+}$⇨$Fth^{fl/fl}$ ($n = 7$), $Ccr2^{-/-}$⇨$Fth^{R26\Delta/\Delta}$ ($n = 6$) and $Ccr2^{+/+}$⇨⇨$Fth^{R26\Delta/\Delta}$ ($n = 15$) chimeric mice following TAM administration on day 0. Data in (**E**) is pooled from 3 independent experiments with similar trends. (**F**) Time course of relative body weight changes of $Fth^{fl/fl}$⇔$Fth^{fl/fl}$ ($n = 7$), $Fth^{R26\Delta/\Delta}$⇔$Fth^{R26\Delta/\Delta}$ ($n = 10$), $Fth^{fl/fl}$⇔$Fth^{R26\Delta/\Delta}$ ($n = 14$) and $Fth^{LysM\Delta/\Delta}$⇔$Fth^{R26\Delta/\Delta}$ ($n = 11$) parabiotic mouse pairs, following TAM administration on day 0. Data in (**F**) are presented as mean ± SD, normalized to the initial body weight (t_0) and pooled from 4 independent experiments with similar trends. Survival analysis was performed using Log-rank (Mantel–Cox) test. One-way ANOVA with Tukey's range test for multiple comparison correction was used for comparison between multiple groups. NS: non-significant, *$P < 0.05$, **$P < 0.01$, ***$P < 0.001$.

© The Author(s)

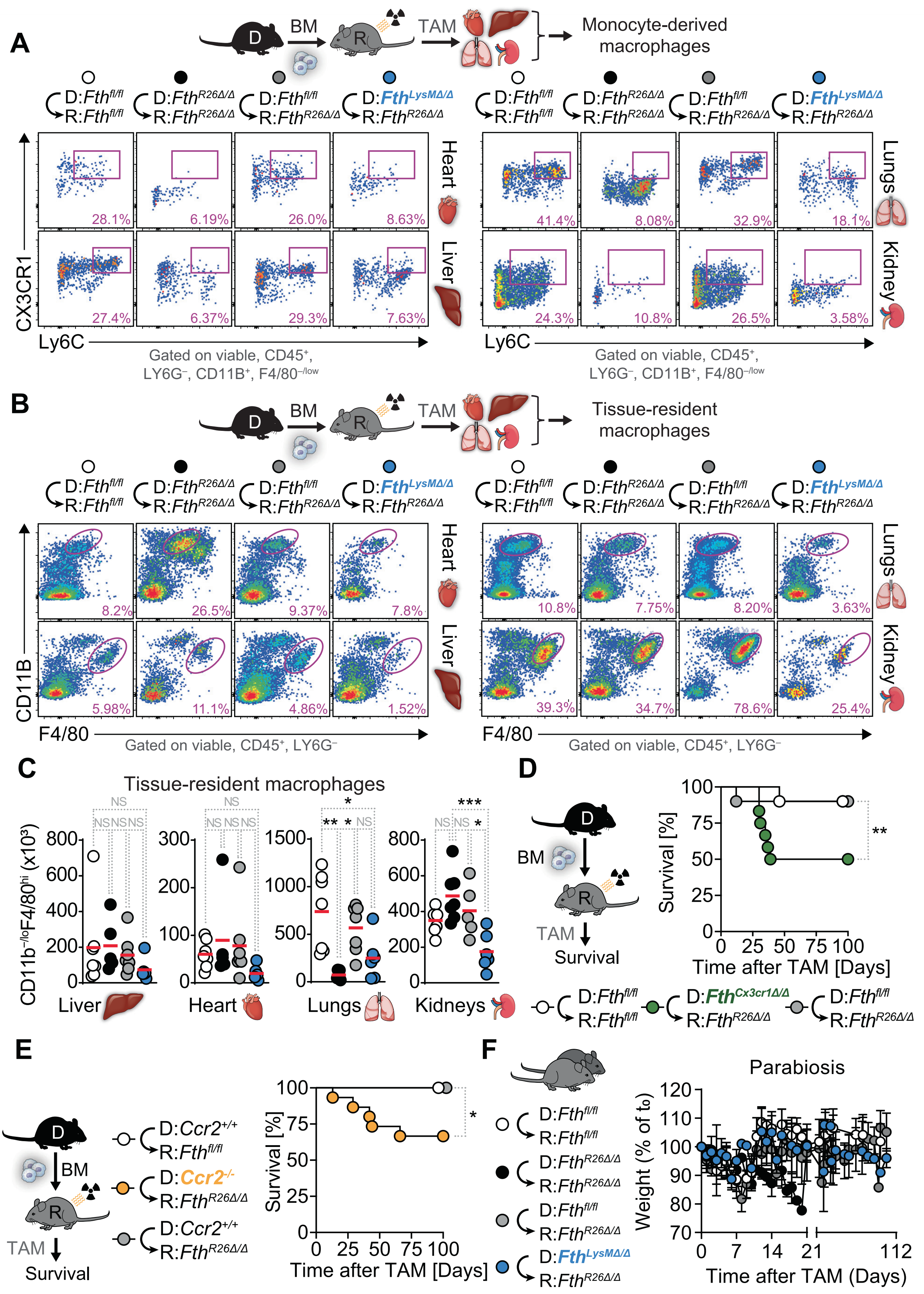
A
BM
TAM
Monocyte-derived macrophages
Heart
Liver
Lungs
Kidney
CX3CR1
Ly6C
Gated on viable, CD45+, LY6G−, CD11B+, F4/80−/low
B
Tissue-resident macrophages
CD11B
F4/80
Gated on viable, CD45+, LY6G−
C
Tissue-resident macrophages
Liver
Heart
Lungs
Kidneys
D
Survival [%]
Time after TAM [Days]
E
F
Parabiosis
Weight (% of t0)
Time after TAM (Days)

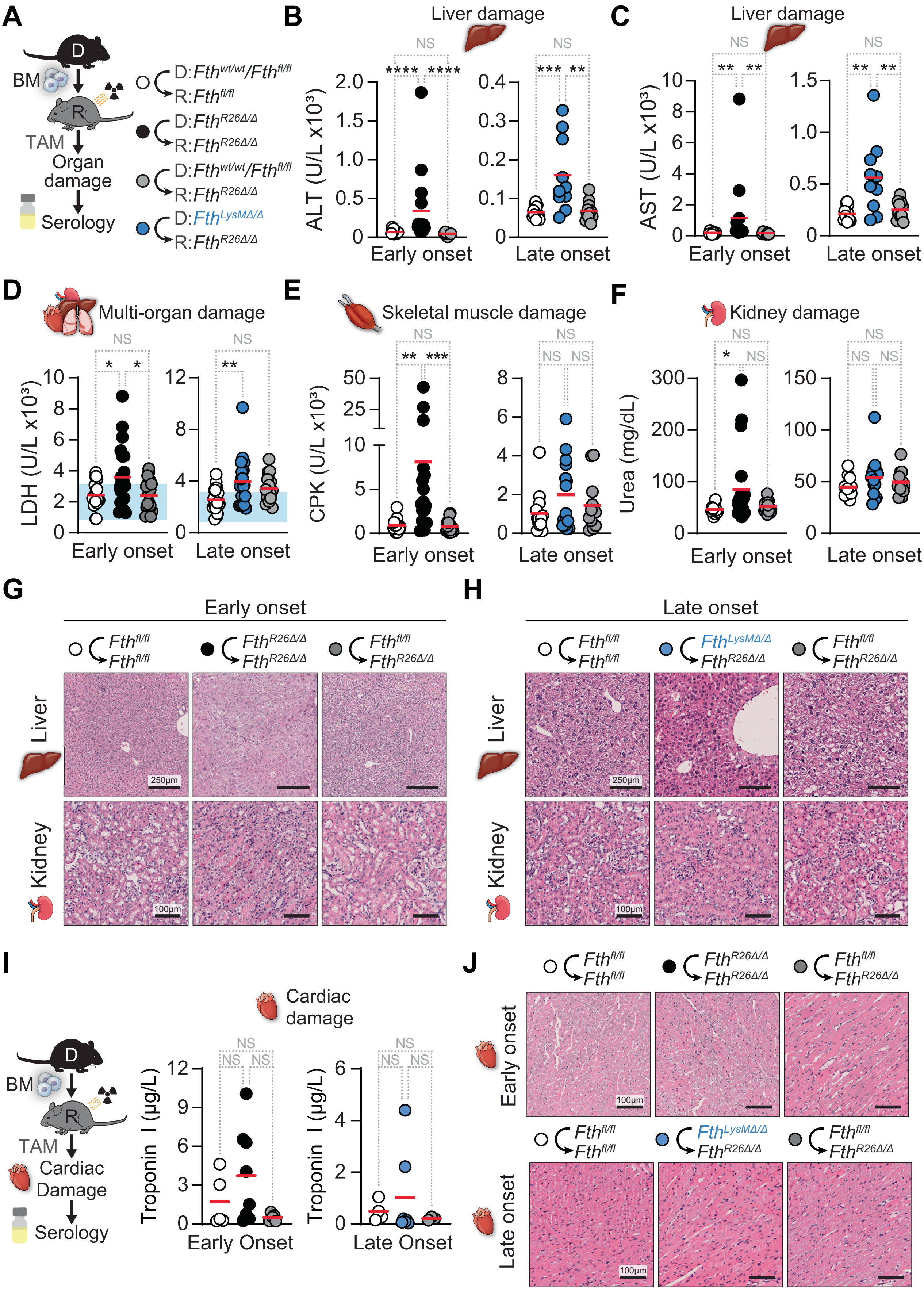
A
D
BM
R
TAM
Organ damage
Serology
D:Fthwt/wt/Fthfl/fl
R:Fthfl/fl
D:FthR26Δ/Δ
R:FthR26Δ/Δ
D:Fthwt/wt/Fthfl/fl
R:FthR26Δ/Δ
D:FthLysMΔ/Δ
R:FthR26Δ/Δ
B
Liver damage
ALT (U/L x10³)
Early onset
Late onset
C
Liver damage
AST (U/L x10³)
Early onset
Late onset
D
Multi-organ damage
LDH (U/L x10³)
Early onset
Late onset
E
Skeletal muscle damage
CPK (U/L x10³)
Early onset
Late onset
F
Kidney damage
Urea (mg/dL)
Early onset
Late onset
G
Early onset
Liver
Kidney
250μm
100μm
H
Late onset
Liver
Kidney
250μm
100μm
I
Cardiac damage
D
BM
R
TAM
Cardiac Damage
Serology
Troponin I (μg/L)
Early Onset
Late Onset
J
Early onset
Late onset
100μm

 © The Author(s)

Figure EV2. *Fth*-competent myeloid cells support tissue function in chimeric *Fth*-deleted mice.

(**A**) Schematic representation of chimeric mice and TAM-induced *Fth* deletion. Plasma levels of (**B**) alanine transaminase (ALT), (**C**) aspartate transaminase (AST), (**D**) lactate dehydrogenase (LDH), (**E**) creatine phosphokinase (CPK) and (**F**) urea, measured in $Fth^{fl/fl}$⇨$Fth^{fl/fl}$ ($n = 11$–21), $Fth^{R26\Delta/\Delta}$⇨$Fth^{R26\Delta/\Delta}$ ($n = 15$–25), $Fth^{fl/fl}$⇨$Fth^{R26\Delta/\Delta}$ ($n = 9$–18) and $Fth^{LysM\Delta/\Delta}$⇨$Fth^{R26\Delta/\Delta}$ ($n = 11$–20) chimeric mice on days 7–15 (early onset), or 19–35 (late onset) following TAM administration. Data in (**B–F**) represented as individual values (circles) and mean (red bars) pooled from 4 to 6 independent experiments with similar trends. (**G**, **H**) Representative hematoxylin and eosin (H&E) stained histology images of liver, kidney and heart from $Fth^{fl/fl}$⇨$Fth^{fl/fl}$, $Fth^{R26\Delta/\Delta}$⇨$Fth^{R26\Delta/\Delta}$, $Fth^{fl/fl}$⇨$Fth^{R26\Delta/\Delta}$ and $Fth^{LysM\Delta/\Delta}$⇨$Fth^{R26\Delta/\Delta}$ chimeric mice on day 7 (early onset) (**G**), or 22 (late onset) (**H**) following TAM administration. One-way ANOVA with Tukey's range test for multiple comparison correction was used for comparison between multiple groups. NS: non-significant, $^{*}P < 0.05$, $^{**}P < 0.01$, $^{***}P < 0.001$, $^{****}P < 0.0001$.

© The Author(s)

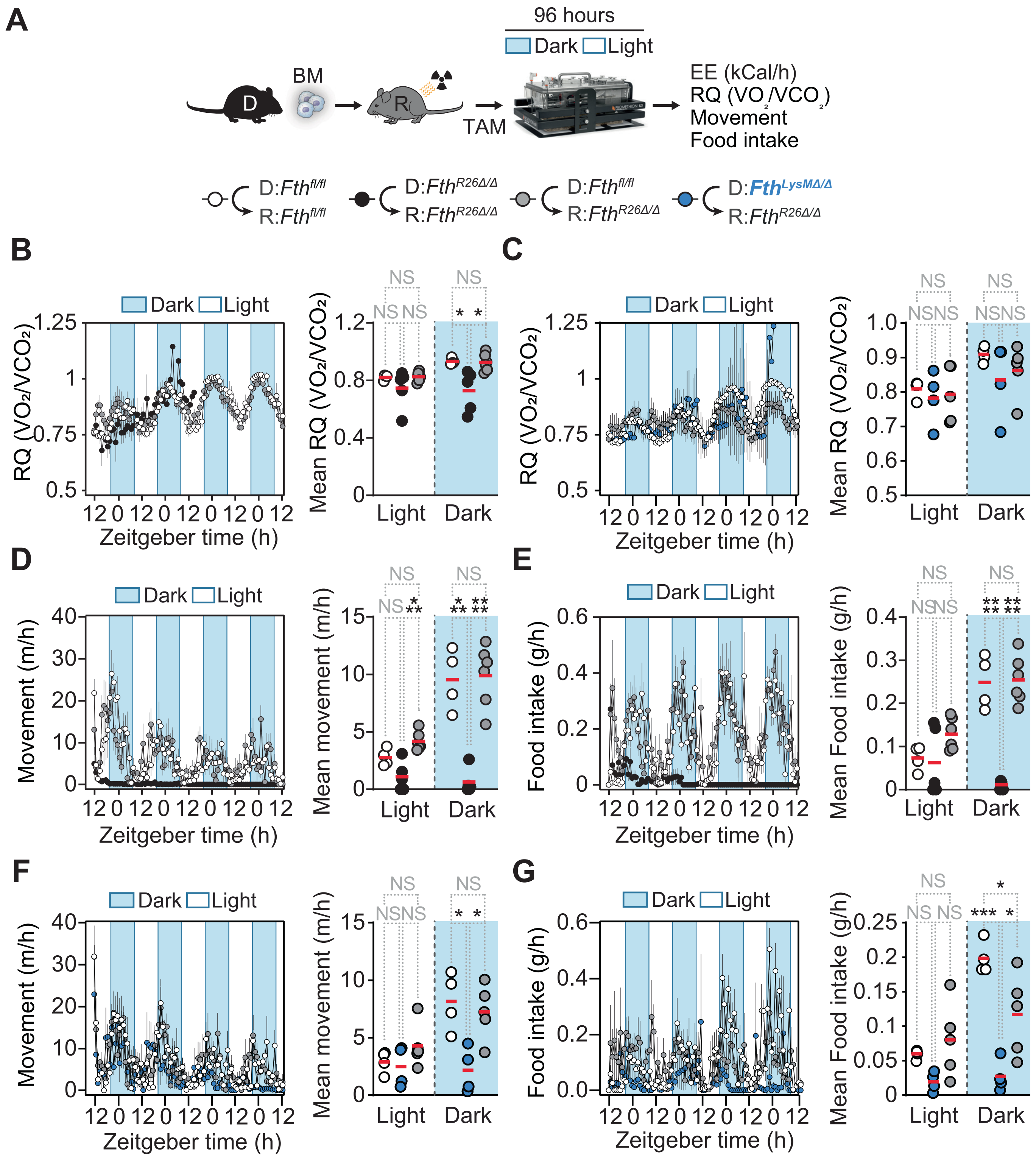
A
96 hours
Dark Light
BM
D
R
TAM
EE (kCal/h)
RQ (VO2/VCO2)
Movement
Food intake
D:Fthfl/fl R:Fthfl/fl
D:FthR26Δ/Δ R:FthR26Δ/Δ
D:Fthfl/fl R:FthR26Δ/Δ
D:FthLysMΔ/Δ R:FthR26Δ/Δ
B
C
D
E
F
G
Dark Light
RQ (VO2/VCO2)
Mean RQ (VO2/VCO2)
Movement (m/h)
Mean movement (m/h)
Food intake (g/h)
Mean Food intake (g/h)
Zeitgeber time (h)
Light
Dark
NS
*
**
***

 © The Author(s)

Figure EV3. *Fth*-competent myeloid cells restore movement and food intake in chimeric *Fth*-deleted mice.

(**A**) Schematic representation of TAM-induced *Fth* deletion in chimeric mice (day 0) and metabolic cage assessment of metabolic parameters. (**B**, **C**) Time course of respiratory quotient (RQ, calculated as VO_2/VCO_2), and mean (red bars) RQ during daytime/nighttime (dot plots) of $Fth^{fl/fl}$⇨$Fth^{fl/fl}$ ($n = 4$), $Fth^{R26\Delta/\Delta}$⇨$Fth^{R26\Delta/\Delta}$ ($n = 5$), $Fth^{fl/fl}$⇨$Fth^{R26\Delta/\Delta}$ ($n = 5$–6) and $Fth^{LysM\Delta/\Delta}$⇨$Fth^{R26\Delta/\Delta}$ ($n = 4$) chimeric mice, assessed from day 7 (**B**; early onset), or day 20 (**C**; late onset) post TAM administration. Time course and mean (red bars) of daytime/nighttime values (dot plots) for mouse movement (m/h; **D**), and rate of food intake (g/h; **E**) of $Fth^{fl/fl}$⇨$Fth^{fl/fl}$ ($n = 4$), $Fth^{R26\Delta/\Delta}$⇨$Fth^{R26\Delta/\Delta}$ ($n = 5$) and $Fth^{fl/fl}$⇨$Fth^{R26\Delta/\Delta}$ ($n = 6$) chimeric mice, assessed from day 7 (early onset). Time course and mean (red bars) of daytime/nighttime values (dot plots) for mouse movement (m/h; **F**), and rate of food intake (g/h; **G**) of $Fth^{fl/fl}$⇨$Fth^{fl/fl}$ ($n = 4$), $Fth^{LysM\Delta/\Delta}$⇨$Fth^{R26\Delta/\Delta}$ ($n = 4$) and $Fth^{fl/fl}$⇨$Fth^{R26\Delta/\Delta}$ ($n = 5$) chimeric mice, assessed from day 20 (late onset). Data in (**B**–**G**) is displayed as mean ± SD (time course), or as individual values (circles) and mean (red bars) (dot plots). Data in (**B**–**G**) is pooled from 2 independent experiments with similar trend. One-way ANOVA with Tukey's range test for multiple comparison correction was used for comparison between multiple groups. NS: non-significant, $^*P < 0.05$, $^{***}P < 0.001$, $^{****}P < 0.0001$.

© The Author(s)

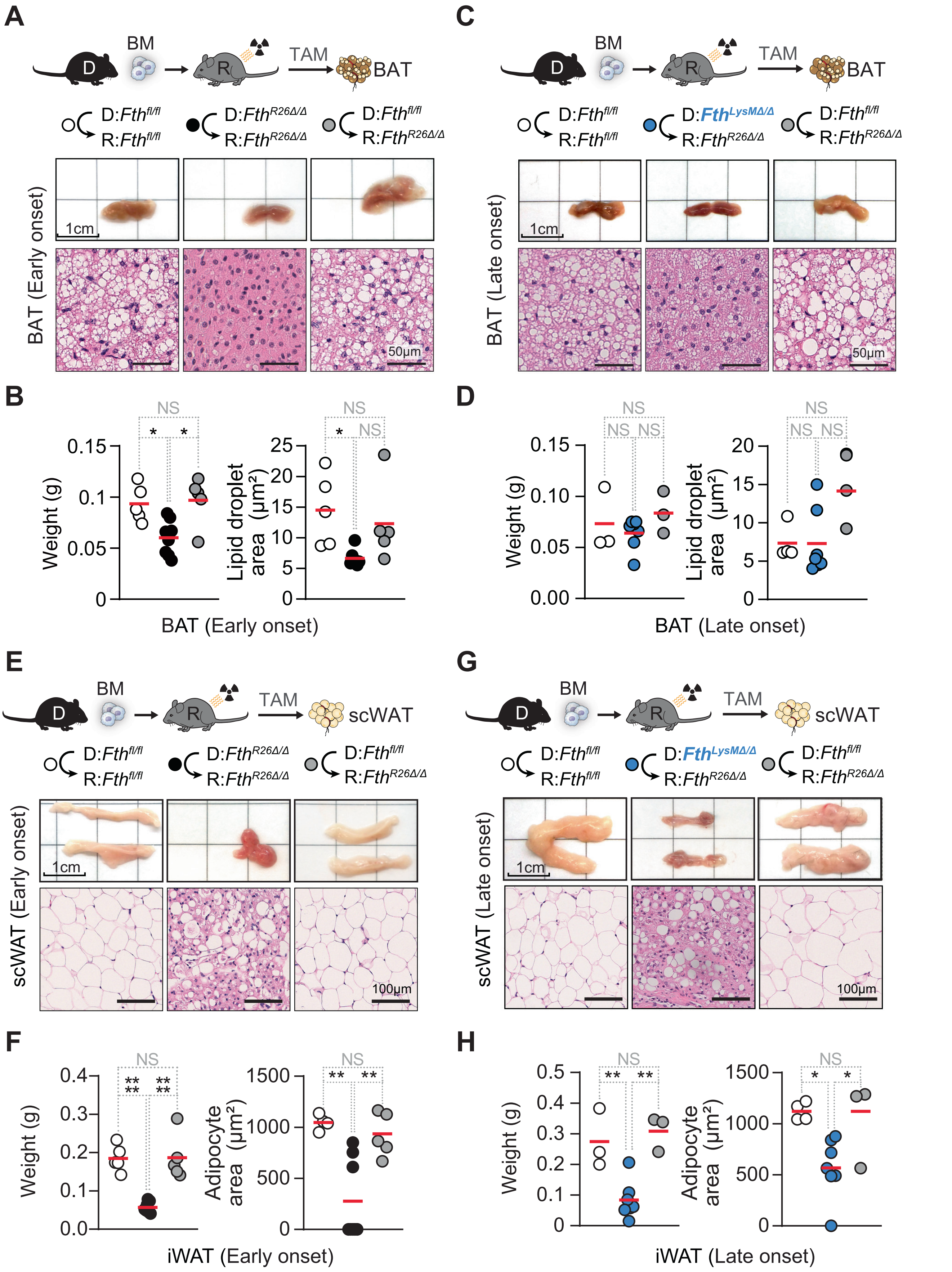

A
BM
D
R
TAM
BAT
D:Fth fl/fl R:Fth fl/fl
D:Fth R26Δ/Δ R:Fth R26Δ/Δ
D:Fth fl/fl R:Fth R26Δ/Δ
BAT (Early onset)
1cm
50µm
C
BAT (Late onset)
D:Fth LysMΔ/Δ R:Fth R26Δ/Δ
B
Weight (g)
Lipid droplet area (µm²)
NS
BAT (Early onset)
D
BAT (Late onset)
E
scWAT
scWAT (Early onset)
100µm
G
scWAT (Late onset)
F
Adipocyte area (µm²)
iWAT (Early onset)
H
iWAT (Late onset)

 © The Author(s)

Figure EV4. *Fth*-competent myeloid cells support BAT and WAT function in chimeric *Fth*-deleted mice.

(**A**) Schematic representation of chimeric mice and TAM-induced *Fth* deletion, and representative macroscopic and histological images of brown adipose tissue pads (BAT) of $Fth^{fl/fl}$⇨$Fth^{fl/fl}$ ($n = 5$), $Fth^{R26\Delta/\Delta}$⇨$Fth^{R26\Delta/\Delta}$ ($n = 8$) and $Fth^{fl/fl}$⇨$Fth^{R26\Delta/\Delta}$ ($n = 5$) chimeric mice, collected on day 7 (early onset) post TAM administration. (**B**) BAT pad weight (left) and mean (red bars) BAT adipocyte lipid droplet area (right) of $Fth^{fl/fl}$⇨$Fth^{fl/fl}$ ($n = 5$), $Fth^{R26\Delta/\Delta}$⇨$Fth^{R26\Delta/\Delta}$ ($n = 8$) and $Fth^{fl/fl}$⇨$Fth^{R26\Delta/\Delta}$ ($n = 5$) chimeric mice, collected on day 7 (early onset) post TAM administration. (**C**) Schematic representation of chimeric mice and TAM-induced *Fth* deletion, and representative macroscopic and histological images of BAT of $Fth^{fl/fl}$⇨$Fth^{fl/fl}$ ($n = 3$–4), $Fth^{LysM\Delta/\Delta}$⇨$Fth^{R26\Delta/\Delta}$ ($n = 6$) and $Fth^{fl/fl}$⇨$Fth^{R26\Delta/\Delta}$ ($n = 3$–4) chimeric mice, collected between days 16 and 39 (late onset) post TAM administration. (**D**) BAT pad weight (left) and mean (red bars) BAT adipocyte lipid droplet area (right) of $Fth^{fl/fl}$⇨$Fth^{fl/fl}$ ($n = 3$–4), $Fth^{LysM\Delta/\Delta}$⇨$Fth^{R26\Delta/\Delta}$ ($n = 6$) and $Fth^{fl/fl}$⇨$Fth^{R26\Delta/\Delta}$ ($n = 3$–4) chimeric mice, collected between days 16 and 39 (late onset) post TAM administration. Data in (**B**, **D**) represented as individual values (circles) and mean (red bars), pooled from three independent experiments with similar trends. (**E**) Schematic representation of chimeric mice and TAM-induced *Fth* deletion, and representative macroscopic and histological images of inguinal white adipose tissue pads (iWAT) of $Fth^{fl/fl}$⇨$Fth^{fl/fl}$ ($n = 4$–5), $Fth^{R26\Delta/\Delta}$⇨$Fth^{R26\Delta/\Delta}$ ($n = 8$) and $Fth^{fl/fl}$⇨$Fth^{R26\Delta/\Delta}$ ($n = 5$) chimeric mice, collected on day 7 (early onset) post TAM administration. (**F**) iWAT pad weight (left) and mean (red bars) iWAT adipocyte area (right) of $Fth^{fl/fl}$⇨$Fth^{fl/fl}$ ($n = 4$–5), $Fth^{R26\Delta/\Delta}$⇨$Fth^{R26\Delta/\Delta}$ ($n = 8$) and $Fth^{fl/fl}$⇨$Fth^{R26\Delta/\Delta}$ ($n = 5$) chimeric mice, collected on day 7 (early onset) post TAM administration. (**G**) Schematic representation of chimeric mice and TAM-induced *Fth* deletion, and representative macroscopic and histological images of iWAT of $Fth^{fl/fl}$⇨$Fth^{fl/fl}$ ($n = 3$–4), $Fth^{LysM\Delta/\Delta}$⇨$Fth^{R26\Delta/\Delta}$ ($n = 7$) and $Fth^{fl/fl}$⇨$Fth^{R26\Delta/\Delta}$ ($n = 3$) chimeric mice, collected between days 16 and 39 (late onset) post TAM administration. (**H**) iWAT pad weight (left) and mean (red bars) iWAT adipocyte area (right) of $Fth^{fl/fl}$⇨$Fth^{fl/fl}$ ($n = 3$–4), $Fth^{LysM\Delta/\Delta}$⇨$Fth^{R26\Delta/\Delta}$ ($n = 7$) and $Fth^{fl/fl}$⇨$Fth^{R26\Delta/\Delta}$ ($n = 3$) chimeric mice, collected between days 16 and 39 (late onset) post TAM administration. Data in (**F**, **H**) represented as individual values (circles) and mean (red bars), pooled from 3 independent experiments with similar trends. One-way ANOVA with Tukey's range test for multiple comparison correction was used for comparison between multiple groups. NS: non-significant, *$P < 0.05$, **$P < 0.01$, ****$P < 0.0001$.

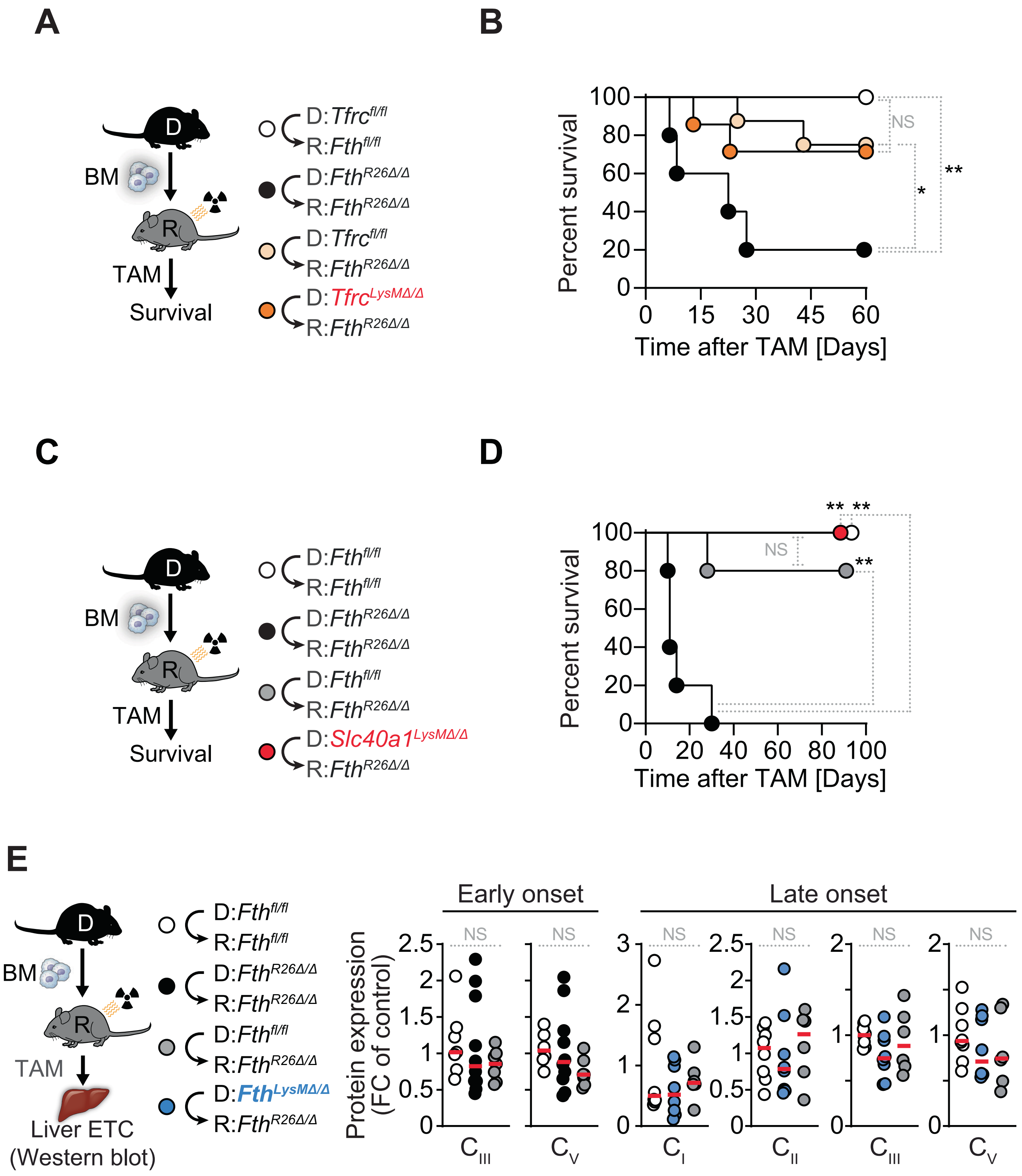
A
D
BM
R
TAM
Survival
D:Tfrc fl/fl
R:Fth fl/fl
D:Fth R26Δ/Δ
R:Fth R26Δ/Δ
D:Tfrc fl/fl
R:Fth R26Δ/Δ
D:Tfrc LysMΔ/Δ
R:Fth R26Δ/Δ
B
Percent survival
Time after TAM [Days]
NS
*
**
C
D:Fth fl/fl
R:Fth fl/fl
D:Fth R26Δ/Δ
R:Fth R26Δ/Δ
D:Fth fl/fl
R:Fth R26Δ/Δ
D:Slc40a1 LysMΔ/Δ
R:Fth R26Δ/Δ
D
Percent survival
Time after TAM [Days]
E
Liver ETC
(Western blot)
D:Fth LysMΔ/Δ
Early onset
Late onset
Protein expression
(FC of control)
NS
CIII
CV
CI
CII

 © The Author(s)

Figure EV5. Myeloid cells rescue chimeric *Fth*-deleted mice irrespectively of cellular Fe import/export.

(**A**) Schematic representation of TAM-induced *Fth* deletion in chimeric mice, and (**B**) survival of *Tfrc*$^{fl/fl}$⇨*Fth*$^{fl/fl}$ ($n = 7$), *Fth*$^{R26\Delta/\Delta}$⇨*Fth*$^{R26\Delta/\Delta}$ ($n = 5$), *Tfrc*$^{fl/fl}$⇨*Fth*$^{R26\Delta/\Delta}$ ($n = 8$) and *Tfrc*$^{LysM\Delta/\Delta}$⇨*Fth*$^{R26\Delta/\Delta}$ ($n = 7$) chimeric mice following TAM administration. Data in (**B**) is pooled from 2 independent experiments with similar trends. (**C**) Schematic representation of TAM-induced *Fth* deletion in chimeric mice, and (**D**) survival of *Fth*$^{fl/fl}$⇨*Fth*$^{fl/fl}$ ($n = 5$), *Fth*$^{R26\Delta/\Delta}$⇨*Fth*$^{R26\Delta/\Delta}$ ($n = 5$), *Fth*$^{fl/fl}$⇨*Fth*$^{R26\Delta/\Delta}$ ($n = 5$), and *Slc40a1*$^{LysM\Delta/\Delta}$⇨*Fth*$^{R26\Delta/\Delta}$ ($n = 5$) chimeric mice on day 0. (**E**) quantification of ETC subunits for complexes I-V in the livers of *Fth*$^{fl/fl}$⇨*Fth*$^{fl/fl}$ ($n = 7$), *Fth*$^{R26\Delta/\Delta}$⇨*Fth*$^{R26\Delta/\Delta}$ ($n = 10$) and *Fth*$^{fl/fl}$⇨*Fth*$^{R26\Delta/\Delta}$ ($n = 7$) chimeric mice, between days 7–8 (early onset), or from *Fth*$^{fl/fl}$⇨*Fth*$^{fl/fl}$ ($n = 8$), *Fth*$^{LysM\Delta/\Delta}$⇨*Fth*$^{R26\Delta/\Delta}$ ($n = 8$) and *Fth*$^{fl/fl}$⇨*Fth*$^{R26\Delta/\Delta}$ ($n = 6$) chimeric mice, between days 19 and 22 (late onset). Data in (**E**) is pooled from 4 independent experiments with similar trends. One-way ANOVA with Tukey's range test for multiple comparison correction was used for comparison between multiple groups. Survival analysis was performed using Log-rank (Mantel–Cox) test. NS: non-significant, $^{*}P < 0.05$, $^{**}P < 0.01$.

© The Author(s)

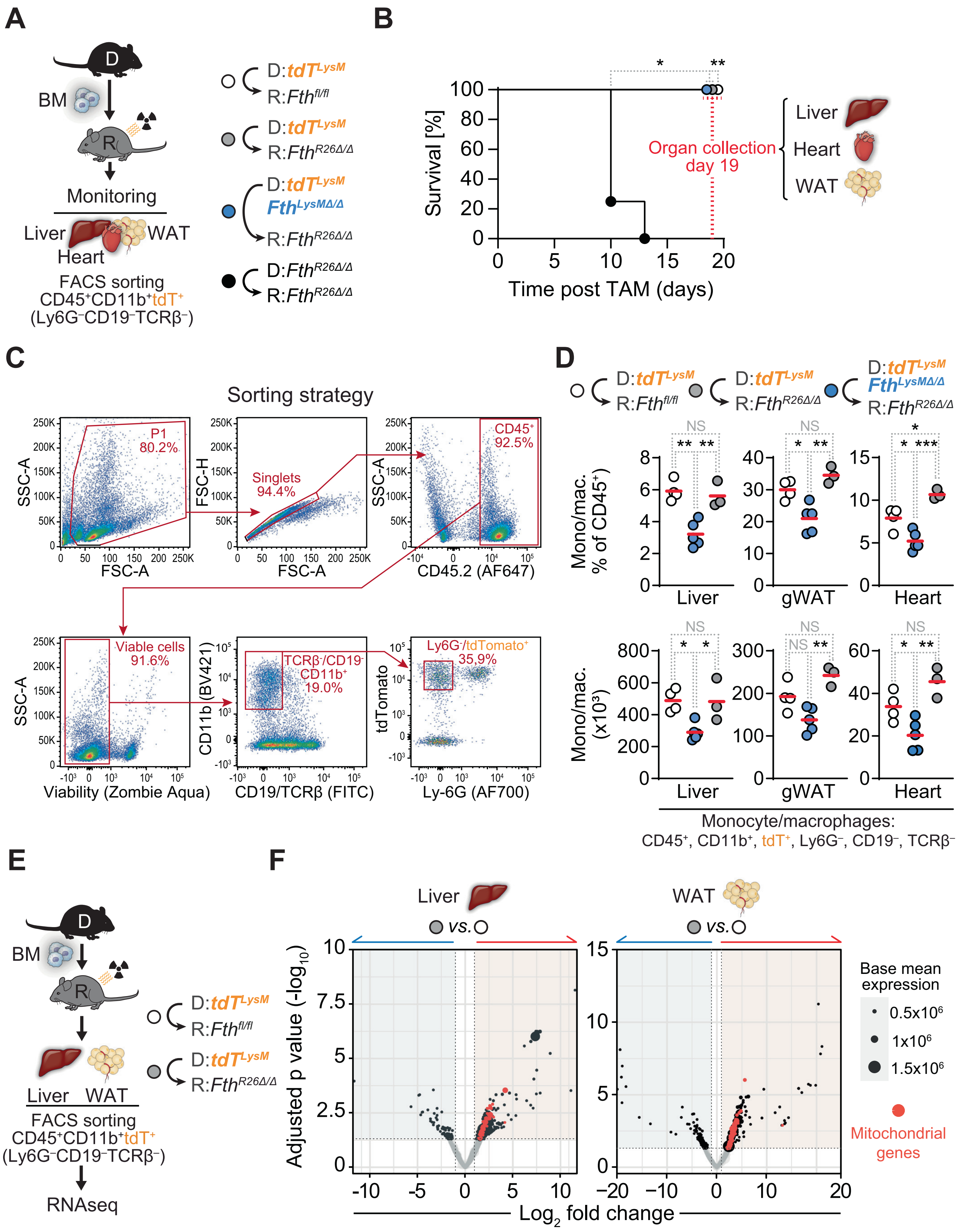
A
D
BM
R
Monitoring
Liver
WAT
Heart
FACS sorting
CD45⁺CD11b⁺tdT⁺
(Ly6G⁻CD19⁻TCRβ⁻)
D:tdT^LysM
R:Fth^fl/fl
D:tdT^LysM
R:Fth^R26Δ/Δ
D:tdT^LysM
Fth^LysMΔ/Δ
R:Fth^R26Δ/Δ
D:Fth^R26Δ/Δ
R:Fth^R26Δ/Δ
B
Survival [%]
Time post TAM (days)
Organ collection
day 19
Liver
Heart
WAT
C
Sorting strategy
SSC-A
FSC-A
P1
80.2%
FSC-H
Singlets
94.4%
CD45⁺
92.5%
CD45.2 (AF647)
Viable cells
91.6%
Viability (Zombie Aqua)
CD11b (BV421)
TCRβ⁻/CD19⁻
CD11b⁺
19.0%
CD19/TCRβ (FITC)
tdTomato
Ly6G⁻/tdTomato⁺
35.9%
Ly-6G (AF700)
D
Mono/mac.
% of CD45⁺)
Mono/mac.
(x10³)
Liver
gWAT
Heart
NS
Monocyte/macrophages:
CD45⁺, CD11b⁺, tdT⁺, Ly6G⁻, CD19⁻, TCRβ⁻
E
D
BM
R
Liver WAT
FACS sorting
CD45⁺CD11b⁺tdT⁺
(Ly6G⁻CD19⁻TCRβ⁻)
RNAseq
D:tdT^LysM
R:Fth^fl/fl
D:tdT^LysM
R:Fth^R26Δ/Δ
F
Liver
WAT
vs.
Adjusted p value (-log10)
Log2 fold change
Base mean
expression
0.5x10⁶
1x10⁶
1.5x10⁶
Mitochondrial
genes

© The Author(s)

Figure EV6. RNA sequencing analysis of monocyte/macrophages in chimeric *Fth*-deleted mice.

(**A**) Schematic representation of chimeric mice, TAM administration and fluorescence-activated cell sorting (FACS) of *LysM*$^+$ monocyte/macrophages (CD45$^+$,CD11b$^+$,Ly6G$^-$,CD19$^-$,TCRβ$^-$) in liver, heart and WAT. (**B**) Survival of *tdT*^{LysM}⇨*Fth*$^{fl/fl}$ ($n = 4$), *tdT*^{LysM}⇨*Fth*$^{R26\Delta/\Delta}$ ($n = 3$), *tdT*^{LysM}*Fth*$^{LysM\Delta/\Delta}$⇨*Fth*$^{R26\Delta/\Delta}$ ($n = 5$) and *Fth*$^{R26\Delta/\Delta}$⇨*Fth*$^{R26\Delta/\Delta}$ ($n = 4$) chimeric mice until organ collection on day 19 post-TAM administration. (**C**) Gating strategy for sorting *LysM*$^+$ monocyte/macrophages (CD45$^+$, CD11b$^+$, Ly6G$^-$, CD19$^-$, TCRβ$^-$) in liver, WAT and Heart. (**D**) Proportion and number and of viable *LysM*$^+$ monocyte/macrophages (CD45$^+$, CD11b$^+$, Ly6G$^-$, CD19$^-$, TCRβ$^-$) in the liver, gWAT and heart of *tdT*^{LysM}⇨*Fth*$^{fl/fl}$ ($n = 4$), *tdT*^{LysM}⇨*Fth*$^{R26\Delta/\Delta}$ ($n = 3$) and *tdT*^{LysM}*Fth*$^{LysM\Delta/\Delta}$⇨*Fth*$^{R26\Delta/\Delta}$ ($n = 5$) chimeric mice on day 19 post-TAM administration. (**E**) Schematic representation of chimeric mice, TAM administration and fluorescence-activated cell sorting (FACS) of *LysM*$^+$ monocyte/macrophages (CD45$^+$, CD11b$^+$, Ly6G$^-$, CD19$^-$, TCRβ$^-$) in liver and WAT. (**F**) Volcano plots of differentially regulated genes between *LysM*$^+$ monocyte/macrophages sorted from liver (left) or WAT (right) of *tdT*^{LysM}⇨*Fth*$^{R26\Delta/\Delta}$ ($n = 3$), *tdT*^{LysM}⇨*Fth*$^{fl/fl}$ ($n = 4$) chimeric mice, on day 19 post TAM administration. Red dots depict mitochondrial genes that are significantly differentially regulated. One-way ANOVA with Tukey's range test for multiple comparison correction was used for comparison between multiple groups. Survival analysis was performed using Log-rank (Mantel–Cox) test. NS: non-significant, *$P < 0.05$, **$P < 0.01$, ***$P < 0.001$.

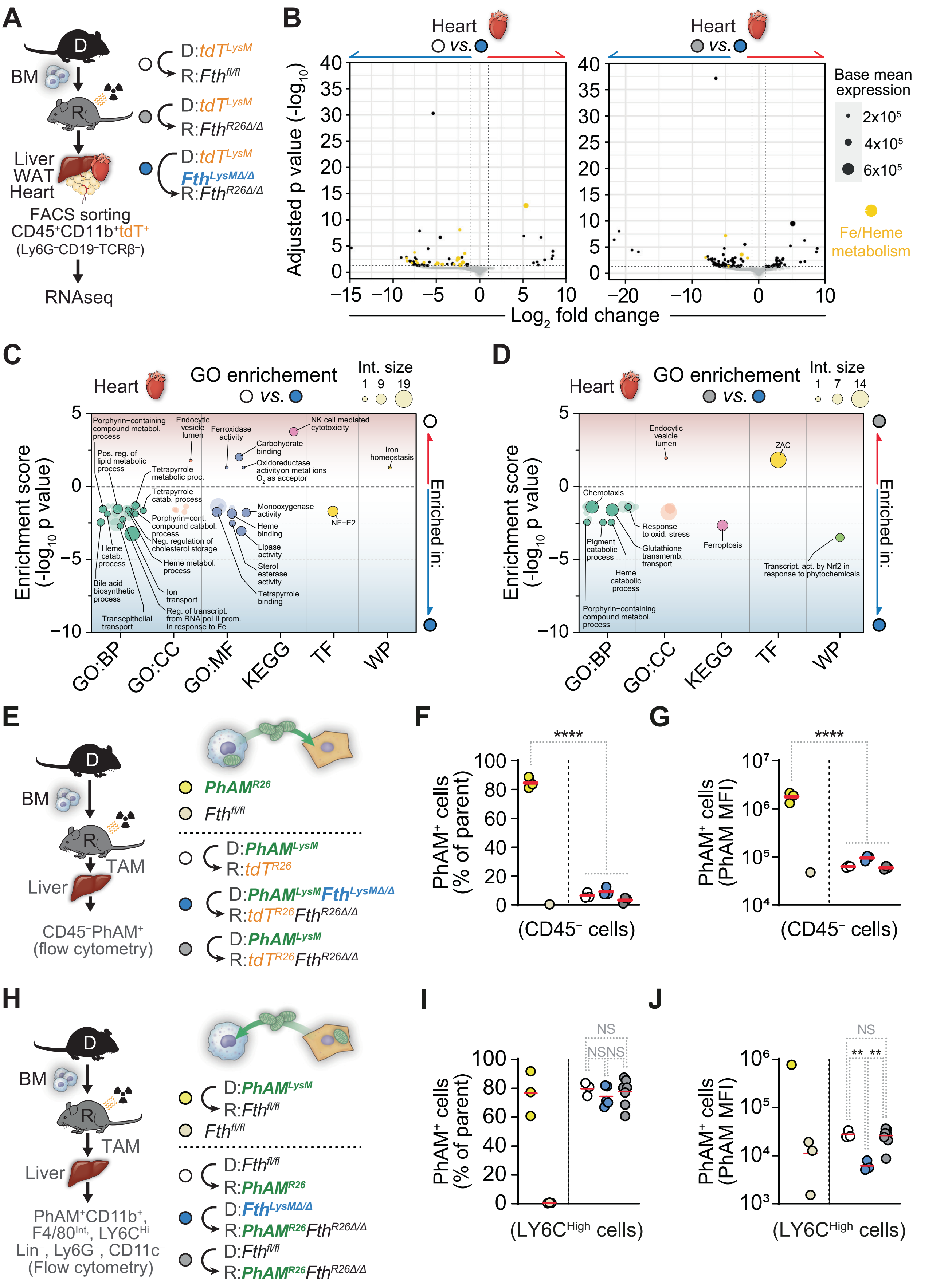
A
D
BM
R
Liver
WAT
Heart
FACS sorting
CD45+CD11b+tdT+
(Ly6G-CD19-TCRβ-)
RNAseq
D:tdTLysM
R:Fthfl/fl
D:tdTLysM
R:FthR26Δ/Δ
D:tdTLysM
FthLysMΔ/Δ
R:FthR26Δ/Δ
B
Heart
vs.
Adjusted p value (-log10)
Log2 fold change
Base mean expression
2x105
4x105
6x105
Fe/Heme metabolism
C
Heart
GO enrichement
vs.
Int. size
1 9 19
Enrichment score (-log10 p value)
Enriched in:
Porphyrin-containing compound metabol. process
Endocytic vesicle lumen
Ferroxidase activity
NK cell mediated cytotoxicity
Carbohydrate binding
Oxidoreductase activityon metal ions O2 as acceptor
Iron homeostasis
Pos. reg. of lipid metabolic process
Tetrapyrrole metabolic proc.
Tetrapyrrole catab. process
Porphyrin-cont. compound catabol. process
Neg. regulation of cholesterol storage
Heme metabol. process
Heme catab. process
Bile acid biosynthetic process
Ion transport
Transepithelial transport
Reg. of transcript. from RNA pol II prom. in response to Fe
Monooxygenase activity
Heme binding
Lipase activity
Sterol esterase activity
Tetrapyrrole binding
NF-E2
GO:BP
GO:CC
GO:MF
KEGG
TF
WP
D
Heart
GO enrichement
vs.
Int. size
1 7 14
Enrichment score (-log10 p value)
Enriched in:
Endocytic vesicle lumen
ZAC
Chemotaxis
Response to oxid. stress
Pigment catabolic process
Glutathione transmemb. transport
Heme catabolic process
Ferroptosis
Transcript. act. by Nrf2 in response to phytochemicals
Porphyrin-containing compound metabol. process
GO:BP
GO:CC
KEGG
TF
WP
E
D
BM
R
TAM
Liver
CD45-PhAM+
(flow cytometry)
PhAMR26
Fthfl/fl
D:PhAMLysM
R:tdTR26
D:PhAMLysM FthLysMΔ/Δ
R:tdTR26 FthR26Δ/Δ
D:PhAMLysM
R:tdTR26 FthR26Δ/Δ
F
****
PhAM+ cells (% of parent)
(CD45- cells)
G
****
PhAM+ cells (PhAM MFI)
(CD45- cells)
H
D
BM
R
TAM
Liver
PhAM+CD11b+, F4/80Int, LY6CHi
Lin-, Ly6G-, CD11c-
(Flow cytometry)
D:PhAMLysM
R:Fthfl/fl
Fthfl/fl
D:Fthfl/fl
R:PhAMR26
D:FthLysMΔ/Δ
R:PhAMR26 FthR26Δ/Δ
D:Fthfl/fl
R:PhAMR26 FthR26Δ/Δ
I
NS
NS NS
PhAM+ cells (% of parent)
(LY6CHigh cells)
J
NS
** **
PhAM+ cells (PhAM MFI)
(LY6CHigh cells)

 © The Author(s)

Figure EV7. *Fth*-competent monocyte-derived macrophages in the heart of *Fth*-deleted chimeras do not employ a mitochondrial gene transcriptional program.

(**A**) Schematic representation of chimeric mice, TAM administration and fluorescence-activated cell sorting (FACS) of $LysM^+$ monocyte/macrophages ($CD45^+$, $CD11b^+$, $Ly6G^-$, $CD19^-$, $TCR\beta^-$) in liver, WAT and heart. (**B**) Volcano plots of differentially regulated genes between $LysM^+$ monocyte/macrophages sorted from the heart of $tdT^{LysM}Fth^{LysM\Delta/\Delta}$⇨$Fth^{R26\Delta/\Delta}$ ($n = 5$) vs. tdT^{LysM}⇨$Fth^{fl/fl}$ ($n = 4$; left) or tdT^{LysM}⇨$Fth^{R26\Delta/\Delta}$ ($n = 3$; right) chimeric mice, on day 19 post TAM administration. Yellow dots depict genes involved in iron/heme metabolism that are significantly differentially regulated. Gene ontology analysis depicting ontologies that are significantly enriched comparing $LysM^+$ monocyte/macrophages sorted from heart of (**C**) $tdT^{LysM}Fth^{LysM\Delta/\Delta}$⇨$Fth^{R26\Delta/\Delta}$ ($n = 5$) chimeras, vs. tdT^{LysM}⇨$Fth^{fl/fl}$ ($n = 4$; left) or (**D**) tdT^{LysM}⇨$Fth^{R26\Delta/\Delta}$ ($n = 3$; right) chimeric mice on day 19 post-TAM administration. Ontologies significantly enriched in $LysM^+$ monocyte/macrophages from tdT^{LysM}⇨$Fth^{fl/fl}$ are depicted as: enrichment score $-\log_{10}$ P value > 1.301. Ontologies significantly enriched in $LysM^+$ monocyte/macrophages from $tdT^{LysM}Fth^{LysM\Delta/\Delta}$⇨$Fth^{R26\Delta/\Delta}$ or tdT^{LysM}⇨$Fth^{R26\Delta/\Delta}$ chimeras are depicted as: enrichment score $-\log_{10}$ P value < -1.301. Ontology classes: TF = transcription factors; GO:CC = gene ontology: cellular component; GO:MF = GO: molecular function; GO:BP = GO: biological process; KEGG = Kyoto Encyclopedia of Genes and Genomes pathway; WP = Wiki Pathways. (**E**) Schematic representation of chimeric mice, TAM administration (day 0), and flow cytometry analysis of livers from positive ($PhAM^{R26}$; $n = 3$) and negative control ($Fth^{fl/fl}$; $n = 1$) mice, and $PhAM^{LysM}$⇨tdT^{R26} ($n = 3$), $PhAM^{LysM}Fth^{LysM\Delta/\Delta}$⇨$tdT^{R26}Fth^{R26\Delta/\Delta}$ ($n = 3$), $PhAM^{LysM}$⇨$tdT^{R26}Fth^{R26\Delta/\Delta}$ ($n = 3$) chimeras. (**F**) Percentage and (**G**) median fluorescence intensity (MFI) of $CD45^-$ cells that are $PhAM^+$. (**H**) Schematic representation of chimeric mice, TAM administration (day 0), and flow cytometry analysis of livers from positive ($PhAM^{LysM}$⇨$Fth^{fl/fl}$; $n = 3$) and negative control ($Fth^{fl/fl}$; $n = 3$) mice, and $Fth^{fl/fl}$⇨$PhAM^{R26}$ ($n = 3$), $Fth^{LysM\Delta/\Delta}$⇨$PhAM^{R26}Fth^{R26\Delta/\Delta}$ ($n = 3$), $Fth^{fl/fl}$⇨$PhAM^{R26}Fth^{R26\Delta/\Delta}$ ($n = 3$) chimeras. (**I**) Percentage and (**J**) median fluorescence intensity (MFI) of $LY6C^{High}$ monocyte-derived macrophages ($CD11b^+$, $F4/80^{Int}$, $LY6C^{Hi}$, Lin^-, $LY6G^-$, $CD11C^-$, $PhAM^+$) that are $PhAM^+$. Data in (**F**) is pooled from 2 independent experiments. Data in (**F**, **G**, **I**, **J**) is presented as individual values and mean. One-way ANOVA with Tukey's range test for multiple comparison correction was used for comparison between multiple groups. NS: non-significant, $^{**}P < 0.01$, $^{****}P < 0.0001$.

© The Author(s)
